# Performance of [^18^F]FDG PET/CT in Diagnosing Cyst Infections in Patients with Autosomal Dominant Polycystic Kidney Disease: A Systematic Review and a Bivariate Meta-Analysis

**DOI:** 10.3390/diagnostics14151603

**Published:** 2024-07-25

**Authors:** Giorgio Treglia, Domenico Albano, Alessio Rizzo, Antonio Bellasi, Andor W. J. M. Glaudemans, Olivier Gheysens

**Affiliations:** 1 Division of Nuclear Medicine, Imaging Institute of Southern Switzerland, Ente Ospedaliero Cantonale, 6500 Bellinzona, Switzerland; 2 Faculty of Biomedical Sciences, Università della Svizzera Italiana, 6900 Lugano, Switzerland; antonio.bellasi@eoc.ch; 3 Faculty of Biology and Medicine, University of Lausanne, 1011 Lausanne, Switzerland; 4 Division of Nuclear Medicine, ASST Spedali Civili Brescia, 25123 Brescia, Italy; domenico.albano@unibs.it; 5 Nuclear Medicine Department, University of Brescia, 25121 Brescia, Italy; 6 Division of Nuclear Medicine, Candiolo Cancer Institute, FPO-IRCCS, 10060 Turin, Italy; alessio.rizzo@ircc.it; 7 Service of Nephrology, Ospedale Regionale di Lugano, Ente Ospedaliero Cantonale, 6900 Lugano, Switzerland; 8 Medical Imaging Center, Department of Nuclear Medicine & Molecular Imaging, University of Groningen, University Medical Center Groningen, 9700-RB Groningen, The Netherlands; a.w.j.m.glaudemans@umcg.nl; 9 Department of Nuclear Medicine, Cliniques Universitaires Saint-Luc and Institute of Clinical and Experimental Research (IREC), Université Catholique de Louvain, 1200 Brussels, Belgium; olivier.gheysens@uclouvain.be

**Keywords:** PET/CT, FDG, nuclear medicine, hybrid imaging, cyst infection, ADPKD, cystic kidney diseases, infectious diseases

## Abstract

Background: Fluorine-18 fluorodeoxyglucose positron emission tomography/computed tomography ([^18^F]FDG PET/CT) has been suggested as a useful imaging method for diagnosing cyst infections in patients with autosomal dominant polycystic kidney disease (ADPKD). The aim of this article is to provide evidence-based data in this setting. Methods: A systematic literature review (exploring several bibliographic databases) and a bivariate meta-analysis were carried out to calculate the pooled diagnostic performance of [^18^F]FDG PET/CT in diagnosing probable cyst infection in ADPKD. Results: Ten studies (282 PET/CT scans and 249 patients) were included in the analysis. The pooled sensitivity and specificity of [^18^F]FDG PET/CT in this setting were 84.6% (95% confidence interval: 75.4–90.7) and 94.9% (95% confidence interval: 72.6–99.2), respectively, without statistical heterogeneity or significant publication bias. [^18^F]FDG PET/CT significantly changed patient management in more than half of ADPKD patients with suspected cyst infection. Conclusions: [^18^F]FDG PET/CT has high performance in diagnosing probable cyst infections in ADPKD patients with an impact on management in the majority of patients. Although more studies are warranted, the provided evidence-based data are an important step towards the integration of [^18^F]FDG PET/CT in clinical and diagnostic guidelines on probable cyst infection in ADPKD patients.

## 1. Introduction

Cystic kidney disease, when broadly defined, includes a wide spectrum of genetic, developmental or acquired diseases with the formation of cysts in one or both kidneys. Autosomal dominant polycystic kidney disease (ADPKD) is the most common inherited cystic kidney disease (common causative mutations involve the PKD1 and PKD2 genes) primarily affecting adults. However, renal cyst formation and expansion begin early in life [1,2]. Genetic testing provides a definitive diagnosis and prognostic information in patients with ADPKD, although kidney ultrasonography is still considered the modality of choice for ADPKD diagnosis [1,2]. Common clinical presentations of ADPKD in pediatric patients include multiple bilateral large cysts, hypertension and proteinuria. Conversely, adult patients may present with numerous bilateral cysts and enlarged kidneys, often leading to end-stage renal disease. Extra-renal features of ADKPD include liver cysts, cerebral aneurysms, cardiac valvular disease, colonic diverticulosis, hernias and male infertility [1,2].

Although ADPKD patients are often asymptomatic in the early stages of the disease, several complications may occur over time, such as hypertension (the most common complication), nephrolithiasis (as polycystic kidneys are prone to stasis) and internal hemorrhage in renal cysts. The incidence of cyst infections is 0.01 episode per year per patient and cyst infections are responsible for about 10% of hospital admissions in patients with ADPKD. Cyst infection is a severe complication of ADPKD that can lead to abscess formation, sepsis and death [3,4]. Therefore, accurate and early diagnosis is of the utmost importance for patient management. However, the diagnosis of cyst infections in ADPKD remains challenging due to their nonspecific clinical signs and biochemical parameters and the limited diagnostic performance of conventional imaging methods such as ultrasound, computed tomography (CT) and magnetic resonance imaging (MRI) [3,5]. More recently, hybrid imaging methods and, in particular, CT combined with positron emission tomography using fluorine-18 fluorodeoxyglucose as a radiotracer ([^18^F]FDG PET/CT), providing both anatomical and functional information related to glucose metabolism, has been suggested as a useful and promising tool in the diagnosis of cyst infections in patients with ADPKD [6,7]. The rationale for using [^18^F]FDG PET to detect cyst infections is based on the increased glucose metabolism of cells involved in infectious foci and the host response [8,9].

Currently, there are no published systematic reviews or meta-analyses on the diagnostic performance of [^18^F]FDG PET/CT for detecting cyst infection in patients with ADPKD. Therefore, this paper aims to provide evidence-based data in this setting that could be used in future clinical and imaging guidelines on cyst infection in ADPKD.

## 2. Materials and Methods

### 2.1. Protocol and Review Question

A predefined protocol was followed to perform this systematic review and meta-analysis of diagnostic test accuracy [10]. The last version of the Preferred Reporting Items for Systematic reviews and Meta-Analyses (PRISMA) checklist was used for our systematic review and meta-analysis report (PRISMA 2020 checklist reported as a Supplementary File S2) [11]. The protocol was prepared and followed but not registered as this is not mandatory (according to item no. 24 of the PRISMA checklist) [11].

The first step was the creation of the review question using the Population–Intervention–Comparison–Outcome (PICO) framework. The authors agreed on the following review question: “What is the diagnostic performance (“outcome”) of [^18^F]FDG PET/CT (“intervention”) in detecting cyst infections in patients with ADPKD (“population”) compared or not to other imaging methods (“comparison”)?”

All the authors defined the review question and related inclusion and exclusion criteria. To avoid selection bias for the systematic review, two review authors (G.T. and D.A.) independently performed the literature search, the study selection, the data extraction and the quality assessment of the retrieved studies. Possible disagreements were solved through the involvement of a third review author (A.R.).

### 2.2. Inclusion and Exclusion Criteria

According to the selected review question, the inclusion and exclusion criteria for the systematic review were defined. Only original studies (or subsets of studies) performing the selected index test ([^18^F]FDG PET/CT) in the defined target condition (patients with ADPKD and suspected cyst infection) were included. Articles were excluded if outside the topic of interest (regarding one or more elements of the PICO framework, e.g., articles regarding other imaging methods than [^18^F]FDG PET/CT), or if related to the topic of interest but not original studies (e.g., reviews, editorials, comments, letters). Even case reports or small case series (less than four patients) on the topic of interest were excluded due to the low quality of evidence of this type of article (which are also affected by publication and patient selection bias). Notably, type of publication language and time of publication were not considered among the exclusion criteria to increase the sensitivity of the literature search.

Studies included in the systematic review were also included in the statistical analysis (meta-analysis) if sufficient data were available to calculate the diagnostic performance of the index test and, if possible, to exclude overlap with other studies of the same group.

### 2.3. Literature Search and Study Selection

In terms of the literature search strategy, a comprehensive literature search using several electronic bibliographic databases (PubMed/MEDLINE, Embase, and Cochrane library) was performed. The search string was based on a combination of text words related to the index test and the target condition linked by Boolean operators (AND/OR): (a) “PET” OR “FDG” OR “positron” AND (b) “ADPKD” OR “polycystic” OR “renal cyst infection*” OR “kidney cyst infection*” OR “liver cyst infection*” OR “hepatic cyst infection*”. The search was updated until 31 May 2024. To increase the sensitivity of the literature search, the references of retrieved articles were also screened for additional eligible articles related to the review question.

The final selection of studies was performed through a careful examination of the titles and abstracts of all retrieved records according to the inclusion and exclusion criteria listed above.

### 2.4. Data Extraction and Collection

The full texts of the selected studies were retrieved. Data from selected articles were extracted from the full texts, figures and tables. These data were collected using predefined data collection forms. The collected data included basic information studies (author names, publication year, country, study design, funding sources), information on the patients (number of included subjects, gender and age), characteristics of the index test (PET device used, median time delay between antibiotic therapy initiation and PET/CT scan, radiopharmaceutical administered activity, uptake time, image analysis features, reference standard) and the diagnostic performance data of the index test on a scan-based analysis (including true/false positive findings, true/false negative findings, sensitivity, specificity, positive predictive value, negative predictive value and diagnostic accuracy). We contacted manuscript authors in case of relevant missing data for the meta-analysis.

### 2.5. Quality Appraisal of Included Studies

The quality assessment of the included articles was performed using a specific tool for diagnostic accuracy studies (QUADAS-2 tool). The risk of bias of the included studies was assessed regarding four areas: patient selection, index test, reference standard, and flow and timing. Applicability concerns were assessed regarding three areas: patient selection, index test and reference standard [12].

### 2.6. Statistical Analysis

Pooled sensitivity and specificity, pooled positive and negative likelihood ratios (LR+ and LR−) and pooled diagnostic odds ratio (DOR) of [^18^F]FDG PET/CT were calculated on a scan-based analysis using a bivariate random-effects model (taking into account the reference standard used in the included studies). This is a hierarchical statistical method suggested for diagnostic test accuracy meta-analyses. Compared to the univariate statistical approach, bivariate meta-analyses take into account the possible correlations among sensitivity and specificity [10]. The results of the meta-analysis are presented as pooled measures with 95% confidence intervals (95%CIs). Forest plots are not provided as this is a bivariate meta-analysis and not a univariate meta-analysis exploring single metrics independently. A summary receiver operating characteristics (SROC) curve is also used for displaying the results of the meta-analysis related to the diagnostic performance of [^18^F]FDG PET/CT. Heterogeneity/inconsistency was estimated by using the I-square index (I^2^) and publication bias was assessed through Egger’s test [10]. In case of significant heterogeneity among the studies, subgroup analyses taking into account several variables were planned to explore this heterogeneity.

Statistical analyses were performed using Meta-DiSc 2.0, a web application for meta-analyses of diagnostic test accuracy data (www.metadisc.es) [13].

## 3. Results

### 3.1. Literature Search Results

**Figure 1:**
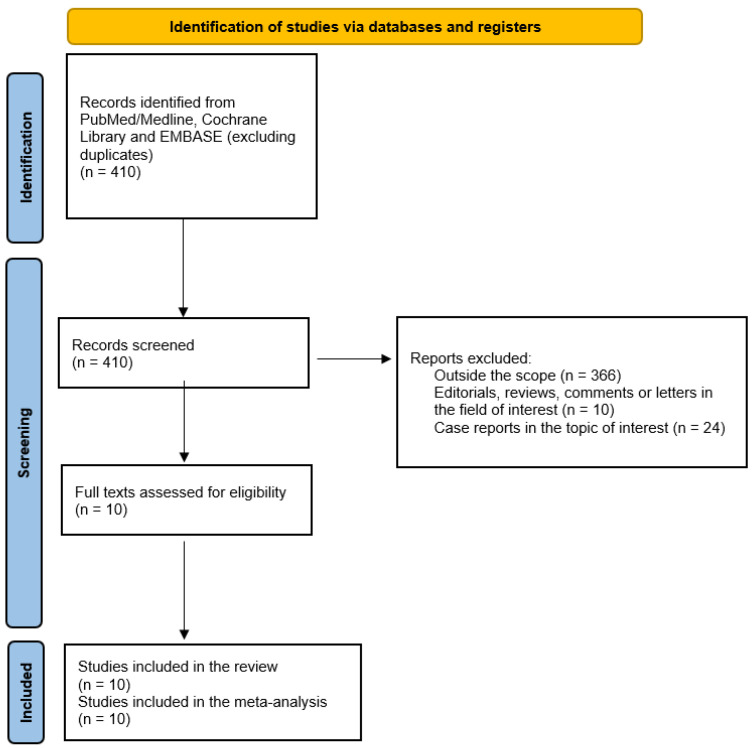
Summary of the process for the selection of eligible articles.

A comprehensive literature search of the selected bibliographic databases, after the exclusion of duplicates, yielded 410 records. The list of retrieved records is provided as a Supplementary File S1. After reading the titles and abstracts, 366 records were excluded for being outside the field of interest; 10 records were excluded for being editorials, reviews, comments or letters related to the selected topic; and 24 records were excluded for being case reports or small case series (less than four patients). The full texts of 10 original studies (or subsets of studies) were screened, and all of them were selected for inclusion in this systematic review and bivariate meta-analysis [14,15,16,17,18,19,20,21,22,23] (Figure 1). No additional manuscripts were added after cross-checking the references of the selected records. There were no disagreements among the review authors on the selected studies.

### 3.2. Characteristics of Included Studies and Patients

Ten articles (nine retrospective studies and one prospective study) published between 2009 and 2023 were included containing 282 [^18^F]FDG PET/CT scans performed in 249 ADPKD patients with suspected cyst infections. The studies were performed in European countries in 90% of cases. The median/mean age and the sex ratio of included patients mildly varied among the included studies. The specific details of included studies and patient characteristics are reported in Table 1.

### 3.3. Characteristics of the Index Test

[^18^F]FDG PET/CT was performed as a hybrid imaging method in all included studies using different tomographs. The mean injected [^18^F]FDG activity varied across studies, but the time from injection to PET/CT image acquisition was similar in all studies (around 60 min). Co-registered CT scans were performed as low-dose CT without the injection of iodinated contrast agents. [^18^F]FDG PET/CT image analysis was based on a visual assessment in all included studies and a semi-quantitative analysis using the maximum standardized uptake value (SUV_max_) was used in four studies (Table 2). In terms of PET image analysis, two patterns were considered positive for cyst infection: increased (homogeneous or heterogeneous; focal or multifocal) [^18^F]FDG uptake lining the cyst (in contrast to physiological uptake in the adjacent parenchyma) and diffuse [^18^F]FDG uptake within the cyst (after exclusion of cyst hemorrhage on CT). Recently, a four-point visual grading scale was suggested to evaluate suspected infected cysts (score 1: [^18^F]FDG uptake around the cyst ≤ mediastinal blood pool; score 2: uptake around the cyst > mediastinal blood pool but ≤ liver uptake; score 3: uptake around the cyst slightly > liver uptake; score 4: uptake around the cyst largely > liver uptake). Scores of 3 and 4 are considered suggestive of cyst infection. All extra-cystic sites of abnormal [^18^F]FDG uptake were also evaluated for additional sites of inflammation or infection. Furthermore, co-registered CT images were carefully analyzed (with a special focus on cyst wall thickness and/or adjacent fat infiltration and the densitometric characteristics of the cystic content).

### 3.4. Reference Standard

When reported, the reference standard for cyst infection in the included studies was based on conventionally accepted clinical criteria. Diagnosis of cyst infection was considered “definite” if confirmed by pus drainage (cyst aspiration showing neutrophils and/or microorganisms) [14,15,16,17,18,19,20,21,22,23]. For the diagnosis of “probable” cyst infections, two different criteria were used in the included studies: (A) the presence of all of the following conditions: fever (temperature ≥38 °C), abdominal pain, and increased plasma C-reactive protein levels ≥ 70 mg/L, the absence of other causes of inflammation and a favorable outcome after ≥21 days of antibiotic therapy [14,16]; and (B) the concurrent manifestation of these conditions: fever (temperature of >38.5 °C for 3 days), abdominal tenderness in the kidney or liver area, increased plasma C-reactive protein (>50 mg/L) and the absence of CT arguments for recent intracystic bleeding or other causes of fever [15,17,18,19,20,21,23].

### 3.5. Quality Assessment

**Figure 2:**
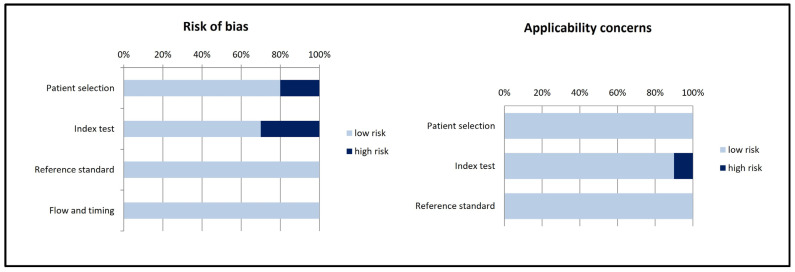
Summary of the quality assessment of the included studies using the QUADAS-2 tool.

The results of the quality assessment using the QUADAS-2 tool are illustrated in Figure 2.

### 3.6. Main Findings of the Included Studies (Qualitative Synthesis)

Overall, the included studies reported a good performance of [^18^F]FDG PET/CT in diagnosing both liver and renal probable cyst infections in patients with ADPKD [14,15,16,17,18,19,20,21,22,23]. Cyst infections detected by [^18^F]FDG PET/CT were predominantly multifocal [14,15,16,17,18,19,20,21,22,23]. Diagnostic outcomes on a scan-based analysis are reported in Table 3.

When using a four-point visual score for PET image analysis, a score of ≥3 was associated with a significantly higher risk of cyst infection compared to scores of 1 and 2. The four-point visual score also showed a good inter-observer agreement and inter-rater reliability [14,16]. In particular, applying a visual threshold of ≥3 improved the specificity of [^18^F]FDG PET/CT without changes in sensitivity [16].

Compared to conventional imaging methods (ultrasound, CT, MRI), [^18^F]FDG PET/CT showed a higher diagnostic performance in detecting cyst infection in ADPKD patients [17,19,20,21,22,23]. In particular, CT was reported to be significantly inferior to [^18^F]FDG PET/CT in terms of both sensitivity and negative predictive value in this setting [19,20]. Few comparative data are available with MRI [17,20].

[^18^F]FDG PET/CT was also useful to detect or rule out extra-cystic causes of infection or inflammation in ADPKD patients with suspected cyst infection [14,15,16,17,18,19,20,21]. Interestingly, the total duration of hospital stay and the duration between the PET/CT scan and discharge from the hospital were significantly longer for patients with positive [^18^F]FDG PET/CT compared to patients with negative [^18^F]FDG PET/CT [17]. Creatinine levels were significantly higher in patients with [^18^F]FDG PET/CT findings suggestive of cyst infection [17]. It is worthwhile to mention that long-term antibiotic treatment before [^18^F]FDG PET/CT may impact its diagnostic performance due to false negative findings [19]. Notably, there was a variable time delay between the initiation of antibiotic therapy and the performance of the [^18^F]FDG PET/CT scan in the selected studies [14,15,16,17,18,19,20,21,22,23].

In addition to diagnostic performance, some included studies also explored the possible role of [^18^F]FDG PET/CT in monitoring cyst infections or evaluating treatment response but, due to the limited available data, the usefulness of [^18^F]FDG PET/CT for these indications is still controversial and warrants further investigation [19,20,22].

Overall, [^18^F]FDG PET/CT significantly changed patient management in more than half of ADPKD patients with suspected cyst infection [14,17,18,22]. More specifically, a negative [^18^F]FDG PET/CT scan led to significant modifications of therapeutics in about half of the cases, while a positive [^18^F]FDG PET/CT finding for cyst infection significantly changed treatment, mainly with regard to the duration of antibiotics, in most of the cases [17,18]. Positive [^18^F]FDG PET/CT findings related to a non-cystic inflammation/infection also changed patient management in most of the cases [14,18].

### 3.7. Meta-Analysis (Quantitative Synthesis)

**Figure 3:**
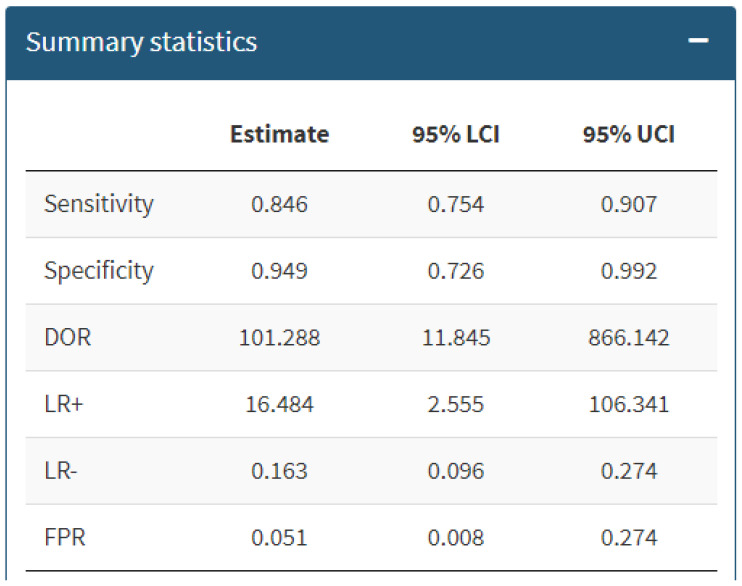
Summary statistics about the bivariate meta-analysis on the performance of [^18^F]FDG PET/CT in diagnosing cyst infection in ADPKD patients. DOR = diagnostic odds ratio; LR+/− = positive and negative likelihood ratio; FPR = false positive rate; 95% LCI/UCI = lower/upper 95% confidence interval.

**Figure 4:**
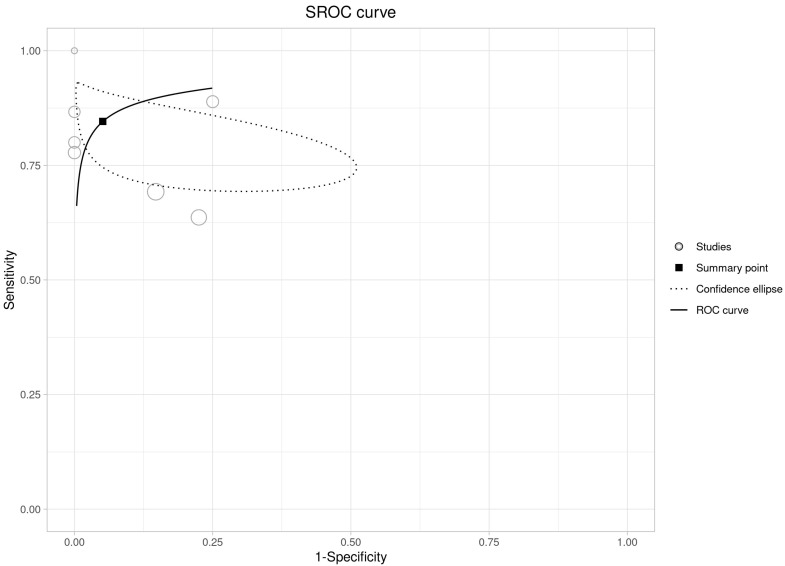
Summary ROC curve on the performance of [^18^F]FDG PET/CT in diagnosing cyst infection in ADPKD patients. This is a plot of the false positive rate (x-axis) versus the true positive rate (y-axis). The more that the summary ROC curve hugs the top left corner of the plot, the better the model at classifying the data into categories.

The bivariate meta-analysis of ten studies including 282 [^18^F]FDG PET/CT scans performed for suspected cyst infection in ADPKD patients [14,15,16,17,18,19,20,21,22,23] resulted in a pooled sensitivity and a pooled specificity of 84.6% (95%CI: 75.4–90.7) and 94.9% (95%CI: 72.6–99.2), respectively. The pooled DOR, LR+ and LR− were 101 (95%CI: 11.8–866), 16.5 (95%CI: 2.5–106.3) and 0.163 (0.096–0.274), respectively. Summary statistics are displayed in Figure 3, whereas the SROC curve is illustrated in Figure 4.

There was no significant statistical heterogeneity among the studies included in this analysis, as the inconsistency index was 0%. Due to these results on statistical heterogeneity, subgroup analyses to explore the heterogeneity were not performed. Egger’s test did not demonstrate a significant publication bias (*p* = 0.64).

## 4. Discussion

[^18^F]FDG PET/CT is currently the imaging method of choice for a wide range of inflammatory and infectious diseases due to the increased [^18^F]FDG uptake in these processes, its widespread availability and its ease of use in combination with excellent sensitivity. To the best of our knowledge, this is the first systematic review and meta-analysis exploring the performance of [^18^F]FDG PET/CT in diagnosing cyst infections in patients with ADPKD. Our systematic review and bivariate meta-analysis clearly demonstrate that [^18^F]FDG PET/CT achieved high performance in diagnosing probable renal or hepatic cyst infections in patients with ADPKD [14,15,16,17,18,19,20,21,22,23].

**Figure 5:**
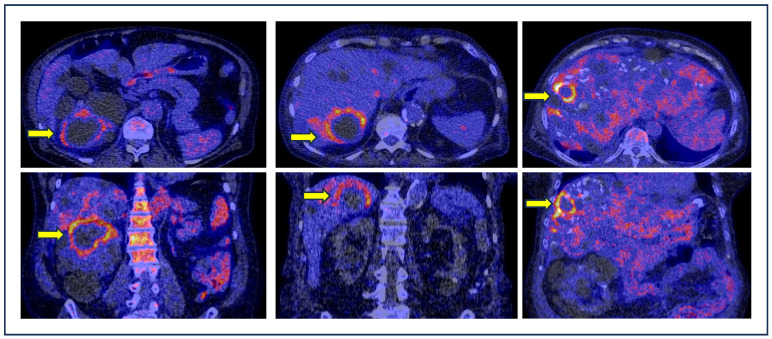
Examples of kidney or liver cyst infections (yellow arrows) detected by [^18^F]FDG PET/CT in ADPKD patients. Axial (**top**) and coronal (**bottom**) fused PET/CT images demonstrate high [^18^F]FDG uptake around the infected cysts.

Cyst infection is a frequent and severe complication in patients with ADPKD that can lead to abscess formation, sepsis and death. Moreover, cyst infection in ADPKD patients remains an important cause of hospitalization. Therefore, an early and correct diagnosis of cyst infection is of high clinical relevance [23,24,25,26]. Several studies explored the use of [^18^F]FDG PET/CT to identify cyst infections in ADPKD patients with promising results [7,14,15,16,17,18,19,20,21,22,23]. Some examples of kidney and liver cyst infections in patients with ADPKD detected by [^18^F]FDG PET/CT are shown in Figure 5.

The articles included in this systematic review and meta-analysis highlight the need to combine both clinical and PET criteria for an optimal diagnostic approach [14]. Furthermore, [^18^F]FDG PET/CT may detect extra-cystic inflammatory or infectious lesions, enhancing its role and impact on the management of patients with ADPKD and suspected infection [14,15,16,17,18,19,20,21].

False negative findings of [^18^F]FDG PET/CT in cyst infections could be explained by a long-term antibiotic treatment before performing the [^18^F]FDG PET/CT, which may reduce its sensitivity [14,19]. For this reason, some authors recommend performing [^18^F]FDG PET/CT within 7 days of antibiotic initiation in ADPKD patients with suspected cyst infection [18,19]. Conversely, [^18^F]FDG PET/CT can also result in a false positive diagnosis of cyst infection, in particular in more complicated cysts where increased tracer uptake may depict physiological urinary uptake [7].

Standardization of [^18^F]FDG PET image interpretation is important and the recent introduction of a visual four-point grading scale may further improve the diagnosis of cyst infections in patients with ADPKD. This visual scoring system is highly reproducible between observers given its high specificity and negative predictive value. A semi-quantitative score > 3 seems to be the most specific PET metric for cyst infections [14,16]. However, the use of semi-quantitative metrics needs further investigation.

Compared to conventional imaging methods (ultrasound, CT and MRI), [^18^F]FDG PET/CT showed a better diagnostic performance in detecting cyst infection in ADPKD [17,19,20,21,22,23]. A diagnosis of infection with conventional imaging methods is mainly based on wall thickening and the heterogeneous content of the cysts. However, due to anatomical consequences related to the presence of multiple cysts in ADPKD, it is hard to distinguish infected from non-infected complicated cysts in ADPKD through conventional imaging methods [7,27]. Furthermore, iodinated contrast agents for CT and gadolinium-based contrast agents for MRI are contraindicated in ADPKD patients with impaired renal function due to the potential risks of nephrotoxicity and nephrogenic systemic fibrosis, respectively [7]. Compared to [^18^F]FDG PET/CT, CT was reported to be significantly inferior in terms of both sensitivity and negative predictive value in detecting cyst infection [19]. Even if a single retrospective study reported a superior performance of [^18^F]FDG PET/CT compared to MRI in diagnosing cyst infection in ADPKD patients [20], more head-to-head comparison studies are needed. Abdominal MRI was proposed as a potentially sensitive method for detecting cyst infections, in particular because of its high lesion-to-background contrast using diffusion-weighted sequences (DWIs) [28]. However, unlike [^18^F]FDG PET/CT, abdominal MRI does not provide whole-body information to rule out or detect other infectious foci and the imaging procedure can be time-consuming and less tolerated by all patients [17]. To date, there are no studies evaluating the role of hybrid [^18^F]FDG PET/MRI in ADPKD patients. PET/MRI has a relatively limited availability compared to PET/CT, but combining the high soft tissue contrast and high resolution with the molecular/metabolic data provided by PET in one single examination could potentially positively impact the management of infectious diseases, including cyst infections in ADPKD patients [29].

Overall, [^18^F]FDG PET/CT findings (positive or negative) significantly changed patient management in more than half of ADPKD patients with suspected cyst infection [14,17,18,22], mainly with regard to the type and duration of antibiotic treatment. In addition to changes in antibiotic treatment, [^18^F]FDG uptake in infected cysts could also guide clinicians to perform interventional procedures to drain certain cysts [7].

Additionally, [^18^F]FDG PET/CT was helpful in the diagnostic work-up of the majority of ADPKD patients (65%) with febrile abdominal pain, including non-cystic inflammations [14]. Positive [^18^F]FDG PET/CT findings corresponding to a non-cystic inflammation changed patient management in most of the cases [14,18].

Unfortunately, the limited available data do not support the use of [^18^F]FDG PET/CT to evaluate the treatment failure of cyst infections in ADPKD patients; however, its value in monitoring early treatment response needs to be further explored [19,20,22,30].

We excluded articles on PET/CT using white blood cells labeled with [^18^F]FDG (WBC-PET/CT) from our analysis, the reason being that radiolabeled white blood cells with [^18^F]FDG have a different targeting mechanism compared to [^18^F]FDG. From a theoretical point of view, [^18^F]FDG-labeled white blood cells, similar to ^99m^Technetium-labeled white blood cells, may provide a better specificity for diagnosing infections [31]. However, limited experience is available with only one study evaluating the performance of WBC-PET/CT for the diagnosis of cyst infection in ADPKD patients, reporting a good diagnostic performance (sensitivity 85.7% and specificity 87.5%) and added value compared to CT and MRI [32]. However, the results of this single study are not sufficient for justifying the clinical use of WBC-PET/CT in this setting.

The limitations of our analysis include the retrospective nature of the majority of the included studies, for which a potential selection bias should be considered [33,34]. In particular, a clear selection bias was present in the paper of Sallée et al. [23]. Furthermore, a suboptimal but clinically acceptable and well-established reference standard was used in the included studies. Cyst puncture and pus drainage is currently the only true gold diagnostic standard for cyst infection, but it may lead to serious complications and is not frequently performed [7]. Even if the PET/CT-guided percutaneous puncture of an infected cysts in ADPKD could be performed [35], unfortunately, only a few cases of suspected cyst infection underwent aspiration of pus and a subgroup analysis with the results of the index test on this specific subgroup was not feasible. Lastly, a subgroup analysis comparing the diagnostic performance of the index test in detecting cyst infection in transplanted versus non-transplanted kidney patients could be useful since immunosuppression can affect the clinical scenario [15,36,37]; this subgroup analysis was not performed due to the insufficient retrieved individual data of both patient groups. However, due to the absence of statistical heterogeneity in our analysis, it is likely that the diagnostic performance of [^18^F]FDG PET/CT in detecting cyst infection would be similar in both groups.

A prospective, preferably multi-center, trial using pus drainage and culture as the gold standard to confirm cyst infection could help to better define both the PET imaging criteria and the clinical criteria of cyst infection. Such a trial could also be useful to develop a diagnostic scoring system combining clinical and imaging criteria. Cost-effectiveness studies and studies demonstrating the impact of [^18^F]FDG PET/CT on the outcome of ADPKD patients with suspected cyst infection are warranted. We are aware of the relatively higher costs of [^18^F]FDG PET/CT compared to other conventional diagnostic imaging methods [38,39]. However, it is likely that performing [^18^F]FDG PET/CT early in this clinical setting may reduce overall healthcare costs in ADPKD patients [17,40].

## 5. Conclusions

[^18^F]FDG PET/CT has a high diagnostic performance in ADPKD patients with suspected cyst infections and is important for treatment decision making in the majority of patients. Despite the fact that large prospective studies, cost-effectiveness analyses and studies on the impact of [^18^F]FDG PET/CT findings on the outcome of ADPKD patients with suspected cyst infection are warranted, the provided evidence-based data should support the inclusion of [^18^F]FDG PET/CT in clinical and diagnostic guidelines on cyst infection in ADPKD patients.

## Figures and Tables

**Table 1:** Characteristics of included studies and patients.

Authors	Year	Country	Study Design	Funding	No. of Patients with Suspicious Cyst Infections Performing [^18^F]FDG PET/CT (No. of PET/CT Scans)	Male %	Median/Mean Age
Demuynck et al. [14]	2023	Belgium	R	None	51 (51)	59%	Median: 57
Ronsin et al. [15]	2022	France	R	None	16 (16)	NR	NR
Neuville et al. [16]	2021	Belgium	R	None	38 (60)	53%	Mean: 59
Pijl et al. [17]	2018	Netherlands	R	None	30 (30)	50%	Median: 61
Neuville et al. [18]	2016	Belgium	R	None	28 (28)	57%	Mean: 56
Bobot et al. [19]	2016	France	R	None	24 (32)	46%	Mean: 59
Balbo et al. [20]	2014	Brazil	R	None	20 (20)	NR	Median: 51
Jouret et al. [21]	2011	Belgium	R	None	24 (27)	33%	NR
Piccoli et al. [22]	2011	Italy	P	None	10 (10)	30%	Mean: 69
Sallée et al. [23]	2009	France	R	None	8 (8)	NR	NR

Legend: NR = not reported; P = prospective; R = retrospective.

**Table 2:** Characteristics of the index test.

Authors	PET/CT Tomograph (Manufacturer)	Median Time Delay between Antibiotic Initiation and PET/CT	[^18^F]FDG Mean Injected Activity	Uptake Time (Minutes)	Image Analysis	Semiquantitative Parameters
Demuynck et al. [14]	Biograph True-Point 40 or Biograph 16 (Siemens) or Discovery MI4 (GE)	9 days	4–4.25 MBq/kg	60	Visual (four-point scale)	Not used
Ronsin et al. [15]	NR	NR	NR	NR	Visual	Not used
Neuville et al. [16]	Gemini TF (Philips)	9 days	3 MBq/kg	60	Visual (four-point scale)	Not used
Pijl et al. [17]	Biograph mCT (Siemens)	NR	3 MBq/kg	60	Visual	Not used
Neuville et al. [18]	NR	7 days	NR	NR	Visual	Not used
Bobot et al. [19]	NR	7 days	4 MBq/kg	60	Visual and semi-quantitative	SUV_max_
Balbo et al. [20]	Biograph 2 (Siemens) or Discovery 690 (GE)	NR	370 MBq	60–90	Visual and semi-quantitative	SUV_max_
Jouret et al. [21]	Gemini TF (Philips)	NR	300 MBq	NR	Visual and semi-quantitative	SUV_max_
Piccoli et al. [22]	Discovery ST (GE)	NR	3 MBq/kg	60	Visual and semi-quantitative	SUV_max_
Sallée et al. [23]	NR	NR	NR	NR	Visual	Not used

Legend: NR = not reported; SUV_max_ = maximal standardized uptake value.

**Table 3:** Diagnostic outcomes of [^18^F]FDG PET/CT in detecting probable cyst infections in ADPKD patients on a scan-based analysis.

Authors	TP	FP	TN	FN	Sens.	Spec.	PPV	NPV	Acc.
Demuynck et al. [14]	7	9	31	4	63.6%	77.5%	43.7%	88.6%	74.5%
Ronsin et al. [15]	14	0	0	2	87.5%	NC	100%	NC	87.5%
Neuville et al. [16]	18	5	29	8	69.2%	85.3%	78.3%	78.4%	78.3%
Pijl et al. [17]	16	3	9	2	88.9%	75%	84.2%	81.8%	83.3%
Neuville et al. [18]	8	0	18	2	80%	100%	100%	90%	92.9%
Bobot et al. [19]	14	0	14	4	77.8%	100%	100%	77.8%	87.5%
Balbo et al. [20]	19	0	0	1	95%	NC	100%	NC	95%
Jouret et al. [21]	13	0	12	2	86.7%	100%	100%	85.7%	92.6%
Piccoli et al. [22]	6	0	4	0	100%	100%	100%	100%	100%
Sallée et al. [23]	8	0	0	0	100%	NC	100%	NC	100%

Legend: Acc. = diagnostic accuracy; FN = false negative; FP = false positive; NC = not calculable; NPV = negative predictive value; PPV = positive predictive value; Sens. = sensitivity; Spec. = specificity; TN = true negative; TP = true positive.

## Data Availability

The original data presented in the study are available from consulting bibliographic databases (PubMed/Medline, Cochrane Library, EMBASE).

## Supplementary Materials

The following supporting information can be downloaded at: https://www.mdpi.com/article/10.3390/diagnostics14151603/s1, File S1: List of retrieved records through the literature search. File S2: PRISMA 2020 checklist.
