# Supplementary material for: Performance of [18F]FDG PET/CT in Diagnosing Cyst Infections in Patients with Autosomal Dominant Polycystic Kidney Disease: A Systematic Review and a Bivariate Meta-Analysis
Source: Diagnostics (Basel). 2024 Jul 25;14(15):1603. doi: 10.3390/diagnostics14151603 (PMC11312050; doi:10.3390/diagnostics14151603)
Supplement: Supplementary file 1 [file diagnostics-14-01603-s001.zip › File S1 - list of retrieved records.pdf]

Supplemental file S1: list of retrieved records

Search question:
Performance of [^{18}F]FDG PET/CT in diagnosing cyst infections in patients with ADPKD
Search string (adapted to several databases):
((PET) OR (FDG) OR (positron)) AND ((ADPKD) OR (polycystic) OR (renal cyst infection*) OR (kidney cyst infection*) OR (liver cyst infection*) OR (hepatic cyst infection*))
Data of last search: 31 May 2024
Selected databases: PubMed/Medline, Cochrane library, EMBASE
Records retrieved excluding duplicates: n = 410

Original articles related to the review question n =10
Case reports related to the review question n = 24
Reviews, comments, letters or editorials related to the review question n = 10
Outside the field of interest n = 366

List of retrieved records (duplicates excluded):

1: Poxleitner M, Hoffmann SHL, Berezhnoy G, Ionescu TM, Gonzalez-Menendez I, Maier FC, Seyfried D, Ehrlichmann W, Quintanilla-Martinez L, Schmid AM, Reischl G, Trautwein C, Maurer A, Pichler BJ, Herfert K, Beziere N. Western diet increases brain metabolism and adaptive immune responses in a mouse model of amyloidosis. J Neuroinflammation. 2024 May 14;21(1):129. doi: 10.1186/s12974-024-03080-0. PMID: 38745337; PMCID: PMC11092112.

2: Zeng XT, Liang X, Hong ZL, Chen S, Yang JC, Lin YC, Wu SS. Initial investigation on ultrasound-guided percutaneous biopsy of lesions in the first hepatic hilum with fusion of ultrasound and multimodal imaging cognitive guidance. Front Oncol. 2024 Apr 24;14:1297153. doi: 10.3389/fonc.2024.1297153. PMID: 38720805; PMCID: PMC11077297.

3: Valverde de Morales HG, Wang HL, Garber K, Corces V, Li H. <i>CTCF</i>-Related Disorder. 2024 Apr 25. In: Adam MP, Feldman J, Mirzaa GM, Pagon RA, Wallace SE, Bean LJH, Gripp KW, Amemiya A, editors. GeneReviews® [Internet]. Seattle (WA): University of Washington, Seattle; 1993–2024. PMID: 38662876.

4: Bjornstad P, Richard G, Choi YJ, Nowak KL, Steele C, Chonchol MB, Nadeau KJ, Vigers T, Pyle L, Tommerdahl K, van Raalte DH, Hilkin A, Driscoll L, Birznieks C, Hopp K, Wang W, Edelstein C, Nelson RG, Gregory AV, Kline TL, Blondin D, Gitomer B. Kidney Energetics and Cyst Burden in Autosomal Dominant Polycystic Kidney Disease: A Pilot Study. Am J Kidney Dis. 2024 Apr 13:S0272-6386(24)00716-9. doi: 10.1053/j.ajkd.2024.02.016. Epub ahead of print. PMID: 38621633.

5: Teranishi N, Inagaki S, Yoshida H, Nagasawa S, Takane Y, Yan M, Takahashi M, Masubuchi M, Wakabayashi K, Uetake H, Itoh Y. [Long-Term Suppression with First-Line Chemotherapy(FOLFIRI plus BV)for Peritoneal Metastasis]. Gan To Kagaku Ryoho. 2024 Feb;51(2):199-201. Japanese. PMID: 38449412.

6: Xu S, Huang B, Cao Y, Zhong Z, Yin J. Polycystic intrahepatic infection caused by Enterococcus casseliflavus: a case report and literature review. BMC Nephrol. 2024 Mar 6;25(1):88. doi: 10.1186/s12882-024-03531-z. PMID: 38448824; PMCID: PMC10919048.

7: Girme A, Gupta V. A Case Report of the Coexistence of Gastric Cancer With Polycystic Kidney and Liver Disease: Unveiling the Complexity. Cureus. 2024 Feb 4;16(2):e53574. doi: 10.7759/cureus.53574. PMID: 38445116; PMCID: PMC10914406.

8: Albano D, Calabrò A, Dondi F, Bagnasco S, Tucci A, Bertagna F. The role of baseline 2-[¹⁸ F]-FDG-PET/CT metrics and radiomics features in predicting primary gastric lymphoma diagnosis. Hematol Oncol. 2024 Mar;42(2):e3266. doi: 10.1002/hon.3266. PMID: 38444261.

9: Chandra P, Sethi A, Chinnappan S. Malignant Portal Vein Thrombosis with No Obvious Liver Parenchymal Mass on Fluorodeoxyglucose Positron Emission Tomography/Computed Tomography. Indian J Nucl Med. 2023 Oct-Dec;38(4):404-406. doi: 10.4103/ijnm.ijnm_15_23. Epub 2023 Dec 20. PMID: 38390545; PMCID: PMC10880848.

10: Huang Z, Zou S, Liu Q, Qi W, Sharma A, Wang Y, Jin A, Schmidt-Wolf IGH, Lu P, Ai W, Liao F. Inferring the diagnostic potential of 18F-FDG-PET/CT in post-renal transplantation from a unique case harboring multiple rare complications. Front Med (Lausanne). 2024 Feb 2;11:1353466. doi: 10.3389/fmed.2024.1353466. PMID: 38371509; PMCID: PMC10869483.

11: Furuta A, Oura S, Shintani H, Kataoka N, Tanaka H, Takamatsu S, Ono W. Focal coagulative necrosis of the liver in a patient with sustained virologic response to anti-hepatitis C virus therapy. Radiol Case Rep. 2024 Jan 22;19(4):1514-1518. doi: 10.1016/j.radcr.2024.01.035. PMID: 38304350; PMCID: PMC10830427.

12: Yu F, Wang J, Ke Z, Zhang Y, Xu L, Zhang H, Huang K, Cheng F, Yang H, Wang L, Wang Z, Shou L, Yu W, Fang H, Medeiros LJ, Wang W. EBV-positive Nodal T-Cell and NK-Cell Lymphoma: A Study of 26 Cases Including a Subset With Strong CD30 Expression Mimicking Anaplastic Large Cell Lymphoma. Am J Surg Pathol. 2024 Apr 1;48(4):406-416. doi: 10.1097/PAS.0000000000002184. Epub 2024 Jan 30. PMID: 38287746.

13: Rumrill WM, Ballard DH. Metastatic Renal Cell Carcinoma in a Patient With Autosomal Dominant Polycystic Kidney Disease. Clin Nucl Med. 2024 Mar

1;49(3):e131-e133. doi: 10.1097/RLU.0000000000005059. Epub 2024 Jan 23. PMID: 38271253.

14: Luo S, Wang X, Ren X, Cheng Y, Guo L, Yan P, Lv J, Su X, Shen J, Zhao K, Sun K, Chen J, Wang R. A case of TM infection with challenging differential diagnosis from lymphoma post-renal transplant. BMC Infect Dis. 2023 Dec 19;23(1):888. doi: 10.1186/s12879-023-08912-7. PMID: 38114917; PMCID: PMC10729359.

15: Moideen A, Singh H, Kumar R, Naik SM, Gupta A, Sekar A, Nada R. 18 F-FDG PET/CT in Renal Allograft Tuberculosis Mimicking as Posttransplant Malignancy. Clin Nucl Med. 2024 Feb 1;49(2):e52-e53. doi: 10.1097/RLU.0000000000004977. Epub 2023 Dec 4. PMID: 38049975.

16: Demuynck S, Lovinfosse P, Seidel L, Jentjens S, Mekahli D, Jouret F, Bammens B, Goffin K. Standardized 4-point scoring scale of [¹⁸F]-FDG PET/CT imaging helps in the diagnosis of renal and hepatic cyst infections in patients with autosomal dominant polycystic kidney disease: a validation cohort. Clin Kidney J. 2023 Jul 5;16(12):2542-2548. doi: 10.1093/ckj/sfad159. PMID: 38046039; PMCID: PMC10689132.

17: Kosaka S, Shimizu S, Nakayamada S, Nawata A, Shimono N, Tanaka M, Maruyama H, Osada Y, Tanaka Y. A delayed diagnosis of fascioliasis: The importance of appropriate fecal diagnostic method. J Infect Chemother. 2024 May;30(5):454-458. doi: 10.1016/j.jiac.2023.11.006. Epub 2023 Nov 8. PMID: 37944698.

18: Buttenschoen J, Pavel V, Mehrl A, Michels B, Albaladejo Fuertes S, Seydel B, Schlosser-Hupf S, Müller M, Schmid S. Bacterial Infection of an Alveolar Echinococcus Cyst from <i>C. perfringens</i> Septicemia: A Case Report and Review of the Literature. Medicina (Kaunas). 2023 Oct 13;59(10):1828. doi: 10.3390/medicina59101828. PMID: 37893546; PMCID: PMC10608314.

19: Kim Y, Li C, Gu C, Fang Y, Tycksen E, Puri A, Pietka TA, Sivapackiam J, Kidd K, Park SJ, Johnson BG, Kmoch S, Duffield JS, Bleyer AJ, Jackrel ME, Urano F, Sharma V, Lindahl M, Chen YM. MANF stimulates autophagy and restores mitochondrial homeostasis to treat autosomal dominant tubulointerstitial kidney disease in mice. Nat Commun. 2023 Oct 14;14(1):6493. doi: 10.1038/s41467-023-42154-0. PMID: 37838725; PMCID: PMC10576802.

20: Yin Y, Liu J, Sun R, Liu X, Zhou Z, Zhang H, Li D. Exploring the efficacy of ¹⁸F-FDG PET/CT in hepatocellular carcinoma diagnosis: role of Ki-67 index and tumor differentiation. Abdom Radiol (NY). 2023 Nov;48(11):3408-3419. doi: 10.1007/s00261-023-04027-4. Epub 2023 Sep 8. PMID: 37682282; PMCID: PMC10556170.

21: Tang Y, Chen X, Lu X, Yuan Z, Yang Y, Qiu C, Li H. Case Report: Primary hepatic neuroendocrine tumor: two cases report with literature review. Front Oncol. 2023 Aug 4;13:1225583. doi: 10.3389/fonc.2023.1225583. PMID: 37601674; PMCID: PMC10436565.

22: Van Den Noortgate R, Kiselinova M, Sys C, Accou G, Laureys G, Van Vlierberghe H, Berrevoet F, Kreps EO. Concurrent Ocular and Cerebral Toxoplasmosis in a Liver Transplant Patient Treated with Anti-CD40 Monoclonal Antibody. Case Rep Infect Dis. 2023 Jul 27;2023:5565575. doi: 10.1155/2023/5565575. PMID: 37545749; PMCID: PMC10400299.

23: Elgazar A, Awad AK, Rashed AM, Kelany M, Saber HS. Pleomorphic rhabdomyosarcoma of the liver in an adult: An extremely rare case report and review of literature. Int J Surg Case Rep. 2023 Aug;109:108585. doi: 10.1016/j.ijscr.2023.108585. Epub 2023 Jul 28. PMID: 37541015; PMCID: PMC10412826.

24: Maggioni G, Bonis A, Schiavon M, Giraudo C, Lunardi F, Pezzuto F, Calabrese F. An unexpected guest: Pulmonary echinococcosis diagnosed by intraoperative frozen section examination. A case report and literature review. Pathol Res Pract. 2023 Aug;248:154615. doi: 10.1016/j.prp.2023.154615. Epub 2023 Jun 12. PMID: 37343377.

25: Sarswat S, Khan D, Sagar S, Kumar R. Role of 18 F-FDG PET/CT in the diagnosis of benign vs. malignant tumor thrombus. Nucl Med Commun. 2023 Aug 1;44(8):726-731. doi: 10.1097/MNM.0000000000001708. Epub 2023 Jun 5. PMID: 37272295.

26: Higuchi T, Hartrampf PE, Buck AK, Pomper MG, Rowe SP, Serfling SE, Werner RA. Role of Functional SPECT and PET in Renal Emergencies. Semin Nucl Med. 2023 Nov;53(6):786-796. doi: 10.1053/j.semnuclmed.2023.04.003. Epub 2023 May 24. PMID: 37236903.

27: Zhang X, Basuli F, Shi ZD, Shah S, Shi J, Mitchell A, Lai J, Wang Z, Hammoud DA, Swenson RE. Synthesis and Evaluation of Fluorine-18-Labeled L-Rhamnose Derivatives. Molecules. 2023 Apr 27;28(9):3773. doi: 10.3390/molecules28093773. PMID: 37175182; PMCID: PMC10180268.

28: Lv J, Yin H, Yu H, Shi H. The added value of ¹⁸F-FDG PET/MRI multimodal imaging in hepatocellular carcinoma for identifying cytokeratin 19 status. Abdom Radiol (NY). 2023 Jul;48(7):2331-2339. doi: 10.1007/s00261-023-03911-3. Epub 2023 Apr 29. PMID: 37119293.

29: Yi M, Liu Y, Chen Q. Xanthogranulomatous pyelonephritis with polycystic kidney disease as a mimic of cystic renal cell carcinoma: a case report. BMC

Urol. 2023 Apr 11;23(1):58. doi: 10.1186/s12894-023-01224-7. PMID: 37038156; PMCID: PMC10088123.

30: John AR, Dwivedi S, Varghese J, Walia GK. Metastatic Hepatocellular Carcinoma Masquerading as an Expansile Osteolytic Lesion in Scapula: A Rare Case of Isolated Appendicular Skeletal Metastatic Involvement of Hepatocellular Carcinoma at Initial Presentation. World J Nucl Med. 2022 Oct 31;22(1):55-58. doi: 10.1055/s-0042-1757288. PMID: 36923980; PMCID: PMC10010855.

31: Sergeeva O, Zhang Y, Gao S, Chan ER, Sergeev M, Iyer R, Sexton S, Avril N, Lu ZR, Lee Z. PET Imaging of Hepatocellular Carcinoma Using ZD2-(⁶⁸Ga-NOTA). J Hepatocell Carcinoma. 2023 Feb 22;10:291-301. doi: 10.2147/JHC.S390939. PMID: 36860804; PMCID: PMC9968869.

32: Koh HD, Choi JW, Kim EK, Park S, Kim MJ, Lee CK. Primary hepatic mucosa-associated lymphoid tissue lymphoma mimicking hepatocellular carcinoma in a patient with chronic hepatitis B: a case report. J Int Med Res. 2023 Feb;51(2):3000605231154399. doi: 10.1177/03000605231154399. PMID: 36788763; PMCID: PMC9932769.

33: Yokoigawa N, Kawaguchi Y. Intraductal Papillary Mucinous Neoplasm of the Pancreas Associated with Polycystic Liver and Kidney Disease. Case Rep Gastroenterol. 2023 Jan 9;17(1):21-25. doi: 10.1159/000528387. PMID: 36742097; PMCID: PMC9893992.

34: Dittrich RP, De Jesus O. Gallium Scan. 2022 Dec 26. In: StatPearls [Internet]. Treasure Island (FL): StatPearls Publishing; 2024 Jan–. PMID: 33620825.

35: Sotoudeh H, Alizadeh M, Shahidi R, Shobeiri P, Love N, Singhal A. Subcortical signal alteration of corticospinal tracts. A radiologic manifestation of ARIA: A case report. Radiol Case Rep. 2022 Nov 6;18(1):275-279. doi: 10.1016/j.radcr.2022.10.023. PMID: 36388611; PMCID: PMC9647162.

36: Pan Y, Lu L, Liu H, Chen D, Han N, Yao R, Wang X, Gao X, Yu J, Chen L, Zhou F, Hao G, Lu Y, Li M, He G, Kang F, Li Z, Tang Y, Zhang J, Wei L, Nie Y. Case report: Long response to PD-1 blockade after failure of trastuzumab plus chemotherapy in advanced Epstein-Barr virus-associated gastric cancer. Front Immunol. 2022 Oct 24;13:1003859. doi: 10.3389/fimmu.2022.1003859. PMID: 36353623; PMCID: PMC9639782.

37: Andre L, Clark MD, Accardo SI. Nuclear Medicine Musculoskeletal Assessment, Protocols, and Interpretation. 2022 Oct 10. In: StatPearls [Internet]. Treasure Island (FL): StatPearls Publishing; 2024 Jan–. PMID: 35015399.

38: Rinaldi V, Crisi PE, Vignoli M, Pierini A, Terragni R, Cabibbo E, Boari A, Finotello R. The Role of Fine Needle Aspiration of Liver and Spleen in the Staging of Low-Grade Canine Cutaneous Mast Cell Tumor. Vet Sci. 2022 Sep 1;9(9):473. doi: 10.3390/vetsci9090473. PMID: 36136689; PMCID: PMC9506313.

39: Ara T, Endo T, Goto H, Kasahara K, Hasegawa Y, Yokoyama S, Shiratori S, Nakagawa M, Kuwahara K, Takakuwa E, Hashino S, Teshima T. Antiretroviral therapy achieved metabolic complete remission of hepatic AIDS related Epstein-Barr virus-associated smooth muscle tumor. Antivir Ther. 2022 Oct;27(5):13596535221126828. doi: 10.1177/13596535221126828. PMID: 36112852.

40: Xian J, Huang H, Huang G, Zhou R, Yang M, Qiu Y, Bi L, Su Z, Xiao F, Shan H, Jin H. A Positron Emission Tomography Tracer Targeting the S2 Subunit of SARS-CoV-2 in Extrapulmonary Infections. Mol Pharm. 2022 Nov 7;19(11):4264-4274. doi: 10.1021/acs.molpharmaceut.2c00584. Epub 2022 Sep 6. PMID: 36067000.

41: Moszczyńska E, Wydra A, Zasada K, Baszyńska-Wilk M, Majak D, Śliwińska A, Grajkowska W. Long-term Survival in a Child with Malignant Insulinoma After Liver Transplantation. J Clin Res Pediatr Endocrinol. 2024 Mar 11;16(1):106-110. doi: 10.4274/jcrpe.galenos.2022.2022-3-5. Epub 2022 Sep 1. PMID: 36047503; PMCID: PMC10938515.

42: Zhang X, Wang N, Wei W, Li Y. Epstein-Barr virus infection-associated cholangiocarcinoma: a report of one case and the review of literature. Virol J. 2022 Aug 9;19(1):133. doi: 10.1186/s12985-022-01862-7. PMID: 35945590; PMCID: PMC9361612.

43: Chen D, Zhu Y, Chen Y, Zhu D, Liu Z, Li T, Liu Y, Zhao K, Su X, Li L. Clinical features and ¹⁸F-FDG PET/CT for distinguishing of malignant lymphoma from inflammatory lymphadenopathy in HIV-infected patients. BMC Infect Dis. 2022 Jul 27;22(1):646. doi: 10.1186/s12879-022-07640-8. PMID: 35896979; PMCID: PMC9327211.

44: Li M, Duan Y, Cheng Z. An Unusual Cause of Intractable Hiccup and Intermittent Low Fever. Gastroenterology. 2022 Dec;163(6):e13-e15. doi: 10.1053/j.gastro.2022.07.011. Epub 2022 Jul 15. PMID: 35850195.

45: Wen XM, Sun J, Liang XX, Xu LL, Zeng XY. [The 497th case: fever, anterior hypopituitarism, lymphadenopathy]. Zhonghua Nei Ke Za Zhi. 2022 Jul 1;61(7):836-839. Chinese. doi: 10.3760/cma.j.cn112138-20210910-00629. PMID: 35764573.

46: Popelier B, Vanheste R, Cuypers S, Heggermont W. An unexpected cause of a swollen pacemaker pocket: a case report. Eur Heart J Case Rep. 2022 May 24;6(6):ytac211. doi: 10.1093/ehjcr/ytac211. PMID: 35685031; PMCID: PMC9174550.

47: Huang Z, Wang H, Ji Z. Giant Polycystic Papillary Renal Cell Carcinoma: A Case Report and Literature Review. Front Oncol. 2022 May 12;12:876217. doi: 10.3389/fonc.2022.876217. PMID: 35646650; PMCID: PMC9134105.

48: Wei A, Lu X, Ma H, Lian H, Yang X, Zhang L, Wang D, Chen S, Zhang Q, Li Z, Zhang R, Yang J, Wang T. ¹⁸F-FDG PET/CT for Identifying the Potential Primary Diseases and Predicting Prognosis of Secondary Hemophagocytic Lymphohistiocytosis in Children. Contrast Media Mol Imaging. 2022 Apr 16;2022:4849081. doi: 10.1155/2022/4849081. PMID: 35510179; PMCID: PMC9034951.

49: Ronsin C, Chaba A, Suchanek O, Coindre JP, Kerleau C, Garandeau C, Houzet A, Cantarovich D, Dantal J, Blancho G, Giral M, Couvrat-Desvergnes G, Ville S. Incidence, Risk Factors and Outcomes of Kidney and Liver Cyst Infection in Kidney Transplant Recipient With ADPKD. Kidney Int Rep. 2022 Feb 3;7(4):867-875. doi: 10.1016/j.ekir.2022.01.1062. PMID: 35497795; PMCID: PMC9039903.

50: Gurusamy VM, Chandramouli SH, Usman M, Divakar SR, Hammoud RW, Al-Hammadi N. Delineating and sparing functional nephrons for radiotherapy in the case of lymphoma with polycystic kidney disease. Cancer Treat Res Commun. 2022;31:100566. doi: 10.1016/j.ctarc.2022.100566. Epub 2022 Apr 21. PMID: 35487053.

51: Wang HJ, Zhou CJ. Occult colon cancer with sepsis as the primary manifestation identified by bone marrow puncture: A case report. World J Clin Cases. 2022 Mar 26;10(9):2969-2975. doi: 10.12998/wjcc.v10.i9.2969. PMID: 35434087; PMCID: PMC8968824.

52: Liu Y, Tong G, Wen Z. Focal Xanthogranulomatous Pyelonephritis on FDG PET/CT. Clin Nucl Med. 2022 Sep 1;47(9):e611-e612. doi: 10.1097/RLU.0000000000004181. Epub 2022 Apr 6. PMID: 35384887.

53: Doroudinia A, Karam MB, Ghadimi N, Yousefi F. Steatotic Hepatitis Presenting as a Huge Hypermetabolic Liver Mass. Clin Nucl Med. 2022 May 1;47(5):e399-e400. doi: 10.1097/RLU.0000000000004098. PMID: 35175943.

54: Lu X, Wei A, Yang X, Liu J, Li S, Kan Y, Wang W, Wang T, Zhang R, Yang J. The Role of Pre-therapeutic ¹⁸F-FDG PET/CT in Pediatric Hemophagocytic Lymphohistiocytosis With Epstein-Barr Virus Infection. Front Med (Lausanne). 2022 Jan 21;8:836438. doi: 10.3389/fmed.2021.836438. PMID: 35127776; PMCID: PMC8813965.

55: Louarn N, Galicier L, Bertinchamp R, Lussato D, Montravers F, Oksenhendler É, Merlet P, Gérard L, Vercellino L. First Extensive Analysis of ¹⁸F-Labeled Fluorodeoxyglucose Positron Emission Tomography-Computed

Tomography in a Large Cohort of Patients With HIV-Associated Hodgkin Lymphoma: Baseline Total Metabolic Tumor Volume Affects Prognosis. J Clin Oncol. 2022 Apr 20;40(12):1346-1355. doi: 10.1200/JCO.21.01228. Epub 2022 Jan 24. PMID: 35073166.

56: Zhuo QF, Liu MQ, Li Z, Liu WS, Shi YH, Xu WY, Ji SR, Xu XW, Yu XJ. [Effect of laparoscopic surgery for pancreatic cancer after neoadjuvant chemotherapy]. Zhonghua Wai Ke Za Zhi. 2022 Feb 1;60(2):134-139. Chinese. doi: 10.3760/cma.j.cn112139-20210823-00389. PMID: 35012272.

57: Li C, Yang X, Lu X, Wang W, Yang J. FDG PET/CT Demonstrated Epstein-Barr Virus-Associated Leiomyosarcoma in a Pediatric Patient With Wiskott-Aldrich Syndrome. Clin Nucl Med. 2022 Feb 1;47(2):190-191. doi: 10.1097/RLU.0000000000004020. PMID: 35006111.

58: Weidenbaum C. Case report: gastric plasmacytoma resistant to radiation therapy. Postgrad Med. 2022 Jan;134(1):122-123. doi: 10.1080/00325481.2021.2008726. Epub 2021 Nov 29. PMID: 34813388.

59: Mitchell R, Kaur A, Munoh Kenne F, Khan A, Zafar W. Spontaneous Regression of Metastatic Lesions of Adenocarcinoma of the Gastro-Esophageal Junction. Cureus. 2021 Oct 14;13(10):e18784. doi: 10.7759/cureus.18784. PMID: 34796071; PMCID: PMC8590531.

60: Abady SM, M Ghanem K, Ghanem NB, Embaby AM. Molecular cloning, heterologous expression, and in silico sequence analysis of Enterobacter GH19 class I chitinase (chiRAM gene). Mol Biol Rep. 2022 Feb;49(2):951-969. doi: 10.1007/s11033-021-06914-9. Epub 2021 Nov 13. PMID: 34773550.

61: López-Mora DA, Fernández A, Duch J, Carrio I. Follow-up ¹⁸F-FDG PET/CT in an oncological asymptomatic COVID-19 patient. Rev Esp Med Nucl Imagen Mol (Engl Ed). 2021 Nov-Dec;40(6):374-375. doi: 10.1016/j.remnie.2021.03.005. Epub 2021 Mar 6. PMID: 34752372; PMCID: PMC7936538.

62: Kaźmierczak O, Kozaczka A, Kolonko A, Kajor M, Pająk J, Chudek J. Advanced Leiomyosarcoma of the Retroperitoneal Space in a Kidney Transplant Recipient with a History of Peritoneal Dialysis: A Case Report. Am J Case Rep. 2021 Oct 25;22:e933267. doi: 10.12659/AJCR.933267. PMID: 34695070; PMCID: PMC8557855.

63: Ronsin C, Bailly C, Le Turnier P, Ville S. Value of FDG-PET/CT in monitoring cyst infections in patients with autosomal dominant polycystic renal disease. Clin Kidney J. 2021 Apr 19;14(10):2273-2275. doi: 10.1093/ckj/sfab077. PMID: 34603707; PMCID: PMC8483683.

64: Suzuki S, Kurokawa R, Tsuruga T, Mori-Uchino M, Nishida H, Kato T, Abe H,

Ushiku T, Amemiya S, Katayama A, Abe O. CT, MRI, and FDG-PET imaging findings of low-grade extrauterine endometrial stromal sarcoma arising from the mesentery: A case report. Radiol Case Rep. 2021 Jul 22;16(9):2774-2779. doi: 10.1016/j.radcr.2021.06.063. PMID: 34367393; PMCID: PMC8326572.

65: Wang LP, Yu YP, Mei JG, Song P, An ZM, Zhou XG. [Clinical and Pathological Characteristics of Aggressive Natural Killer Cell Leukemia Patients]. Zhongguo Shi Yan Xue Ye Xue Za Zhi. 2021 Aug;29(4):1093-1100. Chinese. doi: 10.19746/j.cnki.issn.1009-2137.2021.04.012. PMID: 34362487.

66: Sakaguchi T, Hashimoto D, Satoi S, Yamamoto T, Yamaki S, Sekimoto M. Hepatic actinomycosis after total pancreatectomy: A case report. Int J Surg Case Rep. 2021 Aug;85:106212. doi: 10.1016/j.ijscr.2021.106212. Epub 2021 Jul 20. PMID: 34330068; PMCID: PMC8335620.

67: Ye Y, Yang N, Zhou J, Qian G, Chu J. Case Report: Metagenomic Next-Generation Sequencing in Diagnosis of Disseminated Tuberculosis of an Immunocompetent Patient. Front Med (Lausanne). 2021 Jul 12;8:687984. doi: 10.3389/fmed.2021.687984. PMID: 34322503; PMCID: PMC8310911.

68: Ojeda Gómez A, Íñigo Chaves AM, Barragán Martínez J, Madero Velázquez L, Picó Sala MD, García Soria A, Sáez Fuster J, García Sepulcre MF. What are the odds? Duodenum adenocarcinoma as a primary metachronic neoplasia. Rev Esp Enferm Dig. 2021 Nov;113(11):793-794. doi: 10.17235/reed.2021.8200/2021. PMID: 34320812.

69: Liu Q, Yang Y, Fan X, Xin X, Pan Q, Zhang Y, Liu B, Wei J. Heterogeneity response to afatinib in gastric cancer patient with uncommon epidermal growth factor receptor (EGFR) mutations: a case report. Ann Transl Med. 2021 May;9(9):814. doi: 10.21037/atm-20-7312. PMID: 34268427; PMCID: PMC8246221.

70: Fang G, Cheng NC, Huang LL, Xie WP, Hu CM, Chen W. The first report of co-existence of pulmonary tuberculosis and lung malignancy in a kidney transplant recipient: a case report and literature review. BMC Infect Dis. 2021 Jul 1;21(1):629. doi: 10.1186/s12879-021-06350-x. PMID: 34210287; PMCID: PMC8252204.

71: Chiu TF, Yu TM, Chiu CW, Lee BK, Lan TH, Li CY, Lin MC, Kao CH. Increased risk of pulmonary and extrapulmonary tuberculosis infection in patients with polycystic kidney disease: a nationwide population-based study with propensity score-matching analysis. J Transl Med. 2021 Jun 9;19(1):253. doi: 10.1186/s12967-021-02921-3. PMID: 34107991; PMCID: PMC8191203.

72: Boldizsár S, Rottek J, Schneider T, Varga F, Szaleczky E. Hodgkin-lymphomához társult eltűnőepeút-szindróma [Hodgkin's lymphoma-related vanishing bile duct syndrome]. Orv Hetil. 2021 May 30;162(22):884-888. Hungarian. doi:

10.1556/650.2021.32093. PMID: 34052798.

73: Wu J, Ma HZ, Apaer S, Anweier N, Zeng Q, Fulati X, Li T, Zhao JM, Wen H, Tuxun T. Impact of Albendazole on Cytokine and Chemokine Response Profiles in <i>Echinococcus multilocularis</i>-Inoculated Mice. Biomed Res Int. 2021 May 7;2021:6628814. doi: 10.1155/2021/6628814. PMID: 34041299; PMCID: PMC8121589.

74: Sharma P. Fever of Unknown Origin: ¹⁸F-Fluorodeoxyglucose Positron Emission Tomography-Computed Tomography Showing Renal Cyst Infection in Autosomal Dominant Polycystic Kidney Disease. Indian J Nucl Med. 2021 Jan-Mar;36(1):43-45. doi: 10.4103/ijnm.IJNM_139_20. Epub 2021 Mar 4. PMID: 34040295; PMCID: PMC8130681.

75: Wang J, Zhao B, Song T, Sun J. Lung cancer combined with diffuse peritoneal and mesenteric amyloidosis detected on 18F-FDG PET/CT: A case report. Medicine (Baltimore). 2021 May 28;100(21):e25961. doi: 10.1097/MD.0000000000025961. PMID: 34032706; PMCID: PMC8154378.

76: Yasuda T, Ishikawa T, Hirose R, Doi T, Inoue K, Dohi O, Yoshida N, Kamada K, Uchiyama K, Takagi T, Konishi H, Inamori O, Morinaga Y, Konishi E, Naito Y, Itoh Y. Aggressive advanced gastric cancer in a patient with autosomal dominant polycystic kidney disease. Clin J Gastroenterol. 2021 Aug;14(4):1014-1019. doi: 10.1007/s12328-021-01407-5. Epub 2021 May 24. PMID: 34028786.

77: Liu Y, Tao Y, Wang F, Huang Z. Desmoplastic Small Round Cell Tumor of the Kidney With Mainly Pulmonary Symptoms by ¹⁸F-FDG PET/CT. Urology. 2021 Aug;154:e15-e16. doi: 10.1016/j.urology.2021.04.026. Epub 2021 May 5. PMID: 33964278.

78: Maruno M, Imai K, Nakao Y, Kitano Y, Kaida T, Mima K, Hayashi H, Yamashita YI, Mikami Y, Baba H. Multiple hepatic inflammatory pseudotumors with elevated alpha-fetoprotein and alpha-fetoprotein lectin 3 fraction with various PET accumulations: a case report. Surg Case Rep. 2021 Apr 28;7(1):107. doi: 10.1186/s40792-021-01188-6. PMID: 33913027; PMCID: PMC8081814.

79: Hermel M, Jones D, Olson C, Sherman M, Srivastava A. Vasoactive intestinal peptide producing pheochromocytoma and intracardiac thrombosis. Rare Tumors. 2021 Apr 9;13:20363613211007792. doi: 10.1177/20363613211007792. PMID: 33889374; PMCID: PMC8040591.

80: Chang SC, Tsai CY, Liu KH, Wang SY, Hsu JT, Yeh TS, Yeh CN. Everolimus Related Fulminant Hepatitis in Pancreatic Neuroendocrine Tumor With Liver Metastases: A Case Report and Literature Review. Front Endocrinol (Lausanne). 2021 Apr 1;12:639967. doi: 10.3389/fendo.2021.639967. PMID: 33868173; PMCID: PMC8047461.

81: Issa Z, Ciccarelli O, Devresse A, Kanaan N, Larranaga Lapique E, De Greef J, Lhommel R, Kerschen A, Komuta M, Delire B, Dahlqvist G. Sequential Liver-Kidney Transplantation for Recurrent Liver Cysts Infection in a Patient With Autosomal Dominant Polycystic Kidney Disease: A Case Report. Transplant Proc. 2021 May;53(4):1322-1326. doi: 10.1016/j.transproceed.2021.02.018. Epub 2021 Apr 12. PMID: 33858690.

82: Valamparampil JJ, Palaniappan K, Vij M, Shanmugam N, Ramachandran P, Ramachandran B, Rela M. Recurrent Intestinal Perforation After Cessation of Immunosuppression in Posttransplant Lymphoproliferative Disease in Pediatric Liver Transplant Recipient. J Gastrointest Cancer. 2021 Sep;52(3):1165-1168. doi: 10.1007/s12029-021-00627-9. Epub 2021 Mar 22. PMID: 33751328.

83: Klionsky DJ, Abdel-Aziz AK, Abdelfatah S, Abdellatif M, Abdoli A, Abel S, Abeliovich H, Abildgaard MH, Abudu YP, Acevedo-Arozena A, Adamopoulos IE, Adeli K, Adolph TE, Adornetto A, Aflaki E, Agam G, Agarwal A, Aggarwal BB, Agnello M, Agostinis P, Agrewala JN, Agrotis A, Aguilar PV, Ahmad ST, Ahmed ZM, Ahumada-Castro U, Aits S, Aizawa S, Akkoc Y, Akoumianaki T, Akpinar HA, Al-Abd AM, Al-Akra L, Al-Gharaibeh A, Alaoui-Jamali MA, Alberti S, Alcocer-Gómez E, Alessandri C, Ali M, Alim Al-Bari MA, Aliwaini S, Alizadeh J, Almacellas E, Almasan A, Alonso A, Alonso GD, Altan-Bonnet N, Altieri DC, Álvarez ÉMC, Alves S, Alves da Costa C, Alzaharna MM, Amadio M, Amantini C, Amaral C, Ambrosio S, Amer AO, Ammanathan V, An Z, Andersen SU, Andrabi SA, Andrade-Silva M, Andres AM, Angelini S, Ann D, Anozie UC, Ansari MY, Antas P, Antebi A, Antón Z, Anwar T, Apetoh L, Apostolova N, Araki T, Araki Y, Arasaki K, Araújo WL, Araya J, Arden C, Arévalo MA, Arguelles S, Arias E, Arikkath J, Arimoto H, Ariosa AR, Armstrong-James D, Arnauné-Pelloquin L, Aroca A, Arroyo DS, Arsov I, Artero R, Asaro DML, Aschner M, Ashrafizadeh M, Ashur-Fabian O, Atanasov AG, Au AK, Auberger P, Auner HW, Aurelian L, Autelli R, Avagliano L, Ávalos Y, Aveic S, Aveleira CA, Avin-Wittenberg T, Aydin Y, Ayton S, Ayyadevara S, Azzopardi M, Baba M, Backer JM, Backues SK, Bae DH, Bae ON, Bae SH, Baehrecke EH, Baek A, Baek SH, Baek SH, Bagetta G, Bagniewska-Zadworna A, Bai H, Bai J, Bai X, Bai Y, Bairagi N, Baksi S, Balbi T, Baldari CT, Balduini W, Ballabio A, Ballester M, Balazadeh S, Balzan R, Bandopadhyay R, Banerjee S, Banerjee S, Bánréti Á, Bao Y, Baptista MS, Baracca A, Barbati C, Bargiela A, Barilà D, Barlow PG, Barmada SJ, Barreiro E, Barreto GE, Bartek J, Bartel B, Bartolome A, Barve GR, Basagoudanavar SH, Bassham DC, Bast RC Jr, Basu A, Batoko H, Batten I, Baulieu EE, Baumgarner BL, Bayry J, Beale R, Beau I, Beaumatin F, Bechara LRG, Beck GR Jr, Beers MF, Begun J, Behrends C, Behrens GMN, Bei R, Bejarano E, Bel S, Behl C, Belaid A, Belgareh-Touzé N, Bellarosa C, Belleudi F, Belló Pérez M, Bello-Morales R, Beltran JSO, Beltran S, Benbrook DM, Bendorius M, Benitez BA, Benito-Cuesta I, Bensalem J, Berchtold MW, Berezowska S, Bergamaschi D, Bergami M, Bergmann A, Berliocchi L, Berlioz-Torrent C, Bernard A, Berthoux L, Besirli CG, Besteiro S, Betin VM, Beyaert R, Bezbradica JS, Bhaskar K, Bhatia-Kissova I,

Bhattacharya R, Bhattacharya S, Bhattacharyya S, Bhuiyan MS, Bhutia SK, Bi L, Bi X, Biden TJ, Bijian K, Billes VA, Binart N, Bincoletto C, Birgisdottir AB, Bjorkoy G, Blanco G, Blas-Garcia A, Blasiak J, Blomgran R, Blomgren K, Blum JS, Boada-Romero E, Boban M, Boesze-Battaglia K, Boeuf P, Boland B, Bomont P, Bonaldo P, Bonam SR, Bonfili L, Bonifacino JS, Boone BA, Bootman MD, Bordi M, Borner C, Bornhauser BC, Borthakur G, Bosch J, Bose S, Botana LM, Botas J, Boulanger CM, Boulton ME, Bourdenx M, Bourgeois B, Bourke NM, Bousquet G, Boya P, Bozhkov PV, Bozi LHM, Bozkurt TO, Brackney DE, Brandts CH, Braun RJ, Braus GH, Bravo-Sagua R, Bravo-San Pedro JM, Brest P, Bringer MA, Briones-Herrera A, Broaddus VC, Brodersen P, Brodsky JL, Brody SL, Bronson PG, Bronstein JM, Brown CN, Brown RE, Brum PC, Brumell JH, Brunetti-Pierri N, Bruno D, Bryson-Richardson RJ, Bucci C, Buchrieser C, Bueno M, Buitrago-Molina LE, Buraschi S, Buch S, Buchan JR, Buckingham EM, Budak H, Budini M, Bultynck G, Burada F, Burgoyne JR, Burón MI, Bustos V, Büttner S, Butturini E, Byrd A, Cabas I, Cabrera-Benitez S, Cadwell K, Cai J, Cai L, Cai Q, Cairó M, Calbet JA, Caldwell GA, Caldwell KA, Call JA, Calvani R, Calvo AC, Calvo-Rubio Barrera M, Camara NO, Camonis JH, Camougrand N, Campanella M, Campbell EM, Campbell-Valois FX, Campello S, Campesi I, Campos JC, Camuzard O, Cancino J, Candido de Almeida D, Canesi L, Caniggia I, Canonico B, Cantí C, Cao B, Caraglia M, Caramés B, Carchman EH, Cardenal-Muñoz E, Cardenas C, Cardenas L, Cardoso SM, Carew JS, Carle GF, Carleton G, Carloni S, Carmona-Gutierrez D, Carneiro LA, Carnevali O, Carosi JM, Carra S, Carrier A, Carrier L, Carroll B, Carter AB, Carvalho AN, Casanova M, Casas C, Casas J, Cassioli C, Castillo EF, Castillo K, Castillo-Lluva S, Castoldi F, Castori M, Castro AF, Castro-Caldas M, Castro-Hernandez J, Castro-Obregon S, Catz SD, Cavadas C, Cavaliere F, Cavallini G, Cavinato M, Cayuela ML, Cebollada Rica P, Cecarini V, Cecconi F, Cechowska-Pasko M, Cenci S, Ceperuelo-Mallafré V, Cerqueira JJ, Cerutti JM, Cervia D, Cetintas VB, Cetrullo S, Chae HJ, Chagin AS, Chai CY, Chakrabarti G, Chakrabarti O, Chakraborty T, Chakraborty T, Chami M, Chamilos G, Chan DW, Chan EYW, Chan ED, Chan HYE, Chan HH, Chan H, Chan MTV, Chan YS, Chandra PK, Chang CP, Chang C, Chang HC, Chang K, Chao J, Chapman T, Charlet-Berguerand N, Chatterjee S, Chaube SK, Chaudhary A, Chauhan S, Chaum E, Checler F, Cheetham ME, Chen CS, Chen GC, Chen JF, Chen LL, Chen L, Chen L, Chen M, Chen MK, Chen N, Chen Q, Chen RH, Chen S, Chen W, Chen W, Chen XM, Chen XW, Chen X, Chen Y, Chen YG, Chen Y, Chen Y, Chen YJ, Chen YQ, Chen ZS, Chen Z, Chen ZH, Chen ZJ, Chen Z, Cheng H, Cheng J, Cheng SY, Cheng W, Cheng X, Cheng XT, Cheng Y, Cheng Z, Chen Z, Cheong H, Cheong JK, Chernyak BV, Cherry S, Cheung CFR, Cheung CHA, Cheung KH, Chevet E, Chi RJ, Chiang AKS, Chiaradonna F, Chiarelli R, Chiariello M, Chica N, Chiocca S, Chiong M, Chiou SH, Chiramel AI, Chiurchiù V, Cho DH, Choe SK, Choi AMK, Choi ME, Choudhury KR, Chow NS, Chu CT, Chua JP, Chua JJE, Chung H, Chung KP, Chung S, Chung SH, Chung YL, Cianfanelli V, Ciechomska IA, Cifuentes M, Cinque L, Cirak S, Cirone M, Clague MJ, Clarke R, Clementi E, Coccia EM, Codogno P, Cohen E, Cohen MM, Colasanti T, Colasuonno F, Colbert RA, Colell A, Čolić M, Coll NS, Collins MO, Colombo MI, Colón-Ramos DA, Combaret L, Comincini S, Cominetti MR, Consiglio A, Conte A, Conti F, Contu VR, Cookson MR, Coombs KM, Coppens I, Corasaniti MT, Corkery DP, Cordes N, Cortese

K, Costa MDC, Costantino S, Costelli P, Coto-Montes A, Crack PJ, Crespo JL, Criollo A, Crippa V, Cristofani R, Csizmadia T, Cuadrado A, Cui B, Cui J, Cui Y, Cui Y, Culetto E, Cumino AC, Cybulsky AV, Czaja MJ, Czuczwar SJ, D'Adamo S, D'Amelio M, D'Arcangelo D, D'Lugos AC, D'Orazi G, da Silva JA, Dafsari HS, Dagda RK, Dagdas Y, Daglia M, Dai X, Dai Y, Dai Y, Dal Col J, Dalhaimer P, Dalla Valle L, Dallenga T, Dalmasso G, Damme M, Dando I, Dantuma NP, Darling AL, Das H, Dasarathy S, Dasari SK, Dash S, Daumke O, Dauphinee AN, Davies JS, Dávila VA, Davis RJ, Davis T, Dayalan Naidu S, De Amicis F, De Bosscher K, De Felice F, De Franceschi L, De Leonibus C, de Mattos Barbosa MG, De Meyer GRY, De Milito A, De Nunzio C, De Palma C, De Santi M, De Virgilio C, De Zio D, Debnath J, DeBosch BJ, Decuypere JP, Deehan MA, Deflorian G, DeGregori J, Dehay B, Del Rio G, Delaney JR, Delbridge LMD, Delorme-Axford E, Delpino MV, Demarchi F, Dembitz V, Demers ND, Deng H, Deng Z, Dengjel J, Dent P, Denton D, DePamphilis ML, Der CJ, Deretic V, Descoteaux A, Devis L, Devkota S, Devuyst O, Dewson G, Dharmasivam M, Dhiman R, di Bernardo D, Di Cristina M, Di Domenico F, Di Fazio P, Di Fonzo A, Di Guardo G, Di Guglielmo GM, Di Leo L, Di Malta C, Di Nardo A, Di Rienzo M, Di Sano F, Diallinas G, Diao J, Diaz-Araya G, Díaz-Laviada I, Dickinson JM, Diederich M, Dieudé M, Dikic I, Ding S, Ding WX, Dini L, Dinić J, Dinic M, Dinkova-Kostova AT, Dionne MS, Distler JHW, Diwan A, Dixon IMC, Djavaheri-Mergny M, Dobrinski I, Dobrovinskaya O, Dobrowolski R, Dobson RCJ, Đokić J, Dokmeci Emre S, Donadelli M, Dong B, Dong X, Dong Z, Dorn Ii GW, Dotsch V, Dou H, Dou J, Dowaidar M, Dridi S, Drucker L, Du A, Du C, Du G, Du HN, Du LL, du Toit A, Duan SB, Duan X, Duarte SP, Dubrovska A, Dunlop EA, Dupont N, Durán RV, Dwarakanath BS, Dyshlovoy SA, Ebrahimi-Fakhari D, Eckhart L, Edelstein CL, Efferth T, Eftekharpour E, Eichinger L, Eid N, Eisenberg T, Eissa NT, Eissa S, Ejarque M, El Andaloussi A, El-Hage N, El-Naggar S, Eleuteri AM, El-Shafey ES, Elgendy M, Eliopoulos AG, Elizalde MM, Elks PM, Elsasser HP, Elsherbiny ES, Emerling BM, Emre NCT, Eng CH, Engedal N, Engelbrecht AM, Engelsen AST, Enserink JM, Escalante R, Esclatine A, Escobar-Henriques M, Eskelinen EL, Espert L, Eusebio MO, Fabrias G, Fabrizi C, Facchiano A, Facchiano F, Fadeel B, Fader C, Faesen AC, Fairlie WD, Falcó A, Falkenburger BH, Fan D, Fan J, Fan Y, Fang EF, Fang Y, Fang Y, Fanto M, Farfel-Becker T, Faure M, Fazeli G, Fedele AO, Feldman AM, Feng D, Feng J, Feng L, Feng Y, Feng Y, Feng W, Fenz Araujo T, Ferguson TA, Fernández ÁF, Fernandez-Checa JC, Fernández-Veledo S, Fernie AR, Ferrante AW Jr, Ferraresi A, Ferrari MF, Ferreira JCB, Ferro-Novick S, Figueras A, Filadi R, Filigheddu N, Filippi-Chiela E, Filomeni G, Fimia GM, Fineschi V, Finetti F, Finkbeiner S, Fisher EA, Fisher PB, Flamigni F, Fliesler SJ, Flo TH, Florance I, Florey O, Florio T, Fodor E, Follo C, Fon EA, Forlino A, Fornai F, Fortini P, Fracassi A, Fraldi A, Franco B, Franco R, Franconi F, Frankel LB, Friedman SL, Fröhlich LF, Frühbeck G, Fuentes JM, Fujiki Y, Fujita N, Fujiwara Y, Fukuda M, Fulda S, Furic L, Furuya N, Fusco C, Gack MU, Gaffke L, Galadari S, Galasso A, Galindo MF, Gallolu Kankanamalage S, Galluzzi L, Galy V, Gammoh N, Gan B, Ganley IG, Gao F, Gao H, Gao M, Gao P, Gao SJ, Gao W, Gao X, Garcera A, Garcia MN, Garcia VE, García-Del Portillo F, Garcia-Escudero V, Garcia-Garcia A, Garcia-Macia M, García-Moreno D, Garcia-Ruiz C, García-Sanz P, Garg AD, Gargini R, Garofalo T,

Garry RF, Gassen NC, Gatica D, Ge L, Ge W, Geiss-Friedlander R, Gelfi C, Genschik P, Gentle IE, Gerbino V, Gerhardt C, Germain K, Germain M, Gewirtz DA, Ghasemipour Afshar E, Ghavami S, Ghigo A, Ghosh M, Giamas G, Giampietri C, Giatromanolaki A, Gibson GE, Gibson SB, Ginet V, Giniger E, Giorgi C, Girao H, Girardin SE, Giridharan M, Giuliano S, Giulivi C, Giuriato S, Giustiniani J, Gluschko A, Goder V, Goginashvili A, Golab J, Goldstone DC, Golebiewska A, Gomes LR, Gomez R, Gómez-Sánchez R, Gomez-Puerto MC, Gomez-Sintes R, Gong Q, Goni FM, González-Gallego J, Gonzalez-Hernandez T, Gonzalez-Polo RA, Gonzalez-Reyes JA, González-Rodríguez P, Goping IS, Gorbatyuk MS, Gorbunov NV, Görgülü K, Gorojod RM, Gorski SM, Goruppi S, Gotor C, Gottlieb RA, Gozes I, Gozuacik D, Graef M, Gräler MH, Granatiero V, Grasso D, Gray JP, Green DR, Greenhough A, Gregory SL, Griffin EF, Grinstaff MW, Gros F, Grose C, Gross AS, Gruber F, Grumati P, Grune T, Gu X, Guan JL, Guardia CM, Guda K, Guerra F, Guerri C, Guha P, Guillén C, Gujar S, Gukovskaya A, Gukovsky I, Gunst J, Günther A, Guntur AR, Guo C, Guo C, Guo H, Guo LW, Guo M, Gupta P, Gupta SK, Gupta S, Gupta VB, Gupta V, Gustafsson AB, Gutterman DD, H B R, Haapasalo A, Haber JE, Hać A, Hadano S, Hafrén AJ, Haidar M, Hall BS, Halldén G, Hamacher-Brady A, Hamann A, Hamasaki M, Han W, Hansen M, Hanson PI, Hao Z, Harada M, Harhaji-Trajkovic L, Hariharan N, Haroon N, Harris J, Hasegawa T, Hasima Nagoor N, Haspel JA, Haucke V, Hawkins WD, Hay BA, Haynes CM, Hayrabedyan SB, Hays TS, He C, He Q, He RR, He YW, He YY, Heakal Y, Heberle AM, Hejtmancik JF, Helgason GV, Henkel V, Herb M, Hergovich A, Herman-Antosiewicz A, Hernández A, Hernandez C, Hernandez-Diaz S, Hernandez-Gea V, Herpin A, Herreros J, Hervás JH, Hesselson D, Hetz C, Heussler VT, Higuchi Y, Hilfiker S, Hill JA, Hlavacek WS, Ho EA, Ho IHT, Ho PW, Ho SL, Ho WY, Hobbs GA, Hochstrasser M, Hoet PHM, Hofius D, Hofman P, Höhn A, Holmberg CI, Hombrebueno JR, Yi-Ren Hong CH, Hooper LV, Hoppe T, Horos R, Hoshida Y, Hsin IL, Hsu HY, Hu B, Hu D, Hu LF, Hu MC, Hu R, Hu W, Hu YC, Hu ZW, Hua F, Hua J, Hua Y, Huan C, Huang C, Huang C, Huang C, Huang C, Huang H, Huang K, Huang MLH, Huang R, Huang S, Huang T, Huang X, Huang YJ, Huber TB, Hubert V, Hubner CA, Hughes SM, Hughes WE, Humbert M, Hummer G, Hurley JH, Hussain S, Hussain S, Hussey PJ, Hutabarat M, Hwang HY, Hwang S, Ieni A, Ikeda F, Imagawa Y, Imai Y, Imbriano C, Imoto M, Inman DM, Inoki K, Iovanna J, Iozzo RV, Ippolito G, Irazoqui JE, Iribarren P, Ishaq M, Ishikawa M, Ishimwe N, Isidoro C, Ismail N, Issazadeh-Navikas S, Itakura E, Ito D, Ivankovic D, Ivanova S, Iyer AKV, Izquierdo JM, Izumi M, Jäättelä M, Jabir MS, Jackson WT, Jacobo-Herrera N, Jacomin AC, Jacquin E, Jadiya P, Jaeschke H, Jagannath C, Jakobi AJ, Jakobsson J, Janji B, Jansen-Dürr P, Jansson PJ, Jantsch J, Januszewski S, Jassey A, Jean S, Jeltsch-David H, Jendelova P, Jenny A, Jensen TE, Jessen N, Jewell JL, Ji J, Jia L, Jia R, Jiang L, Jiang Q, Jiang R, Jiang T, Jiang X, Jiang Y, Jimenez-Sanchez M, Jin EJ, Jin F, Jin H, Jin L, Jin L, Jin M, Jin S, Jo EK, Joffre C, Johansen T, Johnson GVW, Johnston SA, Jokitalo E, Jolly MK, Joosten LAB, Jordan J, Joseph B, Ju D, Ju JS, Ju J, Juárez E, Judith D, Juhász G, Jun Y, Jung CH, Jung SC, Jung YK, Jungbluth H, Jungverdorben J, Just S, Kaarniranta K, Kaasik A, Kabuta T, Kaganovich D, Kahana A, Kain R, Kajimura S, Kalamvoki M, Kalia M, Kalinowski DS, Kaludercic N, Kalvari I, Kaminska J, Kaminskyy VO, Kanamori H, Kanasaki K, Kang C, Kang R,

Kang SS, Kaniyappan S, Kanki T, Kanneganti TD, Kanthasamy AG, Kanthasamy A, Kantorow M, Kapuy O, Karamouzis MV, Karim MR, Karmakar P, Katare RG, Kato M, Kaufmann SHE, Kauppinen A, Kaushal GP, Kaushik S, Kawasaki K, Kazan K, Ke PY, Keating DJ, Keber U, Kehrl JH, Keller KE, Keller CW, Kemper JK, Kenific CM, Kepp O, Kermorgant S, Kern A, Ketteler R, Keulers TG, Khalfin B, Khalil H, Khambu B, Khan SY, Khandelwal VKM, Khandia R, Kho W, Khobrekar NV, Khuansuwan S, Khundadze M, Killackey SA, Kim D, Kim DR, Kim DH, Kim DE, Kim EY, Kim EK, Kim HR, Kim HS, Hyung-Ryong Kim, Kim JH, Kim JK, Kim JH, Kim J, Kim JH, Kim KI, Kim PK, Kim SJ, Kimball SR, Kimchi A, Kimmelman AC, Kimura T, King MA, Kinghorn KJ, Kinsey CG, Kirkin V, Kirshenbaum LA, Kiselev SL, Kishi S, Kitamoto K, Kitaoka Y, Kitazato K, Kitsis RN, Kittler JT, Kjaerulff O, Klein PS, Klopstock T, Klucken J, Knævelsrud H, Knorr RL, Ko BCB, Ko F, Ko JL, Kobayashi H, Kobayashi S, Koch I, Koch JC, Koenig U, Kögel D, Koh YH, Koike M, Kohlwein SD, Kocaturk NM, Komatsu M, König J, Kono T, Kopp BT, Korcsmaros T, Korkmaz G, Korolchuk VI, Korsnes MS, Koskela A, Kota J, Kotake Y, Kotler ML, Kou Y, Koukourakis MI, Koustas E, Kovacs AL, Kovács T, Koya D, Kozako T, Kraft C, Krainc D, Krämer H, Krasnodembskaya AD, Kretz-Remy C, Kroemer G, Ktistakis NT, Kuchitsu K, Kuenen S, Kuerschner L, Kukar T, Kumar A, Kumar A, Kumar D, Kumar D, Kumar S, Kume S, Kumsta C, Kundu CN, Kundu M, Kunnumakkara AB, Kurgan L, Kutateladze TG, Kutlu O, Kwak S, Kwon HJ, Kwon TK, Kwon YT, Kyrmizi I, La Spada A, Labonté P, Ladoire S, Laface I, Lafont F, Lagace DC, Lahiri V, Lai Z, Laird AS, Lakkaraju A, Lamark T, Lan SH, Landajuela A, Lane DJR, Lane JD, Lang CH, Lange C, Langel Ü, Langer R, Lapaquette P, Laporte J, LaRusso NF, Lastres-Becker I, Lau WCY, Laurie GW, Lavandero S, Law BYK, Law HK, Layfield R, Le W, Le Stunff H, Leary AY, Lebrun JJ, Leck LYW, Leduc-Gaudet JP, Lee C, Lee CP, Lee DH, Lee EB, Lee EF, Lee GM, Lee HJ, Lee HK, Lee JM, Lee JS, Lee JA, Lee JY, Lee JH, Lee M, Lee MG, Lee MJ, Lee MS, Lee SY, Lee SJ, Lee SY, Lee SB, Lee WH, Lee YR, Lee YH, Lee Y, Lefebvre C, Legouis R, Lei YL, Lei Y, Leikin S, Leitinger G, Lemus L, Leng S, Lenoir O, Lenz G, Lenz HJ, Lenzi P, León Y, Leopoldino AM, Leschczyk C, Leskelä S, Letellier E, Leung CT, Leung PS, Leventhal JS, Levine B, Lewis PA, Ley K, Li B, Li DQ, Li J, Li J, Li J, Li K, Li L, Li M, Li M, Li M, Li M, Li M, Li PL, Li MQ, Li Q, Li S, Li T, Li W, Li W, Li X, Li YP, Li Y, Li Z, Li Z, Li Z, Lian J, Liang C, Liang Q, Liang W, Liang Y, Liang Y, Liao G, Liao L, Liao M, Liao YF, Librizzi M, Lie PPY, Lilly MA, Lim HJ, Lima TRR, Limana F, Lin C, Lin CW, Lin DS, Lin FC, Lin JD, Lin KM, Lin KH, Lin LT, Lin PH, Lin Q, Lin S, Lin SJ, Lin W, Lin X, Lin YX, Lin YS, Linden R, Lindner P, Ling SC, Lingor P, Linnemann AK, Liou YC, Lipinski MM, Lipovšek S, Lira VA, Lisiak N, Liton PB, Liu C, Liu CH, Liu CF, Liu CH, Liu F, Liu H, Liu HS, Liu HF, Liu H, Liu J, Liu J, Liu J, Liu L, Liu L, Liu M, Liu Q, Liu W, Liu W, Liu XH, Liu X, Liu X, Liu X, Liu X, Liu Y, Liu Y, Liu Y, Liu Y, Liu Y, Livingston JA, Lizard G, Lizcano JM, Ljubojevic-Holzer S, LLeonart ME, Llobet-Navàs D, Llorente A, Lo CH, Lobato-Márquez D, Long Q, Long YC, Loos B, Loos JA, López MG, López-Doménech G, López-Guerrero JA, López-Jiménez AT, López-Pérez Ó, López-Valero I, Lorenowicz MJ, Lorente M, Lorincz P, Lossi L, Lotersztajn S, Lovat PE, Lovell JF, Lovy A, Lőw P, Lu G, Lu H, Lu JH, Lu JJ, Lu M, Lu S, Luciani A, Lucocq JM, Ludovico P, Luftig MA, Luhr M, Luis-Ravelo D, Lum

JJ, Luna-Dulcey L, Lund AH, Lund VK, Lünemann JD, Lüningschrör P, Luo H, Luo R, Luo S, Luo Z, Luparello C, Lüscher B, Luu L, Lyakhovich A, Lyamzaev KG, Lystad AH, Lytvynchuk L, Ma AC, Ma C, Ma M, Ma NF, Ma QH, Ma X, Ma Y, Ma Z, MacDougald OA, Macian F, MacIntosh GC, MacKeigan JP, Macleod KF, Maday S, Madeo F, Madesh M, Madl T, Madrigal-Matute J, Maeda A, Maejima Y, Magarinos M, Mahavadi P, Maiani E, Maiese K, Maiti P, Maiuri MC, Majello B, Major MB, Makareeva E, Malik F, Mallilankaraman K, Malorni W, Maloyan A, Mammadova N, Man GCW, Manai F, Mancias JD, Mandelkow EM, Mandell MA, Manfredi AA, Manjili MH, Manjithaya R, Manque P, Manshian BB, Manzano R, Manzoni C, Mao K, Marchese C, Marchetti S, Marconi AM, Marcucci F, Mardente S, Mareninova OA, Margeta M, Mari M, Marinelli S, Marinelli O, Mariño G, Mariotto S, Marshall RS, Marten MR, Martens S, Martin APJ, Martin KR, Martin S, Martin S, Martín-Segura A, Martín-Acebes MA, Martin-Burriel I, Martin-Rincon M, Martin-Sanz P, Martina JA, Martinet W, Martinez A, Martinez A, Martinez J, Martinez Velazquez M, Martinez-Lopez N, Martinez-Vicente M, Martins DO, Martins JO, Martins WK, Martins-Marques T, Marzetti E, Masaldan S, Masclaux-Daubresse C, Mashek DG, Massa V, Massieu L, Masson GR, Masuelli L, Masyuk AI, Masyuk TV, Matarrese P, Matheu A, Matoba S, Matsuzaki S, Mattar P, Matte A, Mattoscio D, Mauriz JL, Mauthe M, Mauvezin C, Maverakis E, Maycotte P, Mayer J, Mazzoccoli G, Mazzoni C, Mazzulli JR, McCarty N, McDonald C, McGill MR, McKenna SL, McLaughlin B, McLoughlin F, McNiven MA, McWilliams TG, Mechta-Grigoriou F, Medeiros TC, Medina DL, Megeney LA, Megyeri K, Mehrpour M, Mehta JL, Meijer AJ, Meijer AH, Mejlvang J, Meléndez A, Melk A, Memisoglu G, Mendes AF, Meng D, Meng F, Meng T, Menna-Barreto R, Menon MB, Mercer C, Mercier AE, Mergny JL, Merighi A, Merkley SD, Merla G, Meske V, Mestre AC, Metur SP, Meyer C, Meyer H, Mi W, Mialet-Perez J, Miao J, Micale L, Miki Y, Milan E, Milczarek M, Miller DL, Miller SI, Miller S, Millward SW, Milosevic I, Minina EA, Mirzaei H, Mirzaei HR, Mirzaei M, Mishra A, Mishra N, Mishra PK, Misirkic Marjanovic M, Misasi R, Misra A, Misso G, Mitchell C, Mitou G, Miura T, Miyamoto S, Miyazaki M, Miyazaki M, Miyazaki T, Miyazawa K, Mizushima N, Mogensen TH, Mograbi B, Mohammadinejad R, Mohamud Y, Mohanty A, Mohapatra S, Möhlmann T, Mohmmed A, Moles A, Moley KH, Molinari M, Mollace V, Møller AB, Mollereau B, Mollinedo F, Montagna C, Monteiro MJ, Montella A, Montes LR, Montico B, Mony VK, Monzio Compagnoni G, Moore MN, Moosavi MA, Mora AL, Mora M, Morales-Alamo D, Moratalla R, Moreira PI, Morelli E, Moreno S, Moreno-Blas D, Moresi V, Morga B, Morgan AH, Morin F, Morishita H, Moritz OL, Moriyama M, Moriyasu Y, Morleo M, Morselli E, Moruno-Manchon JF, Moscat J, Mostowy S, Motori E, Moura AF, Moustaid-Moussa N, Mrakovcic M, Muciño-Hernández G, Mukherjee A, Mukhopadhyay S, Mulcahy Levy JM, Mulero V, Muller S, Münch C, Munjal A, Munoz-Canoves P, Muñoz-Galdeano T, Münz C, Murakawa T, Muratori C, Murphy BM, Murphy JP, Murthy A, Myöhänen TT, Mysorekar IU, Mytych J, Nabavi SM, Nabissi M, Nagy P, Nah J, Nahimana A, Nakagawa I, Nakamura K, Nakatogawa H, Nandi SS, Nanjundan M, Nanni M, Napolitano G, Nardacci R, Narita M, Nassif M, Nathan I, Natsumeda M, Naude RJ, Naumann C, Naveiras O, Navid F, Nawrocki ST, Nazarko TY, Nazio F, Negoita F, Neill T, Neisch AL, Neri LM, Netea MG, Neubert P, Neufeld TP, Neumann D, Neutzner A, Newton PT, Ney PA, Nezis IP, Ng CCW, Ng TB, Nguyen HTT, Nguyen LT, Ni HM, Ní

Cheallaigh C, Ni Z, Nicolao MC, Nicoli F, Nieto-Diaz M, Nilsson P, Ning S, Niranjan R, Nishimune H, Niso-Santano M, Nixon RA, Nobili A, Nobrega C, Noda T, Nogueira-Recalde U, Nolan TM, Nombela I, Novak I, Novoa B, Nozawa T, Nukina N, Nussbaum-Krammer C, Nylandsted J, O'Donovan TR, O'Leary SM, O'Rourke EJ, O'Sullivan MP, O'Sullivan TE, Oddo S, Oehme I, Ogawa M, Ogier-Denis E, Ogmundsdottir MH, Ogretmen B, Oh GT, Oh SH, Oh YJ, Ohama T, Ohashi Y, Ohmuraya M, Oikonomou V, Ojha R, Okamoto K, Okazawa H, Oku M, Oliván S, Oliveira JMA, Ollmann M, Olzmann JA, Omari S, Omary MB, Önal G, Ondrej M, Ong SB, Ong SG, Onnis A, Orellana JA, Orellana-Muñoz S, Ortega-Villaizan MDM, Ortiz-Gonzalez XR, Ortona E, Osiewacz HD, Osman AK, Osta R, Otegui MS, Otsu K, Ott C, Ottobrini L, Ou JJ, Outeiro TF, Oynebraten I, Ozturk M, Pagès G, Pahari S, Pajares M, Pajvani UB, Pal R, Paladino S, Pallet N, Palmieri M, Palmisano G, Palumbo C, Pampaloni F, Pan L, Pan Q, Pan W, Pan X, Panasyuk G, Pandey R, Pandey UB, Pandya V, Paneni F, Pang SY, Panzarini E, Papademetrio DL, Papaleo E, Papinski D, Papp D, Park EC, Park HT, Park JM, Park JI, Park JT, Park J, Park SC, Park SY, Parola AH, Parys JB, Pasquier A, Pasquier B, Passos JF, Pastore N, Patel HH, Patschan D, Pattingre S, Pedraza-Alva G, Pedraza-Chaverri J, Pedrozo Z, Pei G, Pei J, Peled-Zehavi H, Pellegrini JM, Pelletier J, Peñalva MA, Peng D, Peng Y, Penna F, Pennuto M, Pentimalli F, Pereira CM, Pereira GJS, Pereira LC, Pereira de Almeida L, Perera ND, Pérez-Lara Á, Perez-Oliva AB, Pérez-Pérez ME, Periyasamy P, Perl A, Perrotta C, Perrotta I, Pestell RG, Petersen M, Petrache I, Petrovski G, Pfirrmann T, Pfister AS, Philips JA, Pi H, Picca A, Pickrell AM, Picot S, Pierantoni GM, Pierdominici M, Pierre P, Pierrefite-Carle V, Pierzynowska K, Pietrocola F, Pietruczuk M, Pignata C, Pimentel-Muiños FX, Pinar M, Pinheiro RO, Pinkas-Kramarski R, Pinton P, Pircs K, Piya S, Pizzo P, Plantinga TS, Platta HW, Plaza-Zabala A, Plomann M, Plotnikov EY, Plun-Favreau H, Pluta R, Pocock R, Pöggeler S, Pohl C, Poirot M, Poletti A, Ponpuak M, Popelka H, Popova B, Porta H, Porte Alcon S, Portilla-Fernandez E, Post M, Potts MB, Poulton J, Powers T, Prahlad V, Prajsnar TK, Praticò D, Prencipe R, Priault M, Proikas-Cezanne T, Promponas VJ, Proud CG, Puertollano R, Puglielli L, Pulinilkunnil T, Puri D, Puri R, Puyal J, Qi X, Qi Y, Qian W, Qiang L, Qiu Y, Quadrilatero J, Quarleri J, Raben N, Rabinowich H, Ragona D, Ragusa MJ, Rahimi N, Rahmati M, Raia V, Raimundo N, Rajasekaran NS, Ramachandra Rao S, Rami A, Ramírez-Pardo I, Ramsden DB, Randow F, Rangarajan PN, Ranieri D, Rao H, Rao L, Rao R, Rathore S, Ratnayaka JA, Ratovitski EA, Ravanan P, Ravegnini G, Ray SK, Razani B, Rebecca V, Reggiori F, Régnier-Vigouroux A, Reichert AS, Reigada D, Reiling JH, Rein T, Reipert S, Rekha RS, Ren H, Ren J, Ren W, Renault T, Renga G, Reue K, Rewitz K, Ribeiro de Andrade Ramos B, Riazuddin SA, Ribeiro-Rodrigues TM, Ricci JE, Ricci R, Riccio V, Richardson DR, Rikihisa Y, Risbud MV, Risueño RM, Ritis K, Rizza S, Rizzuto R, Roberts HC, Roberts LD, Robinson KJ, Roccheri MC, Rocchi S, Rodney GG, Rodrigues T, Rodrigues Silva VR, Rodriguez A, Rodriguez-Barrueco R, Rodriguez-Henche N, Rodriguez-Rocha H, Roelofs J, Rogers RS, Rogov VV, Rojo AI, Rolka K, Romanello V, Romani L, Romano A, Romano PS, Romeo-Guitart D, Romero LC, Romero M, Roney JC, Rongo C, Roperto S, Rosenfeldt MT, Rosenstiel P, Rosenwald AG, Roth KA, Roth L, Roth S, Rouschop KMA, Roussel BD, Roux S, Rovere-Querini P,

Roy A, Rozieres A, Ruano D, Rubinsztein DC, Rubtsova MP, Ruckdeschel K, Ruckenstuhl C, Rudolf E, Rudolf R, Ruggieri A, Ruparelia AA, Rusmini P, Russell RR, Russo GL, Russo M, Russo R, Ryabaya OO, Ryan KM, Ryu KY, Sabater-Arcis M, Sachdev U, Sacher M, Sachse C, Sadhu A, Sadoshima J, Safren N, Saftig P, Sagona AP, Sahay G, Sahebkar A, Sahin M, Sahin O, Sahni S, Saito N, Saito S, Saito T, Sakai R, Sakai Y, Sakamaki JI, Saksela K, Salazar G, Salazar-Degracia A, Salekdeh GH, Saluja AK, Sampaio-Marques B, Sanchez MC, Sanchez-Alcazar JA, Sanchez-Vera V, Sancho-Shimizu V, Sanderson JT, Sandri M, Santaguida S, Santambrogio L, Santana MM, Santoni G, Sanz A, Sanz P, Saran S, Sardiello M, Sargeant TJ, Sarin A, Sarkar C, Sarkar S, Sarrias MR, Sarkar S, Sarmah DT, Sarparanta J, Sathyanarayan A, Sathyanarayanan R, Scaglione KM, Scatozza F, Schaefer L, Schafer ZT, Schaible UE, Schapira AHV, Scharl M, Schatzl HM, Schein CH, Scheper W, Scheuring D, Schiaffino MV, Schiappacassi M, Schindl R, Schlattner U, Schmidt O, Schmitt R, Schmidt SD, Schmitz I, Schmukler E, Schneider A, Schneider BE, Schober R, Schoijet AC, Schott MB, Schramm M, Schröder B, Schuh K, Schüller C, Schulze RJ, Schürmanns L, Schwamborn JC, Schwarten M, Scialo F, Sciarretta S, Scott MJ, Scotto KW, Scovassi AI, Scrima A, Scrivo A, Sebastian D, Sebti S, Sedej S, Segatori L, Segev N, Seglen PO, Seiliez I, Seki E, Selleck SB, Sellke FW, Selsby JT, Sendtner M, Senturk S, Seranova E, Sergi C, Serra-Moreno R, Sesaki H, Settembre C, Setty SRG, Sgarbi G, Sha O, Shacka JJ, Shah JA, Shang D, Shao C, Shao F, Sharbati S, Sharkey LM, Sharma D, Sharma G, Sharma K, Sharma P, Sharma S, Shen HM, Shen H, Shen J, Shen M, Shen W, Shen Z, Sheng R, Sheng Z, Sheng ZH, Shi J, Shi X, Shi YH, Shiba-Fukushima K, Shieh JJ, Shimada Y, Shimizu S, Shimozawa M, Shintani T, Shoemaker CJ, Shojaei S, Shoji I, Shravage BV, Shridhar V, Shu CW, Shu HB, Shui K, Shukla AK, Shutt TE, Sica V, Siddiqui A, Sierra A, Sierra-Torre V, Signorelli S, Sil P, Silva BJA, Silva JD, Silva-Pavez E, Silvente-Poirot S, Simmonds RE, Simon AK, Simon HU, Simons M, Singh A, Singh LP, Singh R, Singh SV, Singh SK, Singh SB, Singh S, Singh SP, Sinha D, Sinha RA, Sinha S, Sirko A, Sirohi K, Sivridis EL, Skendros P, Skirycz A, Slaninová I, Smaili SS, Smertenko A, Smith MD, Soenen SJ, Sohn EJ, Sok SPM, Solaini G, Soldati T, Soleimanpour SA, Soler RM, Solovchenko A, Somarelli JA, Sonawane A, Song F, Song HK, Song JX, Song K, Song Z, Soria LR, Sorice M, Soukas AA, Soukup SF, Sousa D, Sousa N, Spagnuolo PA, Spector SA, Srinivas Bharath MM, St Clair D, Stagni V, Staiano L, Stalnecker CA, Stankov MV, Stathopulos PB, Stefan K, Stefan SM, Stefanis L, Steffan JS, Steinkasserer A, Stenmark H, Sterneckert J, Stevens C, Stoka V, Storch S, Stork B, Strappazzon F, Strohecker AM, Stupack DG, Su H, Su LY, Su L, Suarez-Fontes AM, Subauste CS, Subbian S, Subirada PV, Sudhandiran G, Sue CM, Sui X, Summers C, Sun G, Sun J, Sun K, Sun MX, Sun Q, Sun Y, Sun Z, Sunahara KKS, Sundberg E, Susztak K, Sutovsky P, Suzuki H, Sweeney G, Symons JD, Sze SCW, Szewczyk NJ, Tabęcka-Łonczynska A, Tabolacci C, Tacke F, Taegtmeyer H, Tafani M, Tagaya M, Tai H, Tait SWG, Takahashi Y, Takats S, Talwar P, Tam C, Tam SY, Tampellini D, Tamura A, Tan CT, Tan EK, Tan YQ, Tanaka M, Tanaka M, Tang D, Tang J, Tang TS, Tanida I, Tao Z, Taouis M, Tatenhorst L, Tavernarakis N, Taylor A, Taylor GA, Taylor JM, Tchetina E, Tee AR, Tegeder I, Teis D, Teixeira N, Teixeira-Clerc F,

Tekirdag KA, Tencomnao T, Tenreiro S, Tepikin AV, Testillano PS, Tettamanti G, Tharaux PL, Thedieck K, Thekkinghat AA, Thellung S, Thinwa JW, Thirumalaikumar VP, Thomas SM, Thomes PG, Thorburn A, Thukral L, Thum T, Thumm M, Tian L, Tichy A, Till A, Timmerman V, Titorenko VI, Todi SV, Todorova K, Toivonen JM, Tomaipitinca L, Tomar D, Tomas-Zapico C, Tomić S, Tong BC, Tong C, Tong X, Tooze SA, Torgersen ML, Torii S, Torres-López L, Torriglia A, Towers CG, Towns R, Toyokuni S, Trajkovic V, Tramontano D, Tran QG, Travassos LH, Trelford CB, Tremel S, Trougakos IP, Tsao BP, Tschan MP, Tse HF, Tse TF, Tsugawa H, Tsvetkov AS, Tumbarello DA, Tumtas Y, Tuñón MJ, Turcotte S, Turk B, Turk V, Turner BJ, Tuxworth RI, Tyler JK, Tyutereva EV, Uchiyama Y, Ugun-Klusek A, Uhlig HH, Ułamek-Kozioł M, Ulasov IV, Umekawa M, Ungermann C, Unno R, Urbe S, Uribe-Carretero E, Üstün S, Uversky VN, Vaccari T, Vaccaro MI, Vahsen BF, Vakifahmetoglu-Norberg H, Valdor R, Valente MJ, Valko A, Vallee RB, Valverde AM, Van den Berghe G, van der Veen S, Van Kaer L, van Loosdregt J, van Wijk SJL, Vandenberghe W, Vanhorebeek I, Vannier-Santos MA, Vannini N, Vanrell MC, Vantaggiato C, Varano G, Varela-Nieto I, Varga M, Vasconcelos MH, Vats S, Vavvas DG, Vega-Naredo I, Vega-Rubin-de-Celis S, Velasco G, Velázquez AP, Vellai T, Vellenga E, Velotti F, Verdier M, Verginis P, Vergne I, Verkade P, Verma M, Verstreken P, Vervliet T, Vervoorts J, Vessoni AT, Victor VM, Vidal M, Vidoni C, Vieira OV, Vierstra RD, Viganó S, Vihinen H, Vijayan V, Vila M, Vilar M, Villalba JM, Villalobo A, Villarejo-Zori B, Villarroya F, Villarroya J, Vincent O, Vindis C, Viret C, Viscomi MT, Visnjic D, Vitale I, Vocadlo DJ, Voitsekhovskaja OV, Volonté C, Volta M, Vomero M, Von Haefen C, Vooijs MA, Voos W, Vucicevic L, Wade-Martins R, Waguri S, Waite KA, Wakatsuki S, Walker DW, Walker MJ, Walker SA, Walter J, Wandosell FG, Wang B, Wang CY, Wang C, Wang C, Wang C, Wang CY, Wang D, Wang F, Wang F, Wang F, Wang G, Wang H, Wang H, Wang H, Wang HG, Wang J, Wang J, Wang J, Wang J, Wang K, Wang L, Wang L, Wang MH, Wang M, Wang N, Wang P, Wang P, Wang P, Wang P, Wang QJ, Wang Q, Wang QK, Wang QA, Wang WT, Wang W, Wang X, Wang X, Wang Y, Wang Y, Wang Y, Wang YY, Wang Y, Wang Y, Wang Y, Wang Y, Wang Z, Wang Z, Wang Z, Warnes G, Warnsmann V, Watada H, Watanabe E, Watchon M, Wawrzyńska A, Weaver TE, Wegrzyn G, Wehman AM, Wei H, Wei L, Wei T, Wei Y, Weiergräber OH, Weihl CC, Weindl G, Weiskirchen R, Wells A, Wen RH, Wen X, Werner A, Weykopf B, Wheatley SP, Whitton JL, Whitworth AJ, Wiktorska K, Wildenberg ME, Wileman T, Wilkinson S, Willbold D, Williams B, Williams RSB, Williams RL, Williamson PR, Wilson RA, Winner B, Winsor NJ, Witkin SS, Wodrich H, Woehlbier U, Wollert T, Wong E, Wong JH, Wong RW, Wong VKW, Wong WW, Wu AG, Wu C, Wu J, Wu J, Wu KK, Wu M, Wu SY, Wu S, Wu SY, Wu S, Wu WKK, Wu X, Wu X, Wu YW, Wu Y, Xavier RJ, Xia H, Xia L, Xia Z, Xiang G, Xiang J, Xiang M, Xiang W, Xiao B, Xiao G, Xiao H, Xiao HT, Xiao J, Xiao L, Xiao S, Xiao Y, Xie B, Xie CM, Xie M, Xie Y, Xie Z, Xie Z, Xilouri M, Xu C, Xu E, Xu H, Xu J, Xu J, Xu L, Xu WW, Xu X, Xue Y, Yakhine-Diop SMS, Yamaguchi M, Yamaguchi O, Yamamoto A, Yamashina S, Yan S, Yan SJ, Yan Z, Yanagi Y, Yang C, Yang DS, Yang H, Yang HT, Yang H, Yang JM, Yang J, Yang J, Yang L, Yang L, Yang M, Yang PM, Yang Q, Yang S, Yang S, Yang SF, Yang W, Yang WY, Yang X, Yang X, Yang Y, Yang Y, Yao H, Yao S, Yao X, Yao YG, Yao YM, Yasui T, Yazdankhah M, Yen PM, Yi C, Yin XM, Yin Y,

Yin Z, Yin Z, Ying M, Ying Z, Yip CK, Yiu SPT, Yoo YH, Yoshida K, Yoshii SR, Yoshimori T, Yousefi B, Yu B, Yu H, Yu J, Yu J, Yu L, Yu ML, Yu SW, Yu VC, Yu WH, Yu Z, Yu Z, Yuan J, Yuan LQ, Yuan S, Yuan SF, Yuan Y, Yuan Z, Yue J, Yue Z, Yun J, Yung RL, Zacks DN, Zaffagnini G, Zambelli VO, Zanella I, Zang QS, Zanivan S, Zappavigna S, Zaragoza P, Zarbalis KS, Zarebkohan A, Zarrouk A, Zeitlin SO, Zeng J, Zeng JD, Žerovnik E, Zhan L, Zhang B, Zhang DD, Zhang H, Zhang H, Zhang H, Zhang H, Zhang H, Zhang H, Zhang H, Zhang HL, Zhang J, Zhang J, Zhang JP, Zhang KYB, Zhang LW, Zhang L, Zhang L, Zhang L, Zhang L, Zhang M, Zhang P, Zhang S, Zhang W, Zhang X, Zhang XW, Zhang X, Zhang X, Zhang X, Zhang X, Zhang XD, Zhang Y, Zhang Y, Zhang Y, Zhang YD, Zhang Y, Zhang YY, Zhang Y, Zhang Z, Zhang Z, Zhang Z, Zhang Z, Zhang Z, Zhang Z, Zhao H, Zhao L, Zhao S, Zhao T, Zhao XF, Zhao Y, Zhao Y, Zhao Y, Zhao Y, Zheng G, Zheng K, Zheng L, Zheng S, Zheng XL, Zheng Y, Zheng ZG, Zhivotovsky B, Zhong Q, Zhou A, Zhou B, Zhou C, Zhou G, Zhou H, Zhou H, Zhou H, Zhou J, Zhou J, Zhou J, Zhou J, Zhou K, Zhou R, Zhou XJ, Zhou Y, Zhou Y, Zhou Y, Zhou ZY, Zhou Z, Zhu B, Zhu C, Zhu GQ, Zhu H, Zhu H, Zhu H, Zhu WG, Zhu Y, Zhu Y, Zhuang H, Zhuang X, Zientara-Rytter K, Zimmermann CM, Ziviani E, Zoladek T, Zong WX, Zorov DB, Zorzano A, Zou W, Zou Z, Zou Z, Zuryn S, Zwerschke W, Brand-Saberi B, Dong XC, Kenchappa CS, Li Z, Lin Y, Oshima S, Rong Y, Sluimer JC, Stallings CL, Tong CK. Guidelines for the use and interpretation of assays for monitoring autophagy (4th edition)¹. Autophagy. 2021 Jan;17(1):1-382. doi: 10.1080/15548627.2020.1797280. Epub 2021 Feb 8. PMID: 33634751; PMCID: PMC7996087.

84: Devarbhavi P, Telang L, Vastrad B, Tengli A, Vastrad C, Kotturshetti I. Identification of key pathways and genes in polycystic ovary syndrome via integrated bioinformatics analysis and prediction of small therapeutic molecules. Reprod Biol Endocrinol. 2021 Feb 23;19(1):31. doi: 10.1186/s12958-021-00706-3. PMID: 33622336; PMCID: PMC7901211.

85: Fırıncıoğluları A, Akıncı Özyürek B, Erdoğan Y, İncekara F, Yılmaz E, Özaydın HE. Lymphoepithelioma-like carcinoma of the lung: a rare case report and review of the literature. Tuberk Toraks. 2020 Dec;68(4):453-457. English. doi: 10.5578/tt.69322. PMID: 33448744.

86: Tajima H, Makino I, Gabata R, Okazaki M, Ohbatake Y, Shimbashi H, Nakanuma S, Saitoh H, Shimada M, Yamaguchi T, Okamoto K, Moriyama H, Kinoshita J, Nakamura K, Miyashita T, Ninomiya I, Fushida S, Ikeda H, Ohta T. A phase I study of preoperative (neoadjuvant) chemotherapy with gemcitabine plus <i>nab</i>-paclitaxel for resectable pancreatic cancer. Mol Clin Oncol. 2021 Feb;14(2):26. doi: 10.3892/mco.2020.2188. Epub 2020 Dec 14. PMID: 33414907; PMCID: PMC7783717.

87: Qian XH, Zhou DK, Wang WL. Surgical treatment of Epstein-Barr virus-associated lymphoepithelioma-like carcinoma occurring in both the posterior mediastinum and liver: Case report. Medicine (Baltimore). 2020 Dec

24;99(52):e23610. doi: 10.1097/MD.0000000000023610. PMID: 33350736; PMCID: PMC7769299.

88: Wee J, Sobhi S, De Boer B, Xu D. Liver rheumatoid nodules imitating liver malignancy: a rare occurrence. BMJ Case Rep. 2020 Dec 16;13(12):e234366. doi: 10.1136/bcr-2020-234366. PMID: 33328208; PMCID: PMC7745683.

89: Chen D, Chang C, Chen M, Zhang Y, Zhao X, Zhang T, Wang Z, Yan J, Zhu H, Zheng L, Zhao K. Unusual disseminated Talaromyces marneffei infection mimicking lymphoma in a non-immunosuppressed patient in East China: a case report and review of the literature. BMC Infect Dis. 2020 Oct 28;20(1):800. doi: 10.1186/s12879-020-05526-1. PMID: 33115429; PMCID: PMC7594432.

90: Quintero Vega GE, Osorio D, de la Hoz Valle JA, Rodríguez Feria D. Limited Renal Intravascular Lymphoma: A Case Report and Review of the Literature. Case Rep Oncol Med. 2020 Oct 6;2020:7052536. doi: 10.1155/2020/7052536. PMID: 33083073; PMCID: PMC7563074.

91: Mendes FR, Sobral KM, Culler HF, Couto SCF, Pereira J, Rocha V, Martinez GA, Lage LAPC. Acquired hemophagocytic lymphohistiocytosis as initial manifestation of multiple myeloma: A case report and literature review. Medicine (Baltimore). 2020 Sep 25;99(39):e22299. doi: 10.1097/MD.0000000000022299. PMID: 32991435; PMCID: PMC7523830.

92: Feng H, Tu N, Bu L. Malignant Peripheral Nerve Sheath Tumor Arising From Transplanted Kidney Assessed by 18F-FDG PET/CT. Clin Nucl Med. 2020 Nov;45(11):905-907. doi: 10.1097/RLU.0000000000003273. PMID: 32969901.

93: Filippi L, Iannarelli A, Ambrogi C, Bagni O. Atrial Metastasis From Sarcomatoid Renal Cell Carcinoma: Integration Between 18F-FDG PET/CT and Cardiac 3-Dimensional Volume Rendering. Clin Nucl Med. 2020 Dec;45(12):970-972. doi: 10.1097/RLU.0000000000003302. PMID: 32956126.

94: Cosma L, Sollaku S, Frantellizzi V, De Vincentis G. Early ¹⁸ F-FDG PET/CT in COVID-19. J Med Imaging Radiat Oncol. 2020 Oct;64(5):671-673. doi: 10.1111/1754-9485.13099. Epub 2020 Sep 17. PMID: 32945110; PMCID: PMC7537199.

95: Aedma SK, Chidharla A, Kelting S, Kasi A. Oxaliplatin-associated sarcoid-like reaction masquerading as recurrent colon cancer. BMJ Case Rep. 2020 Sep 8;13(9):e229548. doi: 10.1136/bcr-2019-229548. PMID: 32907862; PMCID: PMC7481089.

96: Wakfie-Corieh CG, Blanes García AM, Ferrando-Castagnetto F, Valhondo-Rama R, Ortega Candil A, Rodríguez Rey C, Cabrera Martín MN, García-Esquinas MG, Couto

Caro RM, Pedrera Canal M, Carreras Delgado JL. Assessment of extra-parenchymal lung involvement in asymptomatic cancer patients with COVID-19 pneumonia detected on ¹⁸F-FDG PET-CT studies. Eur J Nucl Med Mol Imaging. 2021 Mar;48(3):768-776. doi: 10.1007/s00259-020-05019-y. Epub 2020 Sep 8. PMID: 32901353; PMCID: PMC7478863.

97: De Jaeghere EA, Laloo F, Lippens L, Van Bockstal M, De Man K, Naert E, Van Dorpe J, Van de Vijver K, Tummers P, Makar A, De Visschere PJL, De Wever O, Amant F, Denys HG, Vandecasteele K. Splenic ¹⁸F-FDG uptake on baseline PET/CT is associated with oncological outcomes and tumor immune state in uterine cervical cancer. Gynecol Oncol. 2020 Nov;159(2):335-343. doi: 10.1016/j.ygyno.2020.08.001. Epub 2020 Aug 25. PMID: 32859399.

98: Guberina N, Gäckler A, Grueneisen J, Wetter A, Witzke O, Herrmann K, Rischpler C, Fendler W, Umutlu L, Sawicki LM, Forsting M, Rohn H. Assessment of Suspected Malignancy or Infection in Immunocompromised Patients After Solid Organ Transplantation by [¹⁸F]FDG PET/CT and [¹⁸F]FDG PET/MRI. Nucl Med Mol Imaging. 2020 Aug;54(4):183-191. doi: 10.1007/s13139-020-00648-5. Epub 2020 Jul 2. PMID: 32831964; PMCID: PMC7429581.

99: Yu TM, Li CY, Chuang YW, Chen CH, Lee BK, Chung MC, Chiu HF, Lin MC, Wu MJ, Kao CH. Risk of severe herpes zoster infection in patients with polycystic kidney disease: A nation-wide cohort study with propensity score matching analysis. Int J Clin Pract. 2021 Apr;75(4):e13675. doi: 10.1111/ijcp.13675. Epub 2020 Dec 12. PMID: 32798268.

100: Kong Y, Zhou H, Feng H, Zhuang J, Wen T, Zhang C, Sun B, Wang J, Guan Y. Elucidating the Relationship Between Diabetes Mellitus and Parkinson's Disease Using ¹⁸F-FP-(+)-DTBZ, a Positron-Emission Tomography Probe for Vesicular Monoamine Transporter 2. Front Neurosci. 2020 Jul 14;14:682. doi: 10.3389/fnins.2020.00682. PMID: 32760240; PMCID: PMC7372188.

101: Suwabe T. Cyst infection in autosomal dominant polycystic kidney disease: our experience at Toranomon Hospital and future issues. Clin Exp Nephrol. 2020 Sep;24(9):748-761. doi: 10.1007/s10157-020-01928-2. Epub 2020 Jul 22. PMID: 32700112; PMCID: PMC7474715.

102: Ho CW, Chen HH, Hsieh MC, Chen CC, Hsu SP, Yip HT, Kao CH. Hashimoto's thyroiditis might increase polycystic ovary syndrome and associated comorbidities risks in Asia. Ann Transl Med. 2020 Jun;8(11):684. doi: 10.21037/atm-19-4763. PMID: 32617304; PMCID: PMC7327368.

103: Neuville MF, Lovinfosse P, Jadoul A, Thys M, Seidel L, Hustinx R, Jouret F. The use of a visual 4-point scoring scale improves the yield of ¹⁸F-FDG PET-CT imaging in the diagnosis of renal and hepatic cyst

infection in patients with autosomal dominant polycystic kidney disease. Eur J Nucl Med Mol Imaging. 2021 Jan;48(1):254-259. doi: 10.1007/s00259-020-04903-x. Epub 2020 Jun 15. PMID: 32556485.

104: Saritas AG, Atar C, Gul MO, Teke Z, Ulku A, Sahin B. Bilateral breast lesions mimicking metastases of hepatocellular carcinoma in a male patient. Ann Ital Chir. 2020 May 11;9:S2239253X2003279X. PMID: 32554904.

105: Frye BC, Rump IC, Uhlmann A, Schubach F, Ihorst G, Grimbacher B, Zissel G, Quernheim JM. Safety and efficacy of abatacept in patients with treatment-resistant SARCoidosis (ABASARC) - protocol for a multi-center, single-arm phase IIa trial. Contemp Clin Trials Commun. 2020 May 29;19:100575. doi: 10.1016/j.conctc.2020.100575. PMID: 32551397; PMCID: PMC7292904.

106: Artigas C, Lemort M, Mestrez F, Gil T, Flamen P. COVID-19 Pneumonia Mimicking Immunotherapy-Induced Pneumonitis on 18F-FDG PET/CT in a Patient Under Treatment With Nivolumab. Clin Nucl Med. 2020 Aug;45(8):e381-e382. doi: 10.1097/RLU.0000000000003152. PMID: 32520508; PMCID: PMC7315837.

107: Xiang Q, Liu Z, Yu Y, Zhang H, Xie Q, Mu G, Zhang J, Cen X, Cui Y. Osteomalacia and renal failure due to Fanconi syndrome caused by long-term low-dose Adefovir Dipivoxil: a case report. BMC Pharmacol Toxicol. 2020 Jun 5;21(1):43. doi: 10.1186/s40360-020-00421-6. PMID: 32503675; PMCID: PMC7275610.

108: Prabhu M, Damle NA, Ray A, Kandasamy D. ¹⁸F-FDG PET/CT in cyst infection in autosomal dominant polycystic kidney disease. Indian J Radiol Imaging. 2020 Jan-Mar;30(1):102-104. doi: 10.4103/ijri.IJRI_469_18. Epub 2020 Mar 30. PMID: 32476761; PMCID: PMC7240894.

109: Tohyama T, Sakamoto K, Tamura K, Nakamura T, Watanabe J, Wakisaka H, Takada Y. Pharyngeal metastasis following living-donor liver transplantation for hepatocellular carcinoma: a case report and literature review. World J Surg Oncol. 2020 May 28;18(1):109. doi: 10.1186/s12957-020-01873-0. PMID: 32466780; PMCID: PMC7257203.

110: Krebs S, Petkovska I, Ho AL, Ulaner GA. Laboratory-Proven Asymptomatic SARS-CoV-2 (COVID-19) Infection on 18F-FDG PET/CT. Clin Nucl Med. 2020 Aug;45(8):654-655. doi: 10.1097/RLU.0000000000003141. PMID: 32433161; PMCID: PMC7268849.

111: Oliveira FR, Mamede M, Bizzi MF, Rocha ALL, Ferreira CN, Gomes KB, Cândido AL, Reis FM. Effects of Short Term Metformin Treatment on Brown Adipose Tissue Activity and Plasma Irisin Levels in Women with Polycystic Ovary Syndrome: A Randomized Controlled Trial. Horm Metab Res. 2020 Oct;52(10):718-723. doi: 10.1055/a-1157-0615. Epub 2020 May 4. PMID: 32365398.

112: Ishibashi K, Saida K, Kimura M, Nishiwaki S, Tsuji H, Umemura M. Case report of API2-MALT1 fusion-positive MALT lymphoma arising from bilateral submandibular glands with no evidence of autoimmune syndromes. Oral Surg Oral Med Oral Pathol Oral Radiol. 2020 Oct;130(4):e294-e300. doi: 10.1016/j.oooo.2019.12.018. Epub 2020 Apr 18. PMID: 32312530.

113: Kang SR, Jo EJ, Nguyen VH, Zhang Y, Yoon HS, Pyo A, Kim DY, Hong Y, Bom HS, Min JJ. Imaging of tumor colonization by <i>Escherichia coli</i> using ¹⁸F-FDS PET. Theranostics. 2020 Apr 1;10(11):4958-4966. doi: 10.7150/thno.42121. PMID: 32308761; PMCID: PMC7163454.

114: Hardy AJ, Stoica I, Kearney DE, O'Riordain DS. Diffuse large B-cell lymphoma of the rectum in a patient with Crohn's disease. BMJ Case Rep. 2020 Apr 16;13(4):e228818. doi: 10.1136/bcr-2018-228818. PMID: 32303524; PMCID: PMC7199099.

115: Chen HH, Chen CC, Hsieh MC, Ho CW, Hsu SP, Yip HT, Kao CH. Graves' disease could increase polycystic ovary syndrome and comorbidities in Taiwan. Curr Med Res Opin. 2020 Jun;36(6):1063-1067. doi: 10.1080/03007995.2020.1756235. Epub 2020 Apr 28. PMID: 32297812.

116: Lazzarotto D, Facchin G, Candoni A, Fanin R. 18F-FDG Positron Emission Tomography (PET) Scan as a Useful Tool to Assess Response to Antimycotic Therapy in Disseminated Invasive Mycosis in Acute Myeloid Leukemia. Mycopathologia. 2020 Jun;185(3):597-598. doi: 10.1007/s11046-020-00441-0. Epub 2020 Apr 7. PMID: 32266558.

117: Ho CW, Chen HH, Hsieh MC, Chen CC, Hsu SP, Yip HT, Kao CH. Increased Risk of Polycystic Ovary Syndrome and It's Comorbidities in Women with Autoimmune Thyroid Disease. Int J Environ Res Public Health. 2020 Apr 2;17(7):2422. doi: 10.3390/ijerph17072422. PMID: 32252386; PMCID: PMC7177418.

118: Manenti G, Censi M, Pizzicannella G, Pucci N, Pitocchi F, Calcagni A, Amico A, Collura A, Ryan CP. Vertebral hydatid cyst infection. A case report. Radiol Case Rep. 2020 Mar 3;15(5):523-527. doi: 10.1016/j.radcr.2020.01.029. PMID: 32153692; PMCID: PMC7057157.

119: Pérez-Díaz V, Pérez-Escudero A, Sanz-Ballesteros S, Sánchez-García L, Hernández-García E, Oviedo-Gómez V, Sobrino-Pérez A. Clinical relevance of marginal factors on ultrafiltration in peritoneal dialysis. Perit Dial Int. 2021 Jan;41(1):86-95. doi: 10.1177/0896860820904556. Epub 2020 Feb 12. PMID: 32048915.

120: Matsubayashi H, Ishiwatari H, Imai K, Kishida Y, Ito S, Hotta K, Yabuuchi

Y, Yoshida M, Kakushima N, Takizawa K, Kawata N, Ono H. Steroid Therapy and Steroid Response in Autoimmune Pancreatitis. Int J Mol Sci. 2019 Dec 30;21(1):257. doi: 10.3390/ijms21010257. PMID: 31905944; PMCID: PMC6981453.

121: Chesdachai S, Udompap P, Li F, Lake JR, Kc M. Pulmonary Mycobacterium Spindle Cell Pseudotumor in Patient With Liver Transplant. Am J Med Sci. 2020 Jan;359(1):42-50. doi: 10.1016/j.amjms.2019.10.016. Epub 2019 Nov 5. PMID: 31902440.

122: Maccora I, Marrani E, Ricci S, Azzari C, Simonini G, Cimaz R, Giani T. Common variable immunodeficiency presenting as sarcoidosis in a 9-year-old child. Int J Rheum Dis. 2020 Mar;23(3):448-453. doi: 10.1111/1756-185X.13775. Epub 2019 Dec 19. PMID: 31858744.

123: Ribeiro L, Rajendran S, Stenson K, Loftus I. Rare case of a proximal descending thoracic aorta mycotic aneurysm following intravesical BCG injections for the treatment of bladder cancer. BMJ Case Rep. 2019 Dec 15;12(12):e231595. doi: 10.1136/bcr-2019-231595. PMID: 31843771; PMCID: PMC6936417.

124: Lin SY, Yang YC, Chang CY, Lin CC, Hsu WH, Ju SW, Hsu CY, Kao CH. Risk of Polycystic Ovary Syndrome in Women Exposed to Fine Air Pollutants and Acidic Gases: A Nationwide Cohort Analysis. Int J Environ Res Public Health. 2019 Nov 30;16(23):4816. doi: 10.3390/ijerph16234816. PMID: 31801197; PMCID: PMC6926786.

125: Zhou X, Li P, Gu X, Zheng F, Zhao J, Zhao L. A case report of right atrial epithelioid hemangioendothelioma with multiple pulmonary metastases. Clin Respir J. 2020 Feb;14(2):173-178. doi: 10.1111/crj.13121. Epub 2019 Dec 17. PMID: 31794151.

126: Baumgartner K, Kübler J, Bitzer M, Bösmüller H, Horger M. Caroli-Syndrom [Caroli's syndrome]. Rofo. 2020 Feb;192(2):119-122. German. doi: 10.1055/a-1024-4526. Epub 2019 Nov 28. PMID: 31779027.

127: Wong FCK, Chan AZ, Wong WS, Kwan AHW, Law TSM, Chung JPW, Kwok JSS, Chan AOK. Hyperandrogenism, Elevated 17-Hydroxyprogesterone and Its Urinary Metabolites in a Young Woman with Ovarian Steroid Cell Tumor, Not Otherwise Specified: Case Report and Review of the Literature. Case Rep Endocrinol. 2019 Oct 27;2019:9237459. doi: 10.1155/2019/9237459. PMID: 31772787; PMCID: PMC6854983.

128: Oka S, Ogata M, Takano M, Minamimoto R, Hotta M, Tajima T, Nagata N, Tsukada K, Teruya K, Kikuchi Y, Gatanaga H; Cancer Screening in Hemophiliac/HIV Patient Study Group. Non-AIDS-defining malignancies in Japanese hemophiliacs with HIV-1 infection. Glob Health Med. 2019 Oct 31;1(1):49-54. doi: 10.35772/ghm.2019.01015. PMID: 33330754; PMCID: PMC7731093.

129: Onuchic L, Sato VAH, de Menezes Neves PDM, Balbo BEP, Portela-Neto AA, Ferreira FT, Watanabe EH, Watanabe A, de Almeida MCS, de Abreu Testagrossa L, Chocair PR, Onuchic LF. Two cases of fungal cyst infection in ADPKD: is this really a rare complication? BMC Infect Dis. 2019 Oct 29;19(1):911. doi: 10.1186/s12879-019-4444-y. PMID: 31664917; PMCID: PMC6819534.

130: Pesavento PA, Jackson K, Hampson TSTTB, Munday JS, Barrs VR, Beatty JA. A Novel Hepadnavirus is Associated with Chronic Hepatitis and Hepatocellular Carcinoma in Cats. Viruses. 2019 Oct 21;11(10):969. doi: 10.3390/v11100969. PMID: 31640283; PMCID: PMC6832243.

131: Hoang Oanh DT, Jung TY, Kim SK, Yang DH, Kang SR, Lee KH. Multiple Brain Biopsies for Epstein-Barr Virus-Positive Diffuse Large B-Cell Lymphoma with Extensive Necrosis in a Posttransplant Patient. World Neurosurg. 2020 Jan;133:10-13. doi: 10.1016/j.wneu.2019.09.084. Epub 2019 Sep 21. PMID: 31550543.

132: Greca RD, Cunha-Silva M, Costa LBE, Costa JGF, Mazo DFC, Sevá-Pereira T, Nascimento MMC, Pereira IE, Oliveira FC, Faria GAS, Neto FLP, Almeida JRS. Vanishing bile duct syndrome related to DILI and Hodgkin lymphoma overlap: A rare and severe case. Ann Hepatol. 2020 Jan-Feb;19(1):107-112. doi: 10.1016/j.aohep.2019.06.010. Epub 2019 Aug 19. PMID: 31537508.

133: Madney Y, Shalaby L, Elanany M, Adel N, Nasr E, Alsheshtawi K, Younes A, Hafez H. Clinical features and outcome of hepatosplenic fungal infections in children with haematological malignancies. Mycoses. 2020 Jan;63(1):30-37. doi: 10.1111/myc.13002. Epub 2019 Nov 13. PMID: 31514231.

134: Oliveira FR, Mamede M, Bizzi MF, Rocha ALL, Ferreira CN, Gomes KB, Cândido AL, Reis FM. Brown adipose tissue activity is reduced in women with polycystic ovary syndrome. Eur J Endocrinol. 2019 Nov;181(5):473-480. doi: 10.1530/EJE-19-0505. PMID: 31491743.

135: Simonds H, Botha MH, Ellmann A, Warwick J, Doruyter A, Neugut AI, Van Der Merwe H, Jacobson JS. HIV status does not have an impact on positron emission tomography-computed tomography (PET-CT) findings or radiotherapy treatment recommendations in patients with locally advanced cervical cancer. Int J Gynecol Cancer. 2019 Oct;29(8):1252-1257. doi: 10.1136/ijgc-2019-000641. Epub 2019 Aug 13. PMID: 31413068.

136: Dong ZR, Zhi XT, Li T. A Rapidly Progressing Polycystic Liver Disease? Gastroenterology. 2020 Jan;158(1):e9-e11. doi: 10.1053/j.gastro.2019.07.046. Epub 2019 Jul 31. PMID: 31376387.

137: Gad MM, Bazarbashi N, Kaur M, Gupta A. Sarcoid- like Phenomenon - ustekinumab induced granulomatous reaction mimicking diffuse metastatic disease: a case report and review of the literature. J Med Case Rep. 2019 Jul 30;13(1):257. doi: 10.1186/s13256-019-2137-1. PMID: 31358038; PMCID: PMC6664565.

138: Ito N, Yoshida T, Ichikawa R, Makino E, Akema S, Fukumori J, Takahashi N, Nakahara J, Yamashita R, Orihara K, Kobayashi M, Xiantao H, Watanabe Y, Mizukami S, Shibutani M. Clinical and pathological characteristics of acute myelogenous leukemia in a female koala with diabetes mellitus. J Vet Med Sci. 2019 Aug 24;81(8):1229-1233. doi: 10.1292/jvms.19-0006. Epub 2019 Jul 2. PMID: 31270282; PMCID: PMC6715919.

139: Martin WP, Vaughan LE, Yoshida K, Takahashi N, Edwards ME, Metzger A, Senum SR, Masyuk TV, LaRusso NF, Griffin MD, El-Zoghby Z, Harris PC, Kremers WK, Nagorney DM, Kamath PS, Torres VE, Hogan MC. Bacterial Cholangitis in Autosomal Dominant Polycystic Kidney and Liver Disease. Mayo Clin Proc Innov Qual Outcomes. 2019 May 27;3(2):149-159. doi: 10.1016/j.mayocpiqo.2019.03.004. PMID: 31193902; PMCID: PMC6543502.

140: Sarikaya I, Sarikaya A. Current Status of Radionuclide Renal Cortical Imaging in Pyelonephritis. J Nucl Med Technol. 2019 Dec;47(4):309-312. doi: 10.2967/jnmt.119.227942. Epub 2019 Jun 10. PMID: 31182659.

141: Islam Z, Hausauer B, Keshavamurthy J, Pucar D. Rapidly Lethal Ruptured Hepatocellular Carcinoma With Disseminated Peritoneal Carcinomatosis on FDG PET/CT. Clin Nucl Med. 2019 Nov;44(11):e622-e623. doi: 10.1097/RLU.0000000000002650. PMID: 31162259.

142: Hanssen O, Lovinfosse P, Weekers L, Hustinx R, Jouret F. La tomographie par émission de positons au 18F-FDG en pathologie rénale non oncologique : indications actuelles et perspectives [¹⁸F-FDG positron emission tomography in non-oncological renal pathology: Current indications and perspectives]. Nephrol Ther. 2019 Nov;15(6):430-438. French. doi: 10.1016/j.nephro.2018.11.007. Epub 2019 Apr 11. PMID: 30982747.

143: Zhang T, Zhang H, Fang T, Xu A, Chen MW. [Intermittent abdominal pain and abdominal distension with fatigue and massive ascites in a 13-year-old boy]. Zhongguo Dang Dai Er Ke Za Zhi. 2019 Mar;21(3):277-281. Chinese. doi: 10.7499/j.issn.1008-8830.2019.03.017. PMID: 30907354; PMCID: PMC7389353.

144: Singh G, Swamy A, Kumari K, Brijwal M, Damle NA, Das CJ, Bhowmik D. The Case | Mass in nonfunctioning first renal allograft in a recipient of 2 transplant kidneys. Kidney Int. 2019 Apr;95(4):1001-1002. doi: 10.1016/j.kint.2018.11.017. PMID: 30904055.

145: Lee S, Tsukasaki H, Yamauchi T. Visceral disseminated varicella zoster virus infection with brachial plexus neuritis detected by fluorodeoxyglucose positron emission tomography and computed tomography. J Infect Chemother. 2019 Jul;25(7):556-558. doi: 10.1016/j.jiac.2019.02.015. Epub 2019 Mar 14. PMID: 30879980.

146: Banshodani M, Marubayashi S, Shintaku S, Moriishi M, Tsuchiya S, Ohdan H, Kawanishi H. Isolated Pulmonary Cryptococcosis Confused with Lung Tumor 5 Years After Kidney Transplantation: A Case Report. Transplant Proc. 2019 Mar;51(2):561-564. doi: 10.1016/j.transproceed.2018.12.007. Epub 2018 Dec 12. PMID: 30879590.

147: Dejanovic D, Amtoft AG, Sørensen JB, Pøhl M. 18F-FDG PET/CT Findings in Disseminated Genital Herpes in an Immunocompetent Patient With Anaplastic Lymphoma Kinase Rearranged Advanced Nonsmall Cell Lung Cancer. Clin Nucl Med. 2019 May;44(5):e351-e352. doi: 10.1097/RLU.0000000000002523. PMID: 30829864.

148: Král Z, Adam Z, Folber F, Moulis M, Tomíška M, Říhová L, Štork M, Buliková A, Pour L, Krejčí M, Sandecká V, Koukalová R, Řehák Z, Čermáková Z. Systemic inflammatory response with high CRP values as the dominant symptom of multiple myeloma. Vnitr Lek. 2019 Winter;65(1):37-44. English. PMID: 30823836.

149: Braunwarth E, Primavesi F, Göbel G, Cardini B, Oberhuber R, Margreiter C, Maglione M, Schneeberger S, Öfner D, Stättner S. Is bile leakage after hepatic resection associated with impaired long-term survival? Eur J Surg Oncol. 2019 Jun;45(6):1077-1083. doi: 10.1016/j.ejso.2019.02.021. Epub 2019 Feb 21. PMID: 30803908.

150: Simsek FS, Akarsu S, Narin Y. Can we differentiate malignant peripheral nerve sheath tumor from benign neurofibroma without invasive sampling. World J Nucl Med. 2019 Jan-Mar;18(1):66-68. doi: 10.4103/wjnm.WJNM_11_18. PMID: 30774551; PMCID: PMC6357707.

151: Kavanal AJ, Bharati J, Vadi SK, Gupta KL, Kumar R, Mittal BR. 18F-FDG PET/CT Demonstrates Renal Cell Cancer in a Transplant With Synchronous Intestinal Infection. Clin Nucl Med. 2019 Apr;44(4):e294-e295. doi: 10.1097/RLU.0000000000002482. PMID: 30762817.

152: Zou S, Cheng Z, Song S, Zhu D, Zhu X. Telbivudine-Induced Myopathy Incidentally Detected by FDG PET/CT Imaging in a Patient With History of Hepatocellular Carcinoma. Clin Nucl Med. 2019 Feb;44(2):171-172. doi: 10.1097/RLU.0000000000002385. PMID: 30608913.

153: Rocha RD, Falsarella PM, Pereira De Azevedo AA, Garcia RG. Giant simple biliary cyst infection treated with minimally invasive percutaneous drainage.

World J Nucl Med. 2018 Oct-Dec;17(4):293-295. doi: 10.4103/wjnm.WJNM_63_17. PMID: 30505229; PMCID: PMC6216735.

154: Horvat N, Monti S, Oliveira BC, Rocha CCT, Giancipoli RG, Mannelli L. State of the art in magnetic resonance imaging of hepatocellular carcinoma. Radiol Oncol. 2018 Nov 26;52(4):353-364. doi: 10.2478/raon-2018-0044. PMID: 30511939; PMCID: PMC6287184.

155: Yugawa K, Yoshizumi T, Mano Y, Harada N, Itoh S, Ikegami T, Soejima Y, Fujita N, Kohashi K, Aishima S, Oda Y, Mori M. Primary intrahepatic cholangiocarcinoma with sarcomatous stroma: case report and review of the literature. Surg Case Rep. 2018 Nov 26;4(1):138. doi: 10.1186/s40792-018-0543-z. PMID: 30478801; PMCID: PMC6261094.

156: Bienz M, Tomaszewski M, McDonald EG. Severe pet-transmitted zoonosis in a patient with a compromised immune system. CMAJ. 2018 Nov 12;190(45):E1332-E1336. doi: 10.1503/cmaj.180720. PMID: 30420389; PMCID: PMC6232001.

157: Kim SE, Jung Y, Oh TH, Kim UJ, Kang SJ, Jang HC, Park KH, Lee KH, Jung SI. Case report: dual primary AIDS-defining cancers in an HIV-infected patient receiving antiretroviral therapy: Burkitt's lymphoma and Kaposi's sarcoma. BMC Cancer. 2018 Nov 8;18(1):1080. doi: 10.1186/s12885-018-5019-9. PMID: 30409111; PMCID: PMC6225729.

158: Eskian M, Alavi A, Khorasanizadeh M, Viglianti BL, Jacobsson H, Barwick TD, Meysamie A, Yi SK, Iwano S, Bybel B, Caobelli F, Lococo F, Gea J, Sancho-Muñoz A, Schildt J, Tatcı E, Lapa C, Keramida G, Peters M, Boktor RR, John J, Pitman AG, Mazurek T, Rezaei N. Effect of blood glucose level on standardized uptake value (SUV) in ¹⁸F- FDG PET-scan: a systematic review and meta-analysis of 20,807 individual SUV measurements. Eur J Nucl Med Mol Imaging. 2019 Jan;46(1):224-237. doi: 10.1007/s00259-018-4194-x. Epub 2018 Oct 22. PMID: 30350009.

159: Dickison P, Roger SD, Howard V, Smith SD. Beware what lies beneath: a case of a gigantic cutaneous squamous cell carcinoma. Clin Exp Dermatol. 2019 Jun;44(4):465-466. doi: 10.1111/ced.13791. Epub 2018 Sep 26. PMID: 30259551.

160: Murata Y, Hiramatsu K, Yoshida Y, Akazawa Y, Saito Y, Nosaka T, Ozaki Y, Hayama R, Takahashi K, Naito T, Ofuji K, Matsuda H, Ohtani M, Nemoto T, Nakamoto Y. Reactivation of intraabdominal tuberculous lymphadenopathy after drug-eluting beads transcatheter arterial chemoembolization in a patient with hepatocellular carcinoma. Clin J Gastroenterol. 2019 Feb;12(1):76-81. doi: 10.1007/s12328-018-0894-9. Epub 2018 Aug 23. PMID: 30141183.

161: Kim H, Yu Y, Shim KE, Kim JE, Koh J, Yoon JW, Ahn C, Oh YK. Esophageal

Artery Pseudoaneurysm and Takayasu Arteritis in a Patient with Autosomal Dominant Polycystic Kidney Disease. Electrolyte Blood Press. 2018 Jun;16(1):11-14. doi: 10.5049/EBP.2018.16.1.11. Epub 2018 Jun 30. PMID: 30046329; PMCID: PMC6051944.

162: Iliescu L, Mercan-Stanciu A, Ioanitescu ES, Toma L. Hepatitis C-Associated B-cell Non-Hodgkin Lymphoma: A Pictorial Review. Ultrasound Q. 2018 Sep;34(3):156-166. doi: 10.1097/RUQ.0000000000000369. PMID: 30020272.

163: Kim MH, Kim DW. Incidental Detection of Peri-Renal Hemorrhage on F-18 FDG PET/CT Imaging in a Patient with Polycystic Kidney Disease. Nucl Med Mol Imaging. 2018 Jun;52(3):243-246. doi: 10.1007/s13139-018-0513-1. Epub 2018 Feb 1. PMID: 29942405; PMCID: PMC5995777.

164: Ezeh U, Huang A, Landay M, Azziz R. Long-Term Response of Hirsutism and Other Hyperandrogenic Symptoms to Combination Therapy in Polycystic Ovary Syndrome. J Womens Health (Larchmt). 2018 Jul;27(7):892-902. doi: 10.1089/jwh.2017.6833. Epub 2018 Jun 7. PMID: 29878857; PMCID: PMC6065519.

165: Chuang YW, Yu TM, Huang ST, Sun KT, Lo YC, Fu PK, Lee BJ, Chen CH, Lin CL, Kao CH. Young-Adult Polycystic Kidney Disease is Associated with Major Cardiovascular Complications. Int J Environ Res Public Health. 2018 May 3;15(5):903. doi: 10.3390/ijerph15050903. PMID: 29751520; PMCID: PMC5981942.

166: Zhang JW, Yang HY, Xu YY, Sang XT, Yu SN, Huang HC, Bian J, Xiong JP, Lu X. Surgical treatment for metastasis from lymphoepithelioma-like cholangiocarcinoma in the liver: A case report. Medicine (Baltimore). 2018 May;97(19):e0666. doi: 10.1097/MD.0000000000010666. PMID: 29742707; PMCID: PMC5959422.

167: Pijl JP, Glaudemans AWJM, Slart RHJA, Kwee TC. ¹⁸F-FDG PET/CT in Autosomal Dominant Polycystic Kidney Disease Patients with Suspected Cyst Infection. J Nucl Med. 2018 Nov;59(11):1734-1741. doi: 10.2967/jnumed.117.199448. Epub 2018 Apr 13. PMID: 29653972.

168: Casillas-Sagrado E, Burguera V, Rioja-Martín ME, Rivera-Gorrín M. Infected renal cyst presented with pleural effusion in a woman with autosomal dominant polycystic kidney disease. Clin Kidney J. 2018 Apr;11(2):204-206. doi: 10.1093/ckj/sfx098. Epub 2017 Aug 31. PMID: 29644060; PMCID: PMC5888413.

169: Watanabe T, Araki K, Ishii N, Igarashi T, Watanabe A, Kubo N, Kuwano H, Shirabe K. A Surgically Resected Pancreatic Schwannoma with Obstructive Jaundice with Special Reference to Differential Diagnosis from Other Cystic Lesions in the Pancreas. Case Rep Gastroenterol. 2018 Feb 21;12(1):85-91. doi: 10.1159/000485559. PMID: 29606941; PMCID: PMC5869564.

170: Pijl JP, Kwee TC, Slart RHJA, Glaudemans AWJM. FDG-PET/CT for diagnosis of cyst infection in autosomal dominant polycystic kidney disease. Clin Transl Imaging. 2018;6(1):61-67. doi: 10.1007/s40336-017-0261-8. Epub 2018 Feb 12. PMID: 29568734; PMCID: PMC5860106.

171: Chalaye J, Costentin CE, Luciani A, Amaddeo G, Ganne-Carrié N, Baranes L, Allaire M, Calderaro J, Azoulay D, Nahon P, Seror O, Mallat A, Soussan M, Duvoux C, Itti E, Nault JC. Positron emission tomography/computed tomography with 18F-fluorocholine improve tumor staging and treatment allocation in patients with hepatocellular carcinoma. J Hepatol. 2018 Aug;69(2):336-344. doi: 10.1016/j.jhep.2018.02.018. Epub 2018 Mar 6. PMID: 29518452.

172: Bao C, Wei J, Zhao X, Lin L, Chen D, Liu K, Qian W, Anas JM, Zhao K. Prognostic value of fluorine-18-fluorodeoxyglucose positron emission tomography/computed tomography in primary hepatic mucosa-associated lymphoid tissue lymphoma: A case report and review of the literature. Medicine (Baltimore). 2018 Mar;97(10):e9877. doi: 10.1097/MD.0000000000009877. PMID: 29517697; PMCID: PMC5882437.

173: Yang Y, Wang W, Kan Y, Liu J, Yang J. FDG PET/CT Findings of Polymorphic Posttransplant Lymphoproliferative Disorders in a Transplanted Kidney. Clin Nucl Med. 2018 Jun;43(6):441-444. doi: 10.1097/RLU.0000000000002027. PMID: 29485445.

174: Aida T, Shiobara M, Wakatsuki K, Arai S, Suda K, Miyazawa K, Miyoshi T, Takahashi Y, Yoshioka S. [A Case of Transverse Colon Cancer with Liver Metastasis and Tumor Thrombosis of Portal Vein Effectively Treated with Chemotherapy]. Gan To Kagaku Ryoho. 2018 Feb;45(2):374-376. Japanese. PMID: 29483452.

175: Aktaş GE, Sarıkaya A, Demir SS. Diffusely Increased Splenic Fluorodeoxyglucose Uptake in Lung Cancer Patients. Turk Thorac J. 2017 Jan;18(1):6-10. doi: 10.5152/TurkThoracJ.2017.16025. Epub 2017 Jan 1. PMID: 29404150; PMCID: PMC5783169.

176: Sekiya M, Sakamoto K, Matsukuma S, Tokuhisa Y, Tokumitsu Y, Matsui H, Kanekiyo S, Tomochika S, Iida M, Takeda S, Suzuki N, Yoshino S, Hazama S, Hoshii Y, Nagano H. [A Case of Intrahepatic Cholangiocarcinoma with Ductal Plate Malformation Pattern]. Gan To Kagaku Ryoho. 2017 Nov;44(12):1877-1879. Japanese. PMID: 29394806.

177: Fujiwara S, Tsuyoshi H, Nishimura T, Takahashi N, Yoshida Y. Precise preoperative diagnosis of struma ovarii with pseudo-Meigs' syndrome mimicking ovarian cancer with the combination of ¹³¹I scintigraphy and ¹⁸F-FDG PET: case report and review of the literature. J Ovarian Res. 2018 Feb 2;11(1):11. doi: 10.1186/s13048-018-0383-2. PMID: 29391043; PMCID:

PMC5796437.

178: Meletani T, Cantini L, Lanese A, Nicolini D, Cimadamore A, Agostini A, Ricci G, Antognoli S, Mandolesi A, Guido M, Alaggio R, Giuseppetti GM, Scarpelli M, Vivarelli M, Berardi R. Are liver nested stromal epithelial tumors always low aggressive? World J Gastroenterol. 2017 Dec 14;23(46):8248-8255. doi: 10.3748/wjg.v23.i46.8248. PMID: 29290661; PMCID: PMC5739931.

179: Chen D, Dong M, Zhao K, Sun F, Wang H, Liu Z. Unusual synchronous liver and brain abscesses infected by rare Aerococcus viridians in a patient with pulmonary arteriovenous malformations on FDG PET/CT: A case report and literature review. Medicine (Baltimore). 2017 Dec;96(49):e9048. doi: 10.1097/MD.0000000000009048. PMID: 29245306; PMCID: PMC5728921.

180: Foppiani L, Panarello S, Filauro M, Scirocco MC, Cappato S, Parodi A, Sola S, Antonucci G. Insulinoma and Chronic Kidney Disease: An Uncommon Conundrum Not to Be Overlooked. Clin Med Insights Endocrinol Diabetes. 2017 Nov 20;10:1179551417742620. doi: 10.1177/1179551417742620. PMID: 29200897; PMCID: PMC5700780.

181: Zhou P, Tu L, Lin X, Hao X, Zheng Q, Zeng W, Zhang X, Zheng Y, Wang L, Li S. cfa-miR-143 Promotes Apoptosis via the p53 Pathway in Canine Influenza Virus H3N2-Infected Cells. Viruses. 2017 Nov 25;9(12):360. doi: 10.3390/v9120360. PMID: 29186842; PMCID: PMC5744135.

182: Albano D, Bosio G, Bertoli M, Giubbini R, Bertagna F. 18F-FDG PET/CT in primary brain lymphoma. J Neurooncol. 2018 Feb;136(3):577-583. doi: 10.1007/s11060-017-2686-3. Epub 2017 Nov 17. PMID: 29147860.

183: Li Z, Sun H, Shen C, Yang C, Wang Y, Wang W, Jia Y, Zhao Q, Zhao C. Primary hepatic angiosarcoma with spleen metastases in an adult woman: a case report and literature review. Int J Clin Exp Pathol. 2017 Nov 1;10(11):11211-11218. PMID: 31966472; PMCID: PMC6965857.

184: Parghane RV, Phulsunga RK, Gupta R, Basher RK, Bhattacharya A, Mittal BR. Usefulness of Tc99m-mebrofenin Hepatobiliary Scintigraphy and Single Photon Emission Computed Tomography/Computed Tomography in the Diagnosis of Bronchobiliary Fistula. World J Nucl Med. 2017 Oct-Dec;16(4):317-319. doi: 10.4103/1450-1147.215490. PMID: 29033682; PMCID: PMC5639450.

185: Al Shakarchi J, Neil D, Lipkin G, Elsharkawy A, Inston N. Identification of occult active infection using PET-CT in a combined liver-kidney transplant candidate. Transpl Infect Dis. 2017 Dec;19(6). doi: 10.1111/tid.12776. Epub 2017 Nov 3. PMID: 28921746.

186: Shimamura Y, Takizawa H. Diagnostic utility of ¹⁸FDG-PET/CT for ADPKD cyst infection. Clin Exp Nephrol. 2018 Aug;22(4):973-974. doi: 10.1007/s10157-017-1482-x. Epub 2017 Sep 14. PMID: 28913675.

187: Biassoni L, Easty M. Paediatric nuclear medicine imaging. Br Med Bull. 2017 Sep 1;123(1):127-148. doi: 10.1093/bmb/ldx025. PMID: 28910997.

188: Liang L, Zhou N, Xu H, Liu D, Lu Y, Li F, Guo J. Urachal mucinous adenocarcinoma with pseudomyxoma peritonei: A case report. Medicine (Baltimore). 2017 Sep;96(35):e7548. doi: 10.1097/MD.0000000000007548. PMID: 28858081; PMCID: PMC5585475.

189: Yu TM, Chuang YW, Sun KT, Yu MC, Kung SC, Lee BK, Huang ST, Chen CH, Lin CL, Kao CH. Polycystic kidney disease is significantly associated with dementia risk. Neurology. 2017 Oct 3;89(14):1457-1463. doi: 10.1212/WNL.0000000000004434. Epub 2017 Aug 30. PMID: 28855402.

190: Wróbel MP, Marek B, Kajdaniuk D, Rokicka D, Szymborska-Kajanek A, Strojek K. Metformin - a new old drug. Endokrynol Pol. 2017;68(4):482-496. doi: 10.5603/EP.2017.0050. PMID: 28819951.

191: Park S, Lee D, Lee KM, Han SU, Lee JH, Lee SJ, An YS, Yoon JK. Clinical usefulness of F-18 FDG PET in lymphoepithelioma-like gastric carcinoma. Eur J Radiol. 2017 Sep;94:160-166. doi: 10.1016/j.ejrad.2017.06.022. Epub 2017 Jun 27. PMID: 28712702.

192: Hardy LE, Hessamodini H, Wallace M, Francis R. Is FCH PET able to identify foci of infection superiorly to FDG PET? BMJ Case Rep. 2017 Jul 14;2017:bcr2017219944. doi: 10.1136/bcr-2017-219944. PMID: 28710238; PMCID: PMC5534810.

193: Wong LL, Reyes RJ, Kwee SA, Hernandez BY, Kalathil SC, Tsai NC. Pitfalls in surveillance for hepatocellular carcinoma: How successful is it in the real world? Clin Mol Hepatol. 2017 Sep;23(3):239-248. doi: 10.3350/cmh.2017.0008. Epub 2017 Jul 14. PMID: 28706177; PMCID: PMC5628007.

194: Costa D, Fernandes D, Furtado A, Santa Cruz A. Cytomegalovirus duodenitis in immunocompetent patients: what else should we look for? BMJ Case Rep. 2017 Jun 13;2017:bcr2017219679. doi: 10.1136/bcr-2017-219679. PMID: 28611166; PMCID: PMC5534848.

195: Kim H, Oh YK, Park HC, Park S, Lee S, Lee HY, Hwang YH, Ahn C. Clinical experience with white blood cell-PET/CT in autosomal dominant polycystic kidney disease patients with suspected cyst infection: A prospective case series. Nephrology (Carlton). 2018 Jul;23(7):661-668. doi: 10.1111/nep.13080. PMID:

28565035.

196: Krishnan N, Perazella MA. The Role of PET Scanning in the Evaluation of Patients With Kidney Disease. Adv Chronic Kidney Dis. 2017 May;24(3):154-161. doi: 10.1053/j.ackd.2017.01.002. PMID: 28501078.

197: Cingam S, Patel S, Koshy N. A Case of Hepatosplentic T Cell Lymphoma - A Rare, Aggressive Tumor of the Young. J La State Med Soc. 2017 Mar-Apr;169(2):49-50. Epub 2017 Apr 15. PMID: 28414669.

198: Nohr EW, Itani DM, Andrews CN, Kelly MM. Varicella-Zoster Virus Gastritis: Case Report and Review of the Literature. Int J Surg Pathol. 2017 Aug;25(5):449-452. doi: 10.1177/1066896917696751. Epub 2017 Mar 1. PMID: 28381144.

199: Ehman EC, Johnson GB, Villanueva-Meyer JE, Cha S, Leynes AP, Larson PEZ, Hope TA. PET/MRI: Where might it replace PET/CT? J Magn Reson Imaging. 2017 Nov;46(5):1247-1262. doi: 10.1002/jmri.25711. Epub 2017 Mar 30. PMID: 28370695; PMCID: PMC5623147.

200: Diamandis EP, Stanczyk FZ, Wheeler S, Mathew A, Stengelin M, Nikolenko G, Glezer EN, Brown MD, Zheng Y, Chen YH, Wu HL, Azziz R. Serum complexed and free prostate-specific antigen (PSA) for the diagnosis of the polycystic ovarian syndrome (PCOS). Clin Chem Lab Med. 2017 Oct 26;55(11):1789-1797. doi: 10.1515/cclm-2016-1124. PMID: 28361781; PMCID: PMC5742006.

201: Şamdanci E, Şahin N, Dağli AF, Akatli AN, Aydin NE. Fascioliasis: A Rare Parasitic Infection-Mimicking Tumor in the Liver: Report of Two Cases. Turk Patoloji Derg. 2019;35(1):58-60. English. doi: 10.5146/tjpath.2016.01367. PMID: 28272650.

202: Gucer F, Misirlioglu S, Ceydeli N, Taskiran C. Robot-assisted laparoscopic transperitoneal infrarenal lymphadenectomy in patients with locally advanced cervical cancer by single docking: Do we need a backup procedure? J Robot Surg. 2018 Mar;12(1):49-58. doi: 10.1007/s11701-017-0685-1. Epub 2017 Mar 2. PMID: 28255734.

203: Hasegawa S, Kato H, Hamasaki Y, Koiwa T, Nangaku M. Positron emission tomography in the diagnosis of cyst infections. Kidney Int. 2017 Mar;91(3):759. doi: 10.1016/j.kint.2016.10.025. PMID: 28202176.

204: Kumar SK, Callander NS, Alsina M, Atanackovic D, Biermann JS, Chandler JC, Costello C, Faiman M, Fung HC, Gasparetto C, Godby K, Hofmeister C, Holmberg L, Holstein S, Huff CA, Kassim A, Liedtke M, Martin T, Omel J, Raje N, Reu FJ, Singhal S, Somlo G, Stockerl-Goldstein K, Treon SP, Weber D, Yahalom J, Shead

DA, Kumar R. Multiple Myeloma, Version 3.2017, NCCN Clinical Practice Guidelines in Oncology. J Natl Compr Canc Netw. 2017 Feb;15(2):230-269. doi: 10.6004/jnccn.2017.0023. PMID: 28188192.

205: Bhamidimarri KR, Lanza DC, Kashikar ND. Hepatobiliary and Pancreatic: A unique case of HPV related metastatic Schneiderian carcinoma presenting as polycystic liver disease. J Gastroenterol Hepatol. 2017 Jan;32(1):10. doi: 10.1111/jgh.13538. PMID: 28052462.

206: Parta M, Cuellar-Rodriguez J, Freeman AF, Gea-Banacloche J, Holland SM, Hickstein DD. Resolution of Multifocal Epstein-Barr Virus-Related Smooth Muscle Tumor in a Patient with GATA2 Deficiency Following Hematopoietic Stem Cell Transplantation. J Clin Immunol. 2017 Jan;37(1):61-66. doi: 10.1007/s10875-016-0360-8. Epub 2016 Dec 6. PMID: 27924436; PMCID: PMC6334651.

207: Ikeshima R, Nishimura J, Takahashi H, Haraguchi N, Hata T, Mizushima T, Doki Y, Mori M. A case of adenocarcinoma arising in an ileal diverticulum resected by laparoscopic surgery. Surg Case Rep. 2016 Dec;2(1):135. doi: 10.1186/s40792-016-0257-z. Epub 2016 Nov 17. PMID: 27858376; PMCID: PMC5114209.

208: Yu TM, Chuang YW, Yu MC, Chen CH, Kao CH. Risk of cancer in patients with polycystic kidney disease - Authors' reply. Lancet Oncol. 2016 Nov;17(11):e476. doi: 10.1016/S1470-2045(16)30528-9. PMID: 27819243.

209: Albano D, Bosio G, Bertagna F. 18F-FDG PET/CT demonstrated renal and hepatic cyst infection in a patient with autosomal dominant polycystic kidney disease. Nucl Med Rev Cent East Eur. 2016;19(B):26-28. doi: 10.5603/NMR.2016.0035. PMID: 27813615.

210: Jørgensen NP, Alstrup AK, Mortensen FV, Knudsen K, Jakobsen S, Madsen LB, Bender D, Breining P, Petersen MS, Schleimann MH, Dagnæs-Hansen F, Gormsen LC, Borghammer P. Cholinergic PET imaging in infections and inflammation using ¹¹C-donepezil and ¹⁸F-FEOBV. Eur J Nucl Med Mol Imaging. 2017 Mar;44(3):449-458. doi: 10.1007/s00259-016-3555-6. Epub 2016 Oct 26. PMID: 27785538.

211: Obed A, Bashir A, Jarrad A. Rapid Virological Response After Early Treatment with a Combined Therapy of Ledipasvir and Sofosbuvir in HCV Genotype 4 After Living Donor Liver Transplantation in a HCC Downstaged Patient: Case Report and Review of the Literature. Am J Case Rep. 2016 Sep 20;17:672-5. doi: 10.12659/ajcr.898594. PMID: 27647003; PMCID: PMC5031172.

212: Albano D, Bertoli M, Ferro P, Fallanca F, Gianolli L, Picchio M, Giubbini R, Bertagna F. 18F-FDG PET/CT in gastric MALT lymphoma: a bicentric experience. Eur J Nucl Med Mol Imaging. 2017 Apr;44(4):589-597. doi:

10.1007/s00259-016-3518-y. Epub 2016 Sep 12. PMID: 27619357.

213: Lantinga MA, Darding AJ, de Sévaux RG, Alam A, Bleeker-Rovers CP, Bobot M, Cornec-Le Gall E, Gevers TJ, Hassoun Z, Meijer E, Mrug M, Nevens F, Onuchic LF, Pei Y, Piccoli GB, Pirson Y, Rangan GK, Torra R, Visser FW, Jouret F, Kanaan N, Oyen WJ, Suwabe T, Torres VE, Drenth JP; Delphi collaborators. International Multi-Specialty Delphi Survey: Identification of Diagnostic Criteria for Hepatic and Renal Cyst Infection. Nephron. 2016;134(4):205-214. doi: 10.1159/000446664. Epub 2016 Sep 3. PMID: 27599120.

214: Yu TM, Chuang YW, Yu MC, Chen CH, Yang CK, Huang ST, Lin CL, Shu KH, Kao CH. Risk of cancer in patients with polycystic kidney disease: a propensity-score matched analysis of a nationwide, population-based cohort study. Lancet Oncol. 2016 Oct;17(10):1419-1425. doi: 10.1016/S1470-2045(16)30250-9. Epub 2016 Aug 20. PMID: 27550645.

215: Xie J, Zhang XB, Wang WZ, Li HZ. Case report of renal pelvis squamous cell carcinoma with tumor embolus in autosomal dominant polycystic kidney disease. Medicine (Baltimore). 2016 Aug;95(33):e4597. doi: 10.1097/MD.0000000000004597. PMID: 27537592; PMCID: PMC5370818.

216: Neuville M, Hustinx R, Jacques J, Krzesinski JM, Jouret F. Diagnostic Algorithm in the Management of Acute Febrile Abdomen in Patients with Autosomal Dominant Polycystic Kidney Disease. PLoS One. 2016 Aug 16;11(8):e0161277. doi: 10.1371/journal.pone.0161277. PMID: 27529555; PMCID: PMC4987061.

217: Jadoul A, Lovinfosse P, Weekers L, Delanaye P, Krzesinski JM, Hustinx R, Jouret F. The Uptake of 18F-FDG by Renal Allograft in Kidney Transplant Recipients Is Not Influenced by Renal Function. Clin Nucl Med. 2016 Sep;41(9):683-7. doi: 10.1097/RLU.0000000000001298. PMID: 27405040.

218: Kaido T. Selection Criteria and Current Issues in Liver Transplantation for Hepatocellular Carcinoma. Liver Cancer. 2016 Apr;5(2):121-7. doi: 10.1159/000367749. Epub 2016 Mar 17. PMID: 27386430; PMCID: PMC4906426.

219: Tamai Y, Tanaka H, Yoshikawa K, Sugimoto R, Higashitani K, Morooka R, Yamamoto N, Hasegawa H, Iwasa M, Takei Y. [A case of a solitary Mycobacterium avium complex lesion of the liver]. Nihon Shokakibyo Gakkai Zasshi. 2016 Jul;113(7):1230-5. Japanese. doi: 10.11405/nisshoshi.113.1230. PMID: 27383107.

220: O'Neill AF, Adil EA, Irace AL, Neff L, Davis IJ, Perez-Atayde AR, Voss SD, Weinberg O, Rahbar R. Post-transplant lymphoproliferative disorder of the pediatric airway: Presentation and management. Int J Pediatr Otorhinolaryngol. 2016 Jul;86:218-23. doi: 10.1016/j.ijporl.2016.04.035. Epub 2016 Apr 30. PMID: 27260610.

221: Song MA, Kwee SA, Tiirikainen M, Hernandez BY, Okimoto G, Tsai NC, Wong LL, Yu H. Comparison of genome-scale DNA methylation profiles in hepatocellular carcinoma by viral status. Epigenetics. 2016 Jun 2;11(6):464-74. doi: 10.1080/15592294.2016.1151586. Epub 2016 Jun 1. PMID: 27248055; PMCID: PMC4939927.

222: Foster WR, Bischin A, Dorer R, Aboulafia DM. Human Herpesvirus Type 8-associated Large B-cell Lymphoma: A Nonserous Extracavitary Variant of Primary Effusion Lymphoma in an HIV-infected Man: A Case Report and Review of the Literature. Clin Lymphoma Myeloma Leuk. 2016 Jun;16(6):311-21. doi: 10.1016/j.clml.2016.03.013. Epub 2016 Apr 1. PMID: 27234438; PMCID: PMC5421557.

223: Garvey WT, Mechanick JI, Brett EM, Garber AJ, Hurley DL, Jastreboff AM, Nadolsky K, Pessah-Pollack R, Plodkowski R; Reviewers of the AACE/ACE Obesity Clinical Practice Guidelines. AMERICAN ASSOCIATION OF CLINICAL ENDOCRINOLOGISTS AND AMERICAN COLLEGE OF ENDOCRINOLOGY COMPREHENSIVE CLINICAL PRACTICE GUIDELINES
FOR MEDICAL CARE OF PATIENTS WITH OBESITY. Endocr Pract. 2016 Jul;22 Suppl 3:1-203. doi: 10.4158/EP161365.GL. Epub 2016 May 24. PMID: 27219496.

224: Langsteger W, Rezaee A, Loidl W, Geinitz HS, Fitz F, Steinmair M, Broinger G, Pallwien-Prettner L, Beheshti M, Imamovic L, Beheshti M, Rendl G, Hackl D, Tsybrovsky O, Steinmair M, Emmanuel K, Moinfar F, Pirich C, Langsteger W, Bytyqi A, Karanikas G, Mayerhöfer M, Koperek O, Niederle B, Hartenbach M, Beyer T, Herrmann K, Czernin J, Rausch I, Rust P, DiFranco MD, Lassen M, Stadlbauer A, Mayerhöfer ME, Hartenbach M, Hacker M, Beyer T, Binzel K, Magnussen R, Wei W, Knopp MU, Flanigan DC, Kaeding C, Knopp MV, Leisser A, Nejabat M, Hartenbach M, Kramer G, Krainer M, Hacker M, Haug A, Lehnert W, Schmidt K, Kimiaei S, Bronzel M, Kluge A, Wright CL, Binzel K, Zhang J, Wuthrick E, Maniawski P, Knopp MV, Blaickner M, Rados E, Huber A, Dulovits M, Kulkarni H, Wiessalla S, Schuchardt C, Baum RP, Knäusl B, Georg D, Bauer M, Wulkersdorfer B, Wadsak W, Philippe C, Haslacher H, Zeitlinger M, Langer O, Bauer M, Feldmann M, Karch R, Wadsak W, Zeitlinger M, Koepp MJ, Asselin MC, Pataraia E, Langer O, Zeilinger M, Philippe C, Dumanic M, Pichler F, Pilz J, Hacker M, Wadsak W, Mitterhauser M, Nics L, Steiner B, Hacker M, Mitterhauser M, Wadsak W, Traxl A, Wanek T, Kryeziu K, Mairinger S, Stanek J, Berger W, Kuntner C, Langer O, Mairinger S, Wanek T, Traxl A, Krohn M, Stanek J, Filip T, Sauberer M, Kuntner C, Pahnke J, Langer O, Svatunek D, Denk C, Wilkovitsch M, Wanek T, Filip T, Kuntner-Hannes C, Fröhlich J, Mikula H, Denk C, Svatunek D, Wanek T, Mairinger S, Stanek J, Filip T, Fröhlich J, Mikula H, Kuntner-Hannes C, Balber T, Singer J, Fazekas J, Rami-Mark C, Berroterán-Infante N, Jensen-Jarolim E, Wadsak W, Hacker M, Viernstein H, Mitterhauser M, Denk C, Svatunek D, Sohr B, Mikula H, Fröhlich J, Wanek T, Kuntner-Hannes C, Filip T, Pfaff S, Philippe C, Mitterhauser M, Hartenbach M, Hacker M, Wadsak W, Wanek T, Halilbasic E, Visentin M, Mairinger S, Stieger B,

Kuntner C, Trauner M, Langer O, Lam P, Aistleitner M, Eichinger R, Artner C, Eidherr H, Vraka C, Haug A, Mitterhauser M, Nics L, Hartenbach M, Hacker M, Wadsak W, Kvaternik H, Müller R, Hausberger D, Zink C, Aigner RM, Cossío U, Asensio M, Montes A, Akhtar S, Te Welscher Y, van Nostrum R, Gómez-Vallejo V, Llop J, VandeVyver F, Barclay T, Lippens N, Troch M, Hehenwarter L, Egger B, Holzmannhofer J, Rodrigues-Radischat M, Pirich C, Pötsch N, Rausch I, Wilhelm D, Weber M, Furtner J, Karanikas G, Wöhrer A, Mitterhauser M, Hacker M, Traub-Weidinger T, Cassou-Mounat T, Balogova S, Nataf V, Calzada M, Huchet V, Kerrou K, Devaux JY, Mohty M, Garderet L, Talbot JN, Stanzel S, Pregartner G, Schwarz T, Bjelic-Radisic V, Liegl-Atzwanger B, Aigner R, Stanzel S, Quehenberger F, Aigner RM, Marković AK, Janković M, Jerković VM, Paskaš M, Pupić G, Džodić R, Popović D, Fornito MC, Familiari D, Koranda P, Polzerová H, Metelková I, Henzlová L, Formánek R, Buriánková E, Kamínek M, Thomson WH, Lewis C, Thomson WH, O'Brien J, James G, Notghi A, Huber H, Stelzmüller I, Wunn R, Mandl M, Fellner F, Lamprecht B, Gabriel M, Fornito MC, Leonardi G, Thomson WH, O'Brien J, James G, Hudzietzová J, Sabol J, Fülöp M. 32nd International Austrian Winter Symposium : Zell am See, the Netherlands. 20-23 January 2016. EJNMMI Res. 2016 Apr;6(Suppl 1):32. doi: 10.1186/s13550-016-0168-9. Epub 2016 Apr 18. PMID: 27090254; PMCID: PMC4835428.

225: Bulakçı M, Kartal MG, Yılmaz S, Yılmaz E, Yılmaz R, Şahin D, Aşık M, Erol OB. Multimodality imaging in diagnosis and management of alveolar echinococcosis: an update. Diagn Interv Radiol. 2016 May-Jun;22(3):247-56. doi: 10.5152/dir.2015.15456. PMID: 27082120; PMCID: PMC4859741.

226: Jaster A, Wachsmann J. Serendipitous discovery of peritoneal mesothelioma. Proc (Bayl Univ Med Cent). 2016 Apr;29(2):185-7. doi: 10.1080/08998280.2016.11929410. PMID: 27034564; PMCID: PMC4790566.

227: Kwon HW, Lee HY, Hwang YH, Park HC, Ahn C, Kang KW. Diagnostic performance of 18F-FDG-labeled white blood cell PET/CT for cyst infection in patients with autosomal dominant polycystic kidney disease: a prospective study. Nucl Med Commun. 2016 May;37(5):493-8. doi: 10.1097/MNM.0000000000000466. PMID: 27014954.

228: Loizidou A, Aoun M, Klastersky J. Fever of unknown origin in cancer patients. Crit Rev Oncol Hematol. 2016 May;101:125-30. doi: 10.1016/j.critrevonc.2016.02.015. Epub 2016 Feb 27. PMID: 26995082.

229: Raslan OA, Parkar ND, Muzaffar R, Doherty C, Osman MM. Case 227: Endobronchial Carcinoid Tumor with Incidental Metastatic Breast Cancer Detected with Somatostatin Receptor Scintigraphy ((111)In Pentreotide). Radiology. 2016 Mar;278(3):949-55. doi: 10.1148/radiol.2016141475. PMID: 26885736.

230: Mar WA, Shon AM, Lu Y, Yu JH, Berggruen SM, Guzman G, Ray CE Jr, Miller F. Imaging spectrum of cholangiocarcinoma: role in diagnosis, staging, and

posttreatment evaluation. Abdom Radiol (NY). 2016 Mar;41(3):553-67. doi: 10.1007/s00261-015-0583-9. PMID: 26847022.

231: Yu TM, Chuang YW, Yu MC, Huang ST, Chou CY, Lin CL, Chiu CC, Kao CH. New-onset Atrial Fibrillation is Associated With Polycystic Kidney Disease: A Nationwide Population-based Cohort Study. Medicine (Baltimore). 2016 Jan;95(4):e2623. doi: 10.1097/MD.0000000000002623. PMID: 26825919; PMCID: PMC5291589.

232: Klionsky DJ, Abdelmohsen K, Abe A, Abedin MJ, Abeliovich H, Acevedo Arozena A, Adachi H, Adams CM, Adams PD, Adeli K, Adhihetty PJ, Adler SG, Agam G, Agarwal R, Aghi MK, Agnello M, Agostinis P, Aguilar PV, Aguirre-Ghiso J, Airoldi EM, Ait-Si-Ali S, Akematsu T, Akporiaye ET, Al-Rubeai M, Albaiceta GM, Albanese C, Albani D, Albert ML, Aldudo J, Algül H, Alirezaei M, Alloza I, Almasan A, Almonte-Beceril M, Alnemri ES, Alonso C, Altan-Bonnet N, Altieri DC, Alvarez S, Alvarez-Erviti L, Alves S, Amadoro G, Amano A, Amantini C, Ambrosio S, Amelio I, Amer AO, Amessou M, Amon A, An Z, Anania FA, Andersen SU, Andley UP, Andreadi CK, Andrieu-Abadie N, Anel A, Ann DK, Anoopkumar-Dukie S, Antonioli M, Aoki H, Apostolova N, Aquila S, Aquilano K, Araki K, Arama E, Aranda A, Araya J, Arcaro A, Arias E, Arimoto H, Ariosa AR, Armstrong JL, Arnould T, Arsov I, Asanuma K, Askanas V, Asselin E, Atarashi R, Atherton SS, Atkin JD, Attardi LD, Auberger P, Auburger G, Aurelian L, Autelli R, Avagliano L, Avantaggiati ML, Avrahami L, Awale S, Azad N, Bachetti T, Backer JM, Bae DH, Bae JS, Bae ON, Bae SH, Baehrecke EH, Baek SH, Baghdiguian S, Bagniewska-Zadworna A, Bai H, Bai J, Bai XY, Bailly Y, Balaji KN, Balduini W, Ballabio A, Balzan R, Banerjee R, Bánhegyi G, Bao H, Barbeau B, Barrachina MD, Barreiro E, Bartel B, Bartolomé A, Bassham DC, Bassi MT, Bast RC Jr, Basu A, Batista MT, Batoko H, Battino M, Bauckman K, Baumgarner BL, Bayer KU, Beale R, Beaulieu JF, Beck GR Jr, Becker C, Beckham JD, Bédard PA, Bednarski PJ, Begley TJ, Behl C, Behrends C, Behrens GM, Behrns KE, Bejarano E, Belaid A, Belleudi F, Bénard G, Berchem G, Bergamaschi D, Bergami M, Berkhout B, Berliocchi L, Bernard A, Bernard M, Bernassola F, Bertolotti A, Bess AS, Besteiro S, Bettuzzi S, Bhalla S, Bhattacharyya S, Bhutia SK, Biagosch C, Bianchi MW, Biard-Piechaczyk M, Billes V, Bincoletto C, Bingol B, Bird SW, Bitoun M, Bjedov I, Blackstone C, Blanc L, Blanco GA, Blomhoff HK, Boada-Romero E, Böckler S, Boes M, Boesze-Battaglia K, Boise LH, Bolino A, Boman A, Bonaldo P, Bordi M, Bosch J, Botana LM, Botti J, Bou G, Bouché M, Bouchecareilh M, Boucher MJ, Boulton ME, Bouret SG, Boya P, Boyer-Guittaut M, Bozhkov PV, Brady N, Braga VM, Brancolini C, Braus GH, Bravo-San Pedro JM, Brennan LA, Bresnick EH, Brest P, Bridges D, Bringer MA, Brini M, Brito GC, Brodin B, Brookes PS, Brown EJ, Brown K, Broxmeyer HE, Bruhat A, Brum PC, Brumell JH, Brunetti-Pierri N, Bryson-Richardson RJ, Buch S, Buchan AM, Budak H, Bulavin DV, Bultman SJ, Bultynck G, Bumbasirevic V, Burelle Y, Burke RE, Burmeister M, Bütikofer P, Caberlotto L, Cadwell K, Cahova M, Cai D, Cai J, Cai Q, Calatayud S, Camougrand N, Campanella M, Campbell GR, Campbell M, Campello S, Candau R, Caniggia I, Cantoni L, Cao L, Caplan AB, Caraglia M, Cardinali C, Cardoso SM, Carew JS,

Carleton LA, Carlin CR, Carloni S, Carlsson SR, Carmona-Gutierrez D, Carneiro LA, Carnevali O, Carra S, Carrier A, Carroll B, Casas C, Casas J, Cassinelli G, Castets P, Castro-Obregon S, Cavallini G, Ceccherini I, Cecconi F, Cederbaum AI, Ceña V, Cenci S, Cerella C, Cervia D, Cetrullo S, Chaachouay H, Chae HJ, Chagin AS, Chai CY, Chakrabarti G, Chamilos G, Chan EY, Chan MT, Chandra D, Chandra P, Chang CP, Chang RC, Chang TY, Chatham JC, Chatterjee S, Chauhan S, Che Y, Cheetham ME, Cheluvappa R, Chen CJ, Chen G, Chen GC, Chen G, Chen H, Chen JW, Chen JK, Chen M, Chen M, Chen P, Chen Q, Chen Q, Chen SD, Chen S, Chen SS, Chen W, Chen WJ, Chen WQ, Chen W, Chen X, Chen YH, Chen YG, Chen Y, Chen Y, Chen Y, Chen YJ, Chen YQ, Chen Y, Chen Z, Chen Z, Cheng A, Cheng CH, Cheng H, Cheong H, Cherry S, Chesney J, Cheung CH, Chevet E, Chi HC, Chi SG, Chiacchiera F, Chiang HL, Chiarelli R, Chiariello M, Chieppa M, Chin LS, Chiong M, Chiu GN, Cho DH, Cho SG, Cho WC, Cho YY, Cho YS, Choi AM, Choi EJ, Choi EK, Choi J, Choi ME, Choi SI, Chou TF, Chouaib S, Choubey D, Choubey V, Chow KC, Chowdhury K, Chu CT, Chuang TH, Chun T, Chung H, Chung T, Chung YL, Chwae YJ, Cianfanelli V, Ciarcia R, Ciechomska IA, Ciriolo MR, Cirone M, Claerhout S, Clague MJ, Clària J, Clarke PG, Clarke R, Clementi E, Cleyrat C, Cnop M, Coccia EM, Cocco T, Codogno P, Coers J, Cohen EE, Colecchia D, Coletto L, Coll NS, Colucci-Guyon E, Comincini S, Condello M, Cook KL, Coombs GH, Cooper CD, Cooper JM, Coppens I, Corasaniti MT, Corazzari M, Corbalan R, Corcelle-Termeau E, Cordero MD, Corral-Ramos C, Corti O, Cossarizza A, Costelli P, Costes S, Cotman SL, Coto-Montes A, Cottet S, Couve E, Covey LR, Cowart LA, Cox JS, Coxon FP, Coyne CB, Cragg MS, Craven RJ, Crepaldi T, Crespo JL, Criollo A, Crippa V, Cruz MT, Cuervo AM, Cuezva JM, Cui T, Cutillas PR, Czaja MJ, Czyzyk-Krzeska MF, Dagda RK, Dahmen U, Dai C, Dai W, Dai Y, Dalby KN, Dalla Valle L, Dalmasso G, D'Amelio M, Damme M, Darfeuille-Michaud A, Dargemont C, Darley-Usmar VM, Dasarathy S, Dasgupta B, Dash S, Dass CR, Davey HM, Davids LM, Dávila D, Davis RJ, Dawson TM, Dawson VL, Daza P, de Belleroche J, de Figueiredo P, de Figueiredo RC, de la Fuente J, De Martino L, De Matteis A, De Meyer GR, De Milito A, De Santi M, de Souza W, De Tata V, De Zio D, Debnath J, Dechant R, Decuypere JP, Deegan S, Dehay B, Del Bello B, Del Re DP, Delage-Mourroux R, Delbridge LM, Deldicque L, Delorme-Axford E, Deng Y, Dengjel J, Denizot M, Dent P, Der CJ, Deretic V, Derrien B, Deutsch E, Devarenne TP, Devenish RJ, Di Bartolomeo S, Di Daniele N, Di Domenico F, Di Nardo A, Di Paola S, Di Pietro A, Di Renzo L, DiAntonio A, Díaz-Araya G, Díaz-Laviada I, Diaz-Meco MT, Diaz-Nido J, Dickey CA, Dickson RC, Diederich M, Digard P, Dikic I, Dinesh-Kumar SP, Ding C, Ding WX, Ding Z, Dini L, Distler JH, Diwan A, Djavaheri-Mergny M, Dmytruk K, Dobson RC, Doetsch V, Dokladny K, Dokudovskaya S, Donadelli M, Dong XC, Dong X, Dong Z, Donohue TM Jr, Doran KS, D'Orazi G, Dorn GW 2nd, Dosenko V, Dridi S, Drucker L, Du J, Du LL, Du L, du Toit A, Dua P, Duan L, Duann P, Dubey VK, Duchen MR, Duchosal MA, Duez H, Dugail I, Dumit VI, Duncan MC, Dunlop EA, Dunn WA Jr, Dupont N, Dupuis L, Durán RV, Durcan TM, Duvezin-Caubet S, Duvvuri U, Eapen V, Ebrahimi-Fakhari D, Echard A, Eckhart L, Edelstein CL, Edinger AL, Eichinger L, Eisenberg T, Eisenberg-Lerner A, Eissa NT, El-Deiry WS, El-Khoury V, Elazar Z, Eldar-Finkelman H, Elliott CJ, Emanuele E, Emmenegger U, Engedal N, Engelbrecht AM, Engelender S, Enserink JM, Erdmann R, Erenpreisa

J, Eri R, Eriksen JL, Erman A, Escalante R, Eskelinen EL, Espert L, Esteban-Martínez L, Evans TJ, Fabri M, Fabrias G, Fabrizi C, Facchiano A, Færgeman NJ, Faggioni A, Fairlie WD, Fan C, Fan D, Fan J, Fang S, Fanto M, Fanzani A, Farkas T, Faure M, Favier FB, Fearnhead H, Federici M, Fei E, Felizardo TC, Feng H, Feng Y, Feng Y, Ferguson TA, Fernández ÁF, Fernandez-Barrena MG, Fernandez-Checa JC, Fernández-López A, Fernandez-Zapico ME, Feron O, Ferraro E, Ferreira-Halder CV, Fesus L, Feuer R, Fiesel FC, Filippi-Chiela EC, Filomeni G, Fimia GM, Fingert JH, Finkbeiner S, Finkel T, Fiorito F, Fisher PB, Flajolet M, Flamigni F, Florey O, Florio S, Floto RA, Folini M, Follo C, Fon EA, Fornai F, Fortunato F, Fraldi A, Franco R, Francois A, François A, Frankel LB, Fraser ID, Frey N, Freyssenet DG, Frezza C, Friedman SL, Frigo DE, Fu D, Fuentes JM, Fueyo J, Fujitani Y, Fujiwara Y, Fujiya M, Fukuda M, Fulda S, Fusco C, Gabryel B, Gaestel M, Gailly P, Gajewska M, Galadari S, Galili G, Galindo I, Galindo MF, Galliciotti G, Galluzzi L, Galluzzi L, Galy V, Gammoh N, Gandy S, Ganesan AK, Ganesan S, Ganley IG, Gannagé M, Gao FB, Gao F, Gao JX, García Nannig L, García Véscovi E, Garcia-Macía M, Garcia-Ruiz C, Garg AD, Garg PK, Gargini R, Gassen NC, Gatica D, Gatti E, Gavard J, Gavathiotis E, Ge L, Ge P, Ge S, Gean PW, Gelmetti V, Genazzani AA, Geng J, Genschik P, Gerner L, Gestwicki JE, Gewirtz DA, Ghavami S, Ghigo E, Ghosh D, Giammarioli AM, Giampieri F, Giampietri C, Giatromanolaki A, Gibbings DJ, Gibellini L, Gibson SB, Ginet V, Giordano A, Giorgini F, Giovannetti E, Girardin SE, Gispert S, Giuliano S, Gladson CL, Glavic A, Gleave M, Godefroy N, Gogal RM Jr, Gokulan K, Goldman GH, Goletti D, Goligorsky MS, Gomes AV, Gomes LC, Gomez H, Gomez-Manzano C, Gómez-Sánchez R, Gonçalves DA, Goncu E, Gong Q, Gongora C, Gonzalez CB, Gonzalez-Alegre P, Gonzalez-Cabo P, González-Polo RA, Goping IS, Gorbea C, Gorbunov NV, Goring DR, Gorman AM, Gorski SM, Goruppi S, Goto-Yamada S, Gotor C, Gottlieb RA, Gozes I, Gozuacik D, Graba Y, Graef M, Granato GE, Grant GD, Grant S, Gravina GL, Green DR, Greenhough A, Greenwood MT, Grimaldi B, Gros F, Grose C, Groulx JF, Gruber F, Grumati P, Grune T, Guan JL, Guan KL, Guerra B, Guillen C, Gulshan K, Gunst J, Guo C, Guo L, Guo M, Guo W, Guo XG, Gust AA, Gustafsson ÅB, Gutierrez E, Gutierrez MG, Gwak HS, Haas A, Haber JE, Hadano S, Hagedorn M, Hahn DR, Halayko AJ, Hamacher-Brady A, Hamada K, Hamai A, Hamann A, Hamasaki M, Hamer I, Hamid Q, Hammond EM, Han F, Han W, Handa JT, Hanover JA, Hansen M, Harada M, Harhaji-Trajkovic L, Harper JW, Harrath AH, Harris AL, Harris J, Hasler U, Hasselblatt P, Hasui K, Hawley RG, Hawley TS, He C, He CY, He F, He G, He RR, He XH, He YW, He YY, Heath JK, Hébert MJ, Heinzen RA, Helgason GV, Hensel M, Henske EP, Her C, Herman PK, Hernández A, Hernandez C, Hernández-Tiedra S, Hetz C, Hiesinger PR, Higaki K, Hilfiker S, Hill BG, Hill JA, Hill WD, Hino K, Hofius D, Hofman P, Höglinger GU, Höhfeld J, Holz MK, Hong Y, Hood DA, Hoozemans JJ, Hoppe T, Hsu C, Hsu CY, Hsu LC, Hu D, Hu G, Hu HM, Hu H, Hu MC, Hu YC, Hu ZW, Hua F, Hua Y, Huang C, Huang HL, Huang KH, Huang KY, Huang S, Huang S, Huang WP, Huang YR, Huang Y, Huang Y, Huber TB, Huebbe P, Huh WK, Hulmi JJ, Hur GM, Hurley JH, Husak Z, Hussain SN, Hussain S, Hwang JJ, Hwang S, Hwang TI, Ichihara A, Imai Y, Imbriano C, Inomata M, Into T, Iovane V, Iovanna JL, Iozzo RV, Ip NY, Irazoqui JE, Iribarren P, Isaka Y, Isakovic AJ, Ischiropoulos H, Isenberg JS, Ishaq M,

Ishida H, Ishii I, Ishmael JE, Isidoro C, Isobe K, Isono E, Issazadeh-Navikas S, Itahana K, Itakura E, Ivanov AI, Iyer AK, Izquierdo JM, Izumi Y, Izzo V, Jäättelä M, Jaber N, Jackson DJ, Jackson WT, Jacob TG, Jacques TS, Jagannath C, Jain A, Jana NR, Jang BK, Jani A, Janji B, Jannig PR, Jansson PJ, Jean S, Jendrach M, Jeon JH, Jessen N, Jeung EB, Jia K, Jia L, Jiang H, Jiang H, Jiang L, Jiang T, Jiang X, Jiang X, Jiang X, Jiang Y, Jiang Y, Jiménez A, Jin C, Jin H, Jin L, Jin M, Jin S, Jinwal UK, Jo EK, Johansen T, Johnson DE, Johnson GV, Johnson JD, Jonasch E, Jones C, Joosten LA, Jordan J, Joseph AM, Joseph B, Joubert AM, Ju D, Ju J, Juan HF, Juenemann K, Juhász G, Jung HS, Jung JU, Jung YK, Jungbluth H, Justice MJ, Jutten B, Kaakoush NO, Kaarniranta K, Kaasik A, Kabuta T, Kaeffer B, Kågedal K, Kahana A, Kajimura S, Kakhlon O, Kalia M, Kalvakolanu DV, Kamada Y, Kambas K, Kaminskyy VO, Kampinga HH, Kandouz M, Kang C, Kang R, Kang TC, Kanki T, Kanneganti TD, Kanno H, Kanthasamy AG, Kantorow M, Kaparakis-Liaskos M, Kapuy O, Karantza V, Karim MR, Karmakar P, Kaser A, Kaushik S, Kawula T, Kaynar AM, Ke PY, Ke ZJ, Kehrl JH, Keller KE, Kemper JK, Kenworthy AK, Kepp O, Kern A, Kesari S, Kessel D, Ketteler R, Kettelhut Ido C, Khambu B, Khan MM, Khandelwal VK, Khare S, Kiang JG, Kiger AA, Kihara A, Kim AL, Kim CH, Kim DR, Kim DH, Kim EK, Kim HY, Kim HR, Kim JS, Kim JH, Kim JC, Kim JH, Kim KW, Kim MD, Kim MM, Kim PK, Kim SW, Kim SY, Kim YS, Kim Y, Kimchi A, Kimmelman AC, Kimura T, King JS, Kirkegaard K, Kirkin V, Kirshenbaum LA, Kishi S, Kitajima Y, Kitamoto K, Kitaoka Y, Kitazato K, Kley RA, Klimecki WT, Klinkenberg M, Klucken J, Knævelsrud H, Knecht E, Knuppertz L, Ko JL, Kobayashi S, Koch JC, Koechlin-Ramonatxo C, Koenig U, Koh YH, Köhler K, Kohlwein SD, Koike M, Komatsu M, Kominami E, Kong D, Kong HJ, Konstantakou EG, Kopp BT, Korcsmaros T, Korhonen L, Korolchuk VI, Koshkina NV, Kou Y, Koukourakis MI, Koumenis C, Kovács AL, Kovács T, Kovacs WJ, Koya D, Kraft C, Krainc D, Kramer H, Kravic-Stevovic T, Krek W, Kretz-Remy C, Krick R, Krishnamurthy M, Kriston-Vizi J, Kroemer G, Kruer MC, Kruger R, Ktistakis NT, Kuchitsu K, Kuhn C, Kumar AP, Kumar A, Kumar A, Kumar D, Kumar D, Kumar R, Kumar S, Kundu M, Kung HJ, Kuno A, Kuo SH, Kuret J, Kurz T, Kwok T, Kwon TK, Kwon YT, Kyrmizi I, La Spada AR, Lafont F, Lahm T, Lakkaraju A, Lam T, Lamark T, Lancel S, Landowski TH, Lane DJ, Lane JD, Lanzi C, Lapaquette P, Lapierre LR, Laporte J, Laukkarinen J, Laurie GW, Lavandero S, Lavie L, LaVoie MJ, Law BY, Law HK, Law KB, Layfield R, Lazo PA, Le Cam L, Le Roch KG, Le Stunff H, Leardkamolkarn V, Lecuit M, Lee BH, Lee CH, Lee EF, Lee GM, Lee HJ, Lee H, Lee JK, Lee J, Lee JH, Lee JH, Lee M, Lee MS, Lee PJ, Lee SW, Lee SJ, Lee SJ, Lee SY, Lee SH, Lee SS, Lee SJ, Lee S, Lee YR, Lee YJ, Lee YH, Leeuwenburgh C, Lefort S, Legouis R, Lei J, Lei QY, Leib DA, Leibowitz G, Lekli I, Lemaire SD, Lemasters JJ, Lemberg MK, Lemoine A, Leng S, Lenz G, Lenzi P, Lerman LO, Lettieri Barbato D, Leu JI, Leung HY, Levine B, Lewis PA, Lezoualc'h F, Li C, Li F, Li FJ, Li J, Li K, Li L, Li M, Li M, Li Q, Li R, Li S, Li W, Li W, Li X, Li Y, Lian J, Liang C, Liang Q, Liao Y, Liberal J, Liberski PP, Lie P, Lieberman AP, Lim HJ, Lim KL, Lim K, Lima RT, Lin CS, Lin CF, Lin F, Lin F, Lin FC, Lin K, Lin KH, Lin PH, Lin T, Lin WW, Lin YS, Lin Y, Linden R, Lindholm D, Lindqvist LM, Lingor P, Linkermann A, Liotta LA, Lipinski MM, Lira VA, Lisanti MP, Liton PB, Liu B, Liu C, Liu CF, Liu F, Liu HJ, Liu J, Liu JJ, Liu JL, Liu K, Liu L,

Liu L, Liu Q, Liu RY, Liu S, Liu S, Liu W, Liu XD, Liu X, Liu XH, Liu X, Liu X,
Liu X, Liu Y, Liu Y, Liu Z, Liu Z, Liuzzi JP, Lizard G, Ljujic M, Lodhi IJ,
Logue SE, Lokeshwar BL, Long YC, Lonial S, Loos B, López-Otín C, López-Vicario
C, Lorente M, Lorenzi PL, Lõrincz P, Los M, Lotze MT, Lovat PE, Lu B, Lu B, Lu
J, Lu Q, Lu SM, Lu S, Lu Y, Luciano F, Luckhart S, Lucocq JM, Ludovico P, Lugea
A, Lukacs NW, Lum JJ, Lund AH, Luo H, Luo J, Luo S, Luparello C, Lyons T, Ma J,
Ma Y, Ma Y, Ma Z, Machado J, Machado-Santelli GM, Macian F, MacIntosh GC,
MacKeigan JP, Macleod KF, MacMicking JD, MacMillan-Crow LA, Madeo F, Madesh M,
Madrigal-Matute J, Maeda A, Maeda T, Maegawa G, Maellaro E, Maes H, Magariños M,
Maiese K, Maiti TK, Maiuri L, Maiuri MC, Maki CG, Malli R, Malorni W, Maloyan A,
Mami-Chouaib F, Man N, Mancias JD, Mandelkow EM, Mandell MA, Manfredi AA, Manié
SN, Manzoni C, Mao K, Mao Z, Mao ZW, Marambaud P, Marconi AM, Marelja Z, Marfe
G, Margeta M, Margittai E, Mari M, Mariani FV, Marin C, Marinelli S, Mariño G,
Markovic I, Marquez R, Martelli AM, Martens S, Martin KR, Martin SJ, Martin S,
Martin-Acebes MA, Martín-Sanz P, Martinand-Mari C, Martinet W, Martinez J,
Martinez-Lopez N, Martinez-Outschoorn U, Martínez-Velázquez M, Martinez-Vicente
M, Martins WK, Mashima H, Mastrianni JA, Matarese G, Matarrese P, Mateo R,
Matoba S, Matsumoto N, Matsushita T, Matsuura A, Matsuzawa T, Mattson MP, Matus
S, Maugeri N, Mauvezin C, Mayer A, Maysinger D, Mazzolini GD, McBrayer MK,
McCall K, McCormick C, McInerney GM, McIver SC, McKenna S, McMahon JJ, McNeish
IA, Mechta-Grigoriou F, Medema JP, Medina DL, Megyeri K, Mehrpour M, Mehta JL,
Mei Y, Meier UC, Meijer AJ, Meléndez A, Melino G, Melino S, de Melo EJ, Mena MA,
Meneghini MD, Menendez JA, Menezes R, Meng L, Meng LH, Meng S, Menghini R, Menko
AS, Menna-Barreto RF, Menon MB, Meraz-Ríos MA, Merla G, Merlini L, Merlot AM,
Meryk A, Meschini S, Meyer JN, Mi MT, Miao CY, Micale L, Michaeli S, Michiels C,
Migliaccio AR, Mihailidou AS, Mijaljica D, Mikoshiba K, Milan E, Miller-Fleming
L, Mills GB, Mills IG, Minakaki G, Minassian BA, Ming XF, Minibayeva F, Minina
EA, Mintern JD, Minucci S, Miranda-Vizuete A, Mitchell CH, Miyamoto S, Miyazawa
K, Mizushima N, Mnich K, Mograbi B, Mohseni S, Moita LF, Molinari M, Molinari M,
Møller AB, Mollereau B, Mollinedo F, Mongillo M, Monick MM, Montagnaro S,
Montell C, Moore DJ, Moore MN, Mora-Rodriguez R, Moreira PI, Morel E, Morelli
MB, Moreno S, Morgan MJ, Moris A, Moriyasu Y, Morrison JL, Morrison LA, Morselli
E, Moscat J, Moseley PL, Mostowy S, Motori E, Mottet D, Mottram JC, Moussa CE,
Mpakou VE, Mukhtar H, Mulcahy Levy JM, Muller S, Muñoz-Moreno R, Muñoz-Pinedo C,
Münz C, Murphy ME, Murray JT, Murthy A, Mysorekar IU, Nabi IR, Nabissi M, Nader
GA, Nagahara Y, Nagai Y, Nagata K, Nagelkerke A, Nagy P, Naidu SR, Nair S,
Nakano H, Nakatogawa H, Nanjundan M, Napolitano G, Naqvi NI, Nardacci R,
Narendra DP, Narita M, Nascimbeni AC, Natarajan R, Navegantes LC, Nawrocki ST,
Nazarko TY, Nazarko VY, Neill T, Neri LM, Netea MG, Netea-Maier RT, Neves BM,
Ney PA, Nezis IP, Nguyen HT, Nguyen HP, Nicot AS, Nilsen H, Nilsson P, Nishimura
M, Nishino I, Niso-Santano M, Niu H, Nixon RA, Njar VC, Noda T, Noegel AA, Nolte
EM, Norberg E, Norga KK, Noureini SK, Notomi S, Notterpek L, Nowikovsky K,
Nukina N, Nürnberger T, O'Donnell VB, O'Donovan T, O'Dwyer PJ, Oehme I, Oeste
CL, Ogawa M, Ogretmen B, Ogura Y, Oh YJ, Ohmuraya M, Ohshima T, Ojha R, Okamoto
K, Okazaki T, Oliver FJ, Ollinger K, Olsson S, Orban DP, Ordonez P, Orhon I,

Orosz L, O'Rourke EJ, Orozco H, Ortega AL, Ortona E, Osellame LD, Oshima J, Oshima S, Osiewacz HD, Otomo T, Otsu K, Ou JH, Outeiro TF, Ouyang DY, Ouyang H, Overholtzer M, Ozbun MA, Ozdinler PH, Ozpolat B, Pacelli C, Paganetti P, Page G, Pages G, Pagnini U, Pajak B, Pak SC, Pakos-Zebrucka K, Pakpour N, Palková Z, Palladino F, Pallauf K, Pallet N, Palmieri M, Paludan SR, Palumbo C, Palumbo S, Pampliega O, Pan H, Pan W, Panaretakis T, Pandey A, Pantazopoulou A, Papackova Z, Papademetrio DL, Papassideri I, Papini A, Parajuli N, Pardo J, Parekh VV, Parenti G, Park JI, Park J, Park OK, Parker R, Parlato R, Parys JB, Parzych KR, Pasquet JM, Pasquier B, Pasumarthi KB, Patschan D, Patterson C, Pattingre S, Pattison S, Pause A, Pavenstädt H, Pavone F, Pedrozo Z, Peña FJ, Peñalva MA, Pende M, Peng J, Penna F, Penninger JM, Pensalfini A, Pepe S, Pereira GJ, Pereira PC, Pérez-de la Cruz V, Pérez-Pérez ME, Pérez-Rodríguez D, Pérez-Sala D, Perier C, Perl A, Perlmutter DH, Perrotta I, Pervaiz S, Pesonen M, Pessin JE, Peters GJ, Petersen M, Petrache I, Petrof BJ, Petrovski G, Phang JM, Piacentini M, Pierdominici M, Pierre P, Pierrefite-Carle V, Pietrocola F, Pimentel-Muiños FX, Pinar M, Pineda B, Pinkas-Kramarski R, Pinti M, Pinton P, Piperdi B, Piret JM, Platanias LC, Platta HW, Plowey ED, Pöggeler S, Poirot M, Polčic P, Poletti A, Poon AH, Popelka H, Popova B, Poprawa I, Poulose SM, Poulton J, Powers SK, Powers T, Pozuelo-Rubio M, Prak K, Prange R, Prescott M, Priault M, Prince S, Proia RL, Proikas-Cezanne T, Prokisch H, Promponas VJ, Przyklenk K, Puertollano R, Pugazhenthi S, Puglielli L, Pujol A, Puyal J, Pyeon D, Qi X, Qian WB, Qin ZH, Qiu Y, Qu Z, Quadrilatero J, Quinn F, Raben N, Rabinowich H, Radogna F, Ragusa MJ, Rahmani M, Raina K, Ramanadham S, Ramesh R, Rami A, Randall-Demllo S, Randow F, Rao H, Rao VA, Rasmussen BB, Rasse TM, Ratovitski EA, Rautou PE, Ray SK, Razani B, Reed BH, Reggiori F, Rehm M, Reichert AS, Rein T, Reiner DJ, Reits E, Ren J, Ren X, Renna M, Reusch JE, Revuelta JL, Reyes L, Rezaie AR, Richards RI, Richardson DR, Richetta C, Riehle MA, Rihn BH, Rikihisa Y, Riley BE, Rimbach G, Rippo MR, Ritis K, Rizzi F, Rizzo E, Roach PJ, Robbins J, Roberge M, Roca G, Roccheri MC, Rocha S, Rodrigues CMP, Rodríguez CI, de Cordoba SR, Rodriguez-Muela N, Roelofs J, Rogov VV, Rohn TT, Rohrer B, Romanelli D, Romani L, Romano PS, Roncero MI, Rosa JL, Rosello A, Rosen KV, Rosenstiel P, Rost-Roszkowska M, Roth KA, Roué G, Rouis M, Rouschop KM, Ruan DT, Ruano D, Rubinsztein DC, Rucker EB 3rd, Rudich A, Rudolf E, Rudolf R, Ruegg MA, Ruiz-Roldan C, Ruparelia AA, Rusmini P, Russ DW, Russo GL, Russo G, Russo R, Rusten TE, Ryabovol V, Ryan KM, Ryter SW, Sabatini DM, Sacher M, Sachse C, Sack MN, Sadoshima J, Saftig P, Sagi-Eisenberg R, Sahni S, Saikumar P, Saito T, Saitoh T, Sakakura K, Sakoh-Nakatogawa M, Sakuraba Y, Salazar-Roa M, Salomoni P, Saluja AK, Salvaterra PM, Salvioli R, Samali A, Sanchez AM, Sánchez-Alcázar JA, Sanchez-Prieto R, Sandri M, Sanjuan MA, Santaguida S, Santambrogio L, Santoni G, Dos Santos CN, Saran S, Sardiello M, Sargent G, Sarkar P, Sarkar S, Sarrias MR, Sarwal MM, Sasakawa C, Sasaki M, Sass M, Sato K, Sato M, Satriano J, Savaraj N, Saveljeva S, Schaefer L, Schaible UE, Scharl M, Schatzl HM, Schekman R, Scheper W, Schiavi A, Schipper HM, Schmeisser H, Schmidt J, Schmitz I, Schneider BE, Schneider EM, Schneider JL, Schon EA, Schönenberger MJ, Schönthal AH, Schorderet DF, Schröder B, Schuck S, Schulze RJ, Schwarten M, Schwarz TL, Sciarretta S, Scotto K, Scovassi AI,

Screaton RA, Screen M, Seca H, Sedej S, Segatori L, Segev N, Seglen PO, Seguí-Simarro JM, Segura-Aguilar J, Seki E, Sell C, Seiliez I, Semenkovich CF, Semenza GL, Sen U, Serra AL, Serrano-Puebla A, Sesaki H, Setoguchi T, Settembre C, Shacka JJ, Shajahan-Haq AN, Shapiro IM, Sharma S, She H, Shen CK, Shen CC, Shen HM, Shen S, Shen W, Sheng R, Sheng X, Sheng ZH, Shepherd TG, Shi J, Shi Q, Shi Q, Shi Y, Shibutani S, Shibuya K, Shidoji Y, Shieh JJ, Shih CM, Shimada Y, Shimizu S, Shin DW, Shinohara ML, Shintani M, Shintani T, Shioi T, Shirabe K, Shiri-Sverdlov R, Shirihai O, Shore GC, Shu CW, Shukla D, Sibirny AA, Sica V, Sigurdson CJ, Sigurdsson EM, Sijwali PS, Sikorska B, Silveira WA, Silvente-Poirot S, Silverman GA, Simak J, Simmet T, Simon AK, Simon HU, Simone C, Simons M, Simonsen A, Singh R, Singh SV, Singh SK, Sinha D, Sinha S, Sinicrope FA, Sirko A, Sirohi K, Sishi BJ, Sittler A, Siu PM, Sivridis E, Skwarska A, Slack R, Slaninová I, Slavov N, Smaili SS, Smalley KS, Smith DR, Soenen SJ, Soleimanpour SA, Solhaug A, Somasundaram K, Son JH, Sonawane A, Song C, Song F, Song HK, Song JX, Song W, Soo KY, Sood AK, Soong TW, Soontornniyomkij V, Sorice M, Sotgia F, Soto-Pantoja DR, Sotthibundhu A, Sousa MJ, Spaink HP, Span PN, Spang A, Sparks JD, Speck PG, Spector SA, Spies CD, Springer W, Clair DS, Stacchiotti A, Staels B, Stang MT, Starczynowski DT, Starokadomskyy P, Steegborn C, Steele JW, Stefanis L, Steffan J, Stellrecht CM, Stenmark H, Stepkowski TM, Stern ST, Stevens C, Stockwell BR, Stoka V, Storchova Z, Stork B, Stratoulias V, Stravopodis DJ, Strnad P, Strohecker AM, Ström AL, Stromhaug P, Stulik J, Su YX, Su Z, Subauste CS, Subramaniam S, Sue CM, Suh SW, Sui X, Sukseree S, Sulzer D, Sun FL, Sun J, Sun J, Sun SY, Sun Y, Sun Y, Sun Y, Sundaramoorthy V, Sung J, Suzuki H, Suzuki K, Suzuki N, Suzuki T, Suzuki YJ, Swanson MS, Swanton C, Swärd K, Swarup G, Sweeney ST, Sylvester PW, Szatmari Z, Szegezdi E, Szlosarek PW, Taegtmeyer H, Tafani M, Taillebourg E, Tait SW, Takacs-Vellai K, Takahashi Y, Takáts S, Takemura G, Takigawa N, Talbot NJ, Tamagno E, Tamburini J, Tan CP, Tan L, Tan ML, Tan M, Tan YJ, Tanaka K, Tanaka M, Tang D, Tang D, Tang G, Tanida I, Tanji K, Tannous BA, Tapia JA, Tasset-Cuevas I, Tatar M, Tavassoly I, Tavernarakis N, Taylor A, Taylor GS, Taylor GA, Taylor JP, Taylor MJ, Tchetina EV, Tee AR, Teixeira-Clerc F, Telang S, Tencomnao T, Teng BB, Teng RJ, Terro F, Tettamanti G, Theiss AL, Theron AE, Thomas KJ, Thomé MP, Thomes PG, Thorburn A, Thorner J, Thum T, Thumm M, Thurston TL, Tian L, Till A, Ting JP, Titorenko VI, Toker L, Toldo S, Tooze SA, Topisirovic I, Torgersen ML, Torosantucci L, Torriglia A, Torrisi MR, Tournier C, Towns R, Trajkovic V, Travassos LH, Triola G, Tripathi DN, Trisciuoglio D, Troncoso R, Trougakos IP, Truttmann AC, Tsai KJ, Tschan MP, Tseng YH, Tsukuba T, Tsung A, Tsvetkov AS, Tu S, Tuan HY, Tucci M, Tumbarello DA, Turk B, Turk V, Turner RF, Tveita AA, Tyagi SC, Ubukata M, Uchiyama Y, Udelnow A, Ueno T, Umekawa M, Umemiya-Shirafuji R, Underwood BR, Ungermann C, Ureshino RP, Ushioda R, Uversky VN, Uzcátegui NL, Vaccari T, Vaccaro MI, Váchová L, Vakifahmetoglu-Norberg H, Valdor R, Valente EM, Vallette F, Valverde AM, Van den Berghe G, Van Den Bosch L, van den Brink GR, van der Goot FG, van der Klei IJ, van der Laan LJ, van Doorn WG, van Egmond M, van Golen KL, Van Kaer L, van Lookeren Campagne M, Vandenabeele P, Vandenberghe W, Vanhorebeek I, Varela-Nieto I, Vasconcelos MH, Vasko R, Vavvas DG, Vega-Naredo

I, Velasco G, Velentzas AD, Velentzas PD, Vellai T, Vellenga E, Vendelbo MH, Venkatachalam K, Ventura N, Ventura S, Veras PS, Verdier M, Vertessy BG, Viale A, Vidal M, Vieira HL, Vierstra RD, Vigneswaran N, Vij N, Vila M, Villar M, Villar VH, Villarroya J, Vindis C, Viola G, Viscomi MT, Vitale G, Vogl DT, Voitsekhovskaja OV, von Haefen C, von Schwarzenberg K, Voth DE, Vouret-Craviari V, Vuori K, Vyas JM, Waeber C, Walker CL, Walker MJ, Walter J, Wan L, Wan X, Wang B, Wang C, Wang CY, Wang C, Wang C, Wang C, Wang D, Wang F, Wang F, Wang G, Wang HJ, Wang H, Wang HG, Wang H, Wang HD, Wang J, Wang J, Wang M, Wang MQ, Wang PY, Wang P, Wang RC, Wang S, Wang TF, Wang X, Wang XJ, Wang XW, Wang X, Wang X, Wang Y, Wang Y, Wang Y, Wang YJ, Wang Y, Wang Y, Wang YT, Wang Y, Wang ZN, Wappner P, Ward C, Ward DM, Warnes G, Watada H, Watanabe Y, Watase K, Weaver TE, Weekes CD, Wei J, Weide T, Weihl CC, Weindl G, Weis SN, Wen L, Wen X, Wen Y, Westermann B, Weyand CM, White AR, White E, Whitton JL, Whitworth AJ, Wiels J, Wild F, Wildenberg ME, Wileman T, Wilkinson DS, Wilkinson S, Willbold D, Williams C, Williams K, Williamson PR, Winklhofer KF, Witkin SS, Wohlgemuth SE, Wollert T, Wolvetang EJ, Wong E, Wong GW, Wong RW, Wong VK, Woodcock EA, Wright KL, Wu C, Wu D, Wu GS, Wu J, Wu J, Wu M, Wu M, Wu S, Wu WK, Wu Y, Wu Z, Xavier CP, Xavier RJ, Xia GX, Xia T, Xia W, Xia Y, Xiao H, Xiao J, Xiao S, Xiao W, Xie CM, Xie Z, Xie Z, Xilouri M, Xiong Y, Xu C, Xu C, Xu F, Xu H, Xu H, Xu J, Xu J, Xu J, Xu L, Xu X, Xu Y, Xu Y, Xu ZX, Xu Z, Xue Y, Yamada T, Yamamoto A, Yamanaka K, Yamashina S, Yamashiro S, Yan B, Yan B, Yan X, Yan Z, Yanagi Y, Yang DS, Yang JM, Yang L, Yang M, Yang PM, Yang P, Yang Q, Yang W, Yang WY, Yang X, Yang Y, Yang Y, Yang Z, Yang Z, Yao MC, Yao PJ, Yao X, Yao Z, Yao Z, Yasui LS, Ye M, Yedvobnick B, Yeganeh B, Yeh ES, Yeyati PL, Yi F, Yi L, Yin XM, Yip CK, Yoo YM, Yoo YH, Yoon SY, Yoshida K, Yoshimori T, Young KH, Yu H, Yu JJ, Yu JT, Yu J, Yu L, Yu WH, Yu XF, Yu Z, Yuan J, Yuan ZM, Yue BY, Yue J, Yue Z, Zacks DN, Zacksenhaus E, Zaffaroni N, Zaglia T, Zakeri Z, Zecchini V, Zeng J, Zeng M, Zeng Q, Zervos AS, Zhang DD, Zhang F, Zhang G, Zhang GC, Zhang H, Zhang H, Zhang H, Zhang H, Zhang J, Zhang J, Zhang J, Zhang J, Zhang JP, Zhang L, Zhang L, Zhang L, Zhang L, Zhang MY, Zhang X, Zhang XD, Zhang Y, Zhang Y, Zhang Y, Zhang Y, Zhang Y, Zhao M, Zhao WL, Zhao X, Zhao YG, Zhao Y, Zhao Y, Zhao YX, Zhao Z, Zhao ZJ, Zheng D, Zheng XL, Zheng X, Zhivotovsky B, Zhong Q, Zhou GZ, Zhou G, Zhou H, Zhou SF, Zhou XJ, Zhu H, Zhu H, Zhu WG, Zhu W, Zhu XF, Zhu Y, Zhuang SM, Zhuang X, Ziparo E, Zois CE, Zoladek T, Zong WX, Zorzano A, Zughaier SM. Guidelines for the use and interpretation of assays for monitoring autophagy (3rd edition). Autophagy. 2016;12(1):1-222. doi: 10.1080/15548627.2015.1100356. Erratum in: Autophagy. 2016;12(2):443. Selliez, Iban [corrected to Seiliez, Iban]. PMID: 26799652; PMCID: PMC4835977.

233: Dhaka N, Samanta J, Kochhar S, Kalra N, Appasani S, Manrai M, Kochhar R. Pancreatic fluid collections: What is the ideal imaging technique? World J Gastroenterol. 2015 Dec 28;21(48):13403-10. doi: 10.3748/wjg.v21.i48.13403. PMID: 26730150; PMCID: PMC4690168.

234: Pathuri G, Hedrick AF, Awasthi V, Cowley BD Jr, Gali H. Evaluation of

[18F]PFH PET renography to predict future disease progression in a rat model of autosomal dominant polycystic kidney disease. Nucl Med Biol. 2016 Jan;43(1):1-5. doi: 10.1016/j.nucmedbio.2015.10.002. Epub 2015 Oct 22. PMID: 26702780.

235: Scholtens AM, Verberne HJ, Budde RP, Lam MG. Additional Heparin Preadministration Improves Cardiac Glucose Metabolism Suppression over Low-Carbohydrate Diet Alone in ^{18}F-FDG PET Imaging. J Nucl Med. 2016 Apr;57(4):568-73. doi: 10.2967/jnumed.115.166884. Epub 2015 Dec 10. PMID: 26659348.

236: Fiogbe AA, Liistro G, Hoton D, Pieters T. Infection à Mycobacterium avium simulant un adénocarcinome pulmonaire : un potentiel piège diagnostique [Mycobacterium avium tumoral infection mimicking a lung adenocarcinoma: A potential diagnostic pitfall]. Rev Pneumol Clin. 2016 Apr;72(2):147-51. French. doi: 10.1016/j.pneumo.2015.08.003. Epub 2015 Dec 2. PMID: 26651929.

237: Castellano CA, Baillargeon JP, Nugent S, Tremblay S, Fortier M, Imbeault H, Duval J, Cunnane SC. Regional Brain Glucose Hypometabolism in Young Women with Polycystic Ovary Syndrome: Possible Link to Mild Insulin Resistance. PLoS One. 2015 Dec 9;10(12):e0144116. doi: 10.1371/journal.pone.0144116. PMID: 26650926; PMCID: PMC4674147.

238: Claimon A, Bang JI, Cheon GJ, Kim EE, Lee DS. Malignant Peritoneal Mesothelioma Masquerades as Peritoneal Metastasis on (18)F-FDG PET/CT Scans; a Rare Diagnosis that Should Not Be Missed. Nucl Med Mol Imaging. 2015 Dec;49(4):325-8. doi: 10.1007/s13139-015-0360-2. Epub 2015 Aug 18. PMID: 26550054; PMCID: PMC4630325.

239: Park JY, Kim SG, Kim JS, Jung HC. Bone marrow involvement is rare in superficial gastric mucosa-associated lymphoid tissue lymphoma. Dig Liver Dis. 2016 Jan;48(1):81-6. doi: 10.1016/j.dld.2015.10.008. Epub 2015 Oct 23. PMID: 26548745.

240: Guler S, Cimen S, Hurton S, Molinari M. Diagnosis and Treatment Modalities of Symptomatic Polycystic Kidney Disease. In: Li X, editor. Polycystic Kidney Disease [Internet]. Brisbane (AU): Codon Publications; 2015 Nov. Chapter 4. PMID: 27512786.

241: Bobot M, Ghez C, Gondouin B, Sallée M, Fournier PE, Burtey S, Legris T, Dussol B, Berland Y, Souteyrand P, Tessonnier L, Cammilleri S, Jourde-Chiche N. Diagnostic performance of [(18)F]fluorodeoxyglucose positron emission tomography-computed tomography in cyst infection in patients with autosomal dominant polycystic kidney disease. Clin Microbiol Infect. 2016 Jan;22(1):71-77. doi: 10.1016/j.cmi.2015.09.024. Epub 2015 Oct 8. PMID: 26454062.

242: Hamauchi S, Yokota T, Onozawa Y, Ogawa H, Onoe T, Kamijo T, Iida Y, Nishimura T, Onitsuka T, Yasui H. Safety and efficacy of concurrent carboplatin plus radiotherapy for locally advanced head and neck cancer patients ineligible for treatment with cisplatin. Jpn J Clin Oncol. 2015 Dec;45(12):1116-21. doi: 10.1093/jjco/hyv142. Epub 2015 Sep 29. PMID: 26423341.

243: Chelazzi C, Pettini E, Villa G, De Gaudio AR. Epidemiology, associated factors and outcomes of ICU-acquired infections caused by Gram-negative bacteria in critically ill patients: an observational, retrospective study. BMC Anesthesiol. 2015 Sep 21;15:125. doi: 10.1186/s12871-015-0106-9. PMID: 26392077; PMCID: PMC4578757.

244: Adler Y, Charron P, Imazio M, Badano L, Barón-Esquivias G, Bogaert J, Brucato A, Gueret P, Klingel K, Lionis C, Maisch B, Mayosi B, Pavie A, Ristic AD, Sabaté Tenas M, Seferovic P, Swedberg K, Tomkowski W; ESC Scientific Document Group. 2015 ESC Guidelines for the diagnosis and management of pericardial diseases: The Task Force for the Diagnosis and Management of Pericardial Diseases of the European Society of Cardiology (ESC)Endorsed by: The European Association for Cardio-Thoracic Surgery (EACTS). Eur Heart J. 2015 Nov 7;36(42):2921-2964. doi: 10.1093/eurheartj/ehv318. Epub 2015 Aug 29. PMID: 26320112; PMCID: PMC7539677.

245: Hizem A, M'rad S, Oudni-M'rad M, Mestiri S, Hammedi F, Mezhoud H, Zakhama A, Mokni M, Babba H. Molecular genotyping of Echinococcus granulosus using formalin-fixed paraffin-embedded preparations from human isolates in unusual tissue sites. J Helminthol. 2016 Jul;90(4):417-21. doi: 10.1017/S0022149X15000516. Epub 2015 Jul 20. PMID: 26190231.

246: Birendra KC, Afzal MZ, Wentland KA, Hashmi H, Singh S, Ivan E, Lakhani N. Spontaneous Regression of Refractory Diffuse Large B-Cell Lymphoma with Improvement in Immune Status with ART in a Patient with HIV: A Case Report and Literature Review. Am J Case Rep. 2015 Jun 5;16:347-52. doi: 10.12659/AJCR.892883. PMID: 26046822; PMCID: PMC4467606.

247: Mihailovič J, Matovina E, Nikoletič K. 18F-fluorideoxyglucose positron emission tomography/computed tomography imaging: artifacts and pitfalls. Med Pregl. 2015 Jan-Feb;68(1-2):41-8. doi: 10.2298/mpns1502041m. PMID: 26012243.

248: Lantinga MA, de Sévaux RG, Drenth JP. 18F-FDG PET/CT during diagnosis and follow-up of recurrent hepatic cyst infection in autosomal dominant polycystic kidney disease. Clin Nephrol. 2015 Jul;84(1):61-4. doi: 10.5414/CN108495. PMID: 25881686.

249: Sioka C, Assimakopoulos A, Fotopoulos A. The diagnostic role of (18)F fluorodeoxyglucose positron emission tomography in patients with fever of

unknown origin. Eur J Clin Invest. 2015 Jun;45(6):601-8. doi: 10.1111/eci.12439. Epub 2015 Apr 24. PMID: 25823953.

250: Walczewska M, Mocarska A, Burdan F, Janczarek M, Żelazowska-Cieślińska I, Starosławska E. Diagnostyka obrazowa zmian łagodnych w jajnikach [Diagnosis of benign ovarian lesions using imaging techniques]. Pol Merkur Lekarski. 2015 Jan;38(223):55-60. Polish. PMID: 25763591.

251: Li HX, Lu XJ, Li CH, Chen J. Molecular characterization of the liver-expressed antimicrobial peptide 2 (LEAP-2) in a teleost fish, Plecoglossus altivelis: antimicrobial activity and molecular mechanism. Mol Immunol. 2015 Jun;65(2):406-15. doi: 10.1016/j.molimm.2015.02.022. Epub 2015 Mar 6. PMID: 25749706.

252: Pirson Y, Kanaan N. Complications infectieuses associées à la polykystose rénale autosomique dominante [Infectious complications in autosomal dominant polycystic kidney disease]. Nephrol Ther. 2015 Apr;11(2):73-7. French. doi: 10.1016/j.nephro.2014.11.008. Epub 2015 Feb 27. PMID: 25733396.

253: Paschali AN, Georgakopoulos AT, Pianou NK, Anagnostopoulos CD. (18)F-fluorodeoxyglucose positron emission tomography/computed tomography in infected polycystic kidney disease. World J Nucl Med. 2015 Jan-Apr;14(1):57-9. doi: 10.4103/1450-1147.150553. PMID: 25709548; PMCID: PMC4337011.

254: Liang R, Wang Z, Chen XQ, Bai QX. Treatment of plasmablastic lymphoma with multiple organ involvement. Singapore Med J. 2014 Dec;55(12):e194-7. doi: 10.11622/smedj.2014184. PMID: 25630325; PMCID: PMC4292009.

255: Tang J, Abbas J, Hoetzl K, Allison D, Osman M, Williams M, Zelenock GB. Ligation of superior mesenteric vein and portal to splenic vein anastomosis after superior mesenteric-portal vein confluence resection during pancreaticoduodenectomy - Case report. Ann Med Surg (Lond). 2014 Oct 2;3(4):137-40. doi: 10.1016/j.amsu.2014.08.001. PMID: 25568802; PMCID: PMC4284446.

256: Wijnands TF, Lantinga MA, Drenth JP. Hepatic cyst infection following aspiration sclerotherapy: a case series. J Gastrointestin Liver Dis. 2014 Dec;23(4):441-4. doi: 10.15403/jgld.2014.1121.234.hcy. PMID: 25532005.

257: Krol JJ, Krol VV, Dawkins A, Ganesh HS. Case 213: primary splenic angiosarcoma. Radiology. 2015 Jan;274(1):298-303. doi: 10.1148/radiol.14110919. PMID: 25531483.

258: Kytö V, Sipilä J, Rautava P. Gender and in-hospital mortality of ST-segment elevation myocardial infarction (from a multihospital nationwide registry study

of 31,689 patients). Am J Cardiol. 2015 Feb 1;115(3):303-6. doi: 10.1016/j.amjcard.2014.11.001. Epub 2014 Nov 13. PMID: 25488357.

259: Hofer A, Kratochwill H, Pentsch A, Gabriel M. Detection of necrosis of the gastric fundus after blunt abdominal trauma by PET-CT. Wien Klin Wochenschr. 2015 Feb;127(3-4):89-91. doi: 10.1007/s00508-014-0648-8. Epub 2014 Nov 25. PMID: 25421368.

260: Sculier D, Doco-Lecompte T, Rougemont M, Calmy A. Haemophagocytic syndrome and elevated EBV load as initial manifestation of Hodgkin lymphoma in a HIV patient: case report and review of the literature. J Int AIDS Soc. 2014 Nov 2;17(4 Suppl 3):19650. doi: 10.7448/IAS.17.4.19650. PMID: 25394154; PMCID: PMC4224866.

261: Min BH, Park JY, Kim ER, Min YW, Lee JH, Rhee PL, Rhee JC, Kim JJ. Limited role of bone marrow aspiration and biopsy in the initial staging work-up of gastric mucosa-associated lymphoid tissue lymphoma in Korea. Gut Liver. 2014 Nov;8(6):637-42. doi: 10.5009/gnl13284. Epub 2014 Nov 15. PMID: 25368752; PMCID: PMC4215450.

262: Niu JH, Yang H, Zhang Q, Zhu CY, Zhu HY, Yao ZL, Yu L, Jing Y. [Efficacy of liver transplantation for acute hepatic failure caused by reactivation of hepatitis B virus infection after chemotherapy and radiotherapy for diffuse large B-cell lymphoma]. Zhongguo Shi Yan Xue Ye Xue Za Zhi. 2014 Aug;22(4):1005-11. Chinese. doi: 10.7534/j.issn.1009-2137.2014.04.022. PMID: 25130818.

263: Lantinga MA, Drenth JP, Gevers TJ. Diagnostic criteria in renal and hepatic cyst infection. Nephrol Dial Transplant. 2015 May;30(5):744-51. doi: 10.1093/ndt/gfu227. Epub 2014 Jun 20. PMID: 24950937.

264: Balbo BE, Sapienza MT, Ono CR, Jayanthi SK, Dettoni JB, Castro I, Onuchic LF. Cyst infection in hospital-admitted autosomal dominant polycystic kidney disease patients is predominantly multifocal and associated with kidney and liver volume. Braz J Med Biol Res. 2014 Jul;47(7):584-93. doi: 10.1590/1414-431x20143584. Epub 2014 Jun 13. PMID: 24919173; PMCID: PMC4123838.

265: Chirindel A, Alluri KC, Tahari AK, Chaudhry M, Wahl RL, Lodge MA, Subramaniam RM. Liver standardized uptake value corrected for lean body mass at FDG PET/CT: effect of FDG uptake time. Clin Nucl Med. 2015 Jan;40(1):e17-22. doi: 10.1097/RLU.0000000000000446. PMID: 24873794; PMCID: PMC5413863.

266: Tammana VS, Begum R, Oneal P, Karpurapu H, Muley A, Yeruva SL, Dunmore-Griffith J, Mekonen E, Hasan N. A novel use of early radiation therapy in the treatment of hyperbilirubinemia in a patient with primary hepatic lymphoma and

chronic hepatitis C. Case Rep Gastrointest Med. 2014;2014:724256. doi: 10.1155/2014/724256. Epub 2014 Apr 29. PMID: 24872902; PMCID: PMC4020534.

267: Wan HL, Hui PW, Li HW, Ng EH. Obstetric outcomes in women with polycystic ovary syndrome and isolated polycystic ovaries undergoing in vitro fertilization: a retrospective cohort analysis. J Matern Fetal Neonatal Med. 2015 Mar;28(4):475-8. doi: 10.3109/14767058.2014.921673. Epub 2014 May 29. PMID: 24803007.

268: Tenley N, Corn DJ, Yuan L, Lee Z. The effect of fasting on PET Imaging of Hepatocellular Carcinoma. J Cancer Ther. 2013 Apr;4(2):561-567. doi: 10.4236/jct.2013.42071. PMID: 24683497; PMCID: PMC3966532.

269: Jung JS, Lee SM, Kim HJ, Jang SH, Lee JW. A case of septic pulmonary embolism associated with renal abscess mimicking pulmonary metastases of renal malignancy. Ann Nucl Med. 2014 May;28(4):381-5. doi: 10.1007/s12149-014-0811-3. Epub 2014 Jan 31. PMID: 24481822.

270: Rahbari-Oskoui F, Mittal A, Mittal P, Chapman A. Renal relevant radiology: radiologic imaging in autosomal dominant polycystic kidney disease. Clin J Am Soc Nephrol. 2014 Feb;9(2):406-15. doi: 10.2215/CJN.08940813. Epub 2013 Dec 26. PMID: 24370765; PMCID: PMC3913246.

271: Mansfield SA, Stawicki SP, Forbes RC, Papadimos TJ, Lindsey DE. Acute upper gastrointestinal bleeding secondary to Kaposi sarcoma as initial presentation of HIV infection. J Gastrointestin Liver Dis. 2013 Dec;22(4):441-5. PMID: 24369327; PMCID: PMC4097021.

272: Buchler T, Homolka J, Fencl P, Rosova B, Hytych V, Abrahamova J. Nontuberculous mycobacterial infection after therapy with temsirolimus for metastatic renal cell carcinoma. Tumori. 2013 Jul-Aug;99(4):e159-63. doi: 10.1177/030089161309900425. PMID: 24326853.

273: Simpson VR, Hargreaves J, Butler HM, Davison NJ, Everest DJ. Causes of mortality and pathological lesions observed post-mortem in red squirrels (Sciurus vulgaris) in Great Britain. BMC Vet Res. 2013 Nov 16;9:229. doi: 10.1186/1746-6148-9-229. PMID: 24238087; PMCID: PMC4225685.

274: Runge VM. Current technological advances in magnetic resonance with critical impact for clinical diagnosis and therapy. Invest Radiol. 2013 Dec;48(12):869-77. doi: 10.1097/01.rli.0000434380.71793.d3. PMID: 24126386.

275: Naboush A, Farhat F, Nasser SM, Kamar FG. Bifocal Presentation of Primary Testicular Extranasal NK/T-Cell Lymphoma: A Case Report and Review of the Literature. Case Rep Oncol Med. 2013;2013:267389. doi: 10.1155/2013/267389. Epub

2013 Aug 12. PMID: 23997969; PMCID: PMC3753765.

276: Dziadzio M, Chee R, McNamara C, Deheragoda M, Wagner T, Seneviratne SL. EBV-driven diffuse large B-cell lymphoma confined to the liver in a patient with a history of idiopathic CD4 lymphocytopenia. BMJ Case Rep. 2013 Aug 21;2013:bcr2013009721. doi: 10.1136/bcr-2013-009721. PMID: 23966455; PMCID: PMC3761448.

277: Mucha K, Foroncewicz B, Orłowski T, Religioni J, Bobek-Billewicz B, Jarząb B, Raczyńska J, Krawczyk M, Pączek L. Atypical presentation of invasive pulmonary aspergillosis in a liver transplant recipient. Ann Transplant. 2013 May 23;18:238-42. doi: 10.12659/AOT.883921. PMID: 23792526.

278: Aydin M, Akkoyunlu ME, Yakar F, Ergul N, Sezer M. Intense FDG uptake on PET/CT in the upper and lower respiratory system indicative of Wegener's granulomatosis. Rev Esp Med Nucl Imagen Mol. 2014 Jan-Feb;33(1):32-5. doi: 10.1016/j.remn.2013.03.003. Epub 2013 May 14. PMID: 23680478.

279: Marsh CA, Berent-Spillson A, Love T, Persad CC, Pop-Busui R, Zubieta JK, Smith YR. Functional neuroimaging of emotional processing in women with polycystic ovary syndrome: a case-control pilot study. Fertil Steril. 2013 Jul;100(1):200-7.e1. doi: 10.1016/j.fertnstert.2013.02.054. Epub 2013 Apr 1. PMID: 23557757; PMCID: PMC3900232.

280: Hamada T, Kakizaki S, Koiso H, Irisawa H, Nobusawa S, Mori M. Primary hepatic mucosa-associated lymphoid tissue (MALT) lymphoma. Clin J Gastroenterol. 2013 Apr;6(2):150-5. doi: 10.1007/s12328-013-0362-5. Epub 2013 Feb 7. PMID: 26181453.

281: Hsu CT, Chang HR, Lee JK, Weng JH, Kao PF. FDG PET/CT repeatedly demonstrated hepatic cyst infection in a patient with autosomal dominant polycystic kidney disease. Clin Nucl Med. 2013 Apr;38(4):e188-90. doi: 10.1097/RLU.0b013e318266d056. Erratum in: Clin Nucl Med. 2013 Jul;38(7):589. PMID: 23429392.

282: Brackers de Hugo L, Ffrench M, Broussolle C, Sève P. Granulomatous lesions in bone marrow: clinicopathologic findings and significance in a study of 48 cases. Eur J Intern Med. 2013 Jul;24(5):468-73. doi: 10.1016/j.ejim.2012.11.008. Epub 2013 Feb 13. PMID: 23414770.

283: Vatakis DN, Bristol GC, Kim SG, Levin B, Liu W, Radu CG, Kitchen SG, Zack JA. Using the BLT humanized mouse as a stem cell based gene therapy tumor model. J Vis Exp. 2012 Dec 18;(70):e4181. doi: 10.3791/4181. PMID: 23271478; PMCID: PMC3576416.

284: Chopra A. ¹¹¹In-Labeled Ac-TZ14011 peptide (a chemokine receptor 4 antagonist) conjugated to CyAL5.5 (a fluorescent dye) through a multifunctional single-attachment point (MSAP) reagent. 2012 Nov 29 [updated 2012 Dec 27]. In: Molecular Imaging and Contrast Agent Database (MICAD) [Internet]. Bethesda (MD): National Center for Biotechnology Information (US); 2004–2013. PMID: 23285497.

285: Banzo J, Ubieto MA, Gil D, Prats E, Razola P, Tardín L, Andrés A, Rambalde EF, Ayala SM, Cáncer L, Velilla J. Diagnóstico mediante 18F-FDG PET-TAC de infección quística hepática en paciente con enfermedad poliquística renal autosómica dominante y fiebre de origen desconocido [18F-FDG PET/CT diagnosis of liver cyst infection in a patient with autosomal dominant polycystic kidney disease and fever of unknown origin]. Rev Esp Med Nucl Imagen Mol. 2013 May-Jun;32(3):187-9. Spanish. doi: 10.1016/j.remn.2012.09.003. Epub 2012 Nov 13. PMID: 23153986.

286: Jouret F, Lhommel R, Devuyst O, Annet L, Pirson Y, Hassoun Z, Kanaan N. Diagnosis of cyst infection in patients with autosomal dominant polycystic kidney disease: attributes and limitations of the current modalities. Nephrol Dial Transplant. 2012 Oct;27(10):3746-51. doi: 10.1093/ndt/gfs352. PMID: 23114901.

287: Bannas P, Habermann CR, Yamamura J, Bley TA. Severe haemorrhage after liver biopsy of malignant B-cell lymphoma mimicking hepatic infection. Rofo. 2013 Feb;185(2):164-6. doi: 10.1055/s-0032-1325443. Epub 2012 Oct 29. PMID: 23108895.

288: Vávra P, Třeška V, Ostruszka P, Sutnar A, Fichtl J, Hozáková L, Mitták M, Horáček J. Chirurgické řešení komplikované jaterní echinokokózy u dvou bulharských občanů na dvou pracovištích v České republice [Surgical treatment of complicated hepatic echinococcosis in two Bulgarian citizens at two surgical departments in the Czech Republic]. Rozhl Chir. 2012 Jul;91(7):381-7. Czech. PMID: 23078257.

289: Mourin G, Badia A, Cazes A, Planquette B. An unusual cause of pulmonary artery pseudoaneurysm: acrylate embolism. Interact Cardiovasc Thorac Surg. 2012 Dec;15(6):1082-4. doi: 10.1093/icvts/ivs314. Epub 2012 Sep 18. PMID: 22990635; PMCID: PMC3501291.

290: Valero V 3rd, Cosgrove D, Herman JM, Pawlik TM. Management of perihilar cholangiocarcinoma in the era of multimodal therapy. Expert Rev Gastroenterol Hepatol. 2012 Aug;6(4):481-95. doi: 10.1586/egh.12.20. PMID: 22928900; PMCID: PMC3538366.

291: Kumar V, Boddeti DK. (68)Ga-radiopharmaceuticals for PET imaging of infection and inflammation. Recent Results Cancer Res. 2013;194:189-219. doi:

10.1007/978-3-642-27994-2_11. PMID: 22918761.

292: Liu SR, Xiao YY, Le Pivert PJ, Wu B, Zhang X, Ma XY, Ren C. CT-guided percutaneous chemoablation using an ethanol-ethiodol-doxorubicin emulsion for the treatment of metastatic lymph node carcinoma: a comparative study. Technol Cancer Res Treat. 2013 Apr;12(2):165-72. doi: 10.7785/tcrt.2012.500254. Epub 2012 Aug 10. Erratum in: Technol Cancer Res Treat. 2013 Oct;12(5):483. Liu, S-H [corrected to Liu, S-R]. PMID: 22905808.

293: Jung CY, Hikima J, Ohtani M, Jang HB, del Castillo CS, Nho SW, Cha IS, Park SB, Aoki T, Jung TS. Recombinant interferon-γ activates immune responses against Edwardsiella tarda infection in the olive flounder, Paralichthys olivaceus. Fish Shellfish Immunol. 2012 Aug;33(2):197-203. doi: 10.1016/j.fsi.2012.04.015. Epub 2012 May 16. PMID: 22609730.

294: Mori E, Akai Y, Matsumoto T, Kawaratani H, Horii M, Iwano M, Uemura S, Nonomura A, Fukui H, Saito Y. Hepatic cyst infection in a healthy older male. BMJ Case Rep. 2012 Mar 20;2012:bcr0420114136. doi: 10.1136/bcr.04.2011.4136. PMID: 22605693; PMCID: PMC3316800.

295: Vase MØ, Hellberg YK, Larsen CS, Petersen E, Schaumburg H, Bendix K, Ravel C, Bastien P, Christensen M, d'Amore F. Development of splenic marginal zone lymphoma in a HIV-negative patient with visceral leishmaniasis. Acta Haematol. 2012;128(1):20-2. doi: 10.1159/000337341. Epub 2012 May 8. PMID: 22572474.

296: Chopra A. <i>N</i>'-Fluorescein-<i>N</i>''-[4-<i>O</i>-(β-d-glucopyranuronic acid)-3-difluoromethylphenyl]-<i>S</i>-methylthiourea (FITC-TrapG) and <i>N</i>'-(p-aminophenyl)thioether of IR-820-<i>N</i>''-[4-<i>O</i>-(β-d-glucopyranuronic acid)-3-difluoromethylphenyl]-<i>S</i>-methylthiourea (NIR-TrapG). 2012 Apr 10 [updated 2012 May 10]. In: Molecular Imaging and Contrast Agent Database (MICAD) [Internet]. Bethesda (MD): National Center for Biotechnology Information (US); 2004–2013. PMID: 22593947.

297: Chopra A. Methyl ester of 1-<i>O</i>-(4-(2-[¹⁸F]fluoroethyl-carbamoyloxymethyl)-2-nitrophenyl)-<i>O</i>-β-d-glucopyronuronate. 2012 Apr 4 [updated 2012 Apr 27]. In: Molecular Imaging and Contrast Agent Database (MICAD) [Internet]. Bethesda (MD): National Center for Biotechnology Information (US); 2004–2013. PMID: 22553889.

298: Chopra A. 1-<i>O</i>-(4-(2-[¹⁸F]Fluoroethyl-carbamoyloxymethyl)-2-nitrophenyl)-<i>O</i>-β-d-glucopyronuronate. 2012 Mar 28 [updated 2012 Apr 27]. In: Molecular Imaging and Contrast Agent Database (MICAD) [Internet]. Bethesda (MD): National Center for Biotechnology Information (US); 2004–2013. PMID: 22553888.

299: Valavoor SH, Ashraf Z, Narwal R, Ratnam S. Conservative management of post-transplant central nervous system lymphoma. Int Urol Nephrol. 2013 Aug;45(4):1219-22. doi: 10.1007/s11255-012-0146-8. Epub 2012 Apr 3. PMID: 22476860.

300: Decaens T, Luciani A, Itti E, Hulin A, Roudot-Thoraval F, Laurent A, Zafrani ES, Mallat A, Duvoux C. Phase II study of sirolimus in treatment-naive patients with advanced hepatocellular carcinoma. Dig Liver Dis. 2012 Jul;44(7):610-6. doi: 10.1016/j.dld.2012.02.005. Epub 2012 Mar 27. PMID: 22459565.

301: Yang JI, Kang JM, Byun HJ, Chung GE, Yim JY, Park MJ, Lee JH, Yoon JH, Lee HS. Metastatic hepatocellular carcinoma presenting as facial nerve palsy and facial pain. Korean J Hepatol. 2011 Dec;17(4):319-22. doi: 10.3350/kjhep.2011.17.4.319. PMID: 22310797; PMCID: PMC3304660.

302: Sainaresh V, Jain Sh, Patel H, Shah P, Vanikar A, Trivedi H. Post transplant urinary tract infection in Autosomal dominant polycystic kidney disease a perpetual diagnostic dilema - 18-fluorodeoxyglucose - Positron emission computerized tomography - A valuable tool. Indian J Nucl Med. 2011 Apr;26(2):109-11. doi: 10.4103/0972-3919.90266. PMID: 22174521; PMCID: PMC3237213.

303: Cheng G, Torigian DA, Alavi A. FDG PET/CT and MRI findings in a patient with focal xanthogranulomatous pyelonephritis mimicking cystic renal malignancy. Clin Nephrol. 2011 Dec;76(6):484-6. doi: 10.5414/cn106762. PMID: 22105452.

304: Miyagi S, Sekiguchi S, Kawagishi N, Akamatsu Y, Satoh K, Takeda I, Fukushima D, Kobayashi Y, Tokodai K, Fujimori K, Satomi S. Rituximab therapy and reduction of immunosuppression to rescue graft function after renal posttransplantation lymphoproliferative disorder found by macrohematuria in a pancreas and kidney transplant recipient: a case report. Transplant Proc. 2011 Nov;43(9):3299-301. doi: 10.1016/j.transproceed.2011.10.002. PMID: 22099782.

305: Agrawal K, Bhattacharya A, Singh SK, Manohar K, Kashyap R, Mittal BR. Polycystic kidney disease: renal cyst infection detected on F-18 FDG PET/CT. Clin Nucl Med. 2011 Dec;36(12):1122-3. doi: 10.1097/RLU.0b013e31821a2bcb. PMID: 22064087.

306: Piccoli GB, Arena V, Consiglio V, Deagostini MC, Pelosi E, Douroukas A, Penna D, Cortese G. Positron emission tomography in the diagnostic pathway for intracystic infection in adpkd and "cystic" kidneys. a case series. BMC Nephrol. 2011 Sep 29;12:48. doi: 10.1186/1471-2369-12-48. PMID: 21957932; PMCID: PMC3197475.

307: Nooka A, Shenoy PJ, Sinha R, Lonial S, Flowers CR. Hepatitis C reactivation in patients who have diffuse large B-cell lymphoma treated with rituximab: a case report and review of literature. Clin Lymphoma Myeloma Leuk. 2011 Oct;11(5):379-84. doi: 10.1016/j.clml.2011.04.005. Epub 2011 May 12. PMID: 21729690.

308: Jouret F, Lhommel R, Beguin C, Devuyst O, Pirson Y, Hassoun Z, Kanaan N. Positron-emission computed tomography in cyst infection diagnosis in patients with autosomal dominant polycystic kidney disease. Clin J Am Soc Nephrol. 2011 Jul;6(7):1644-50. doi: 10.2215/CJN.06900810. Epub 2011 Jun 23. PMID: 21700816.

309: Raina S, Honer M, Krämer SD, Liu Y, Wang X, Segerer S, Wüthrich RP, Serra AL. Anti-VEGF antibody treatment accelerates polycystic kidney disease. Am J Physiol Renal Physiol. 2011 Oct;301(4):F773-83. doi: 10.1152/ajprenal.00058.2011. Epub 2011 Jun 15. PMID: 21677148.

310: Chopra A. ⁶⁴Cu-Labeled bis-1,4,7,10-tetraazacyclododecane-<i>N</i>,<i>N</i>',<i>N</i>,<i>N</i>'-tetraacetic acid conjugated hypericin. 2011 May 3 [updated 2011 May 26]. In: Molecular Imaging and Contrast Agent Database (MICAD) [Internet]. Bethesda (MD): National Center for Biotechnology Information (US); 2004–2013. PMID: 21656982.

311: Berent-Spillson A, Love T, Pop-Busui R, Sowers M, Persad CC, Pennington KP, Eyvazaddeh AD, Padmanabhan V, Zubieta JK, Smith YR. Insulin resistance influences central opioid activity in polycystic ovary syndrome. Fertil Steril. 2011 Jun 30;95(8):2494-8. doi: 10.1016/j.fertnstert.2011.03.031. Epub 2011 Apr 12. PMID: 21486668; PMCID: PMC3124614.

312: Tatli S, Gerbaudo VH, Feeley CM, Shyn PB, Tuncali K, Silverman SG. PET/CT-guided percutaneous biopsy of abdominal masses: initial experience. J Vasc Interv Radiol. 2011 Apr;22(4):507-14. doi: 10.1016/j.jvir.2010.12.035. Epub 2011 Mar 2. PMID: 21367619.

313: Singh S, Bundrick JB. An uncommon infection in an uncommon host. Scand J Infect Dis. 2011 Aug;43(8):661-3. doi: 10.3109/00365548.2011.552073. Epub 2011 Jan 25. PMID: 21265585.

314: Kaneko K, Nishie A, Arima F, Yoshida T, Ono K, Omagari J, Honda H. A case of diffuse-type primary hepatic lymphoma mimicking diffuse hepatocellular carcinoma. Ann Nucl Med. 2011 May;25(4):303-7. doi: 10.1007/s12149-010-0460-0. Epub 2011 Jan 14. PMID: 21234726.

315: Wimsatt J, Withrow SJ, Danner D, Powers B, Hagler T, Pritzker KP. Multicystic bone disease (Gorham-Stout syndrome) in a spider monkey (Ateles

geoffroyi). J Med Primatol. 2011 Apr;40(2):61-70. doi: 10.1111/j.1600-0684.2010.00457.x. Epub 2010 Dec 15. PMID: 21158869.

316: Piccoli GB, Arena V, Consiglio V, Depascale A, Deagostini MC. Positron emission tomography: a precious tool in the challenge of the infected cysts in ADPKD. NDT Plus. 2010 Oct;3(5):492-3. doi: 10.1093/ndtplus/sfq104. Epub 2010 Jun 22. PMID: 25984064; PMCID: PMC4421707.

317: Banzo J, Ubieto MA, Prats E, Razola P, Tardin L, Andrés A, Santapau A, Parra A. ^{18}F-FDG PET-TAC en mononucleosis por citomegalovirus [^{18}F-FDG PET-CT in cytomegalovirus-induced mononucleosis]. Rev Esp Med Nucl. 2010 Nov-Dec;29(6):304-7. Spanish. doi: 10.1016/j.remn.2010.03.008. Epub 2010 Sep 24. PMID: 20869792.

318: Makis W, Stern J. Hepatitis C-related primary effusion lymphoma of the pleura and peritoneum, imaged with F-18 FDG PET/CT. Clin Nucl Med. 2010 Oct;35(10):797-9. doi: 10.1097/RLU.0b013e3181ef09b1. PMID: 20838290.

319: Cheng W, Li F, Zhuang H, Zhong D, Wu C, Zhu Z. Hepatic paragonimiasis revealed by FDG PET/CT. Clin Nucl Med. 2010 Sep;35(9):726-8. doi: 10.1097/RLU.0b013e3181ea33e5. PMID: 20706055.

320: Haist V, Hirschfeld SG, Mallig C, Fehr M, Baumgärtner W. Pathologic fracture of the femur due to endometrial adenocarcinoma metastasis in a female pet rabbit (Oryctolagus cuniculi). Berl Munch Tierarztl Wochenschr. 2010 Jul-Aug;123(7-8):346-51. PMID: 20690547.

321: Nourbakhsh E, Goodman S, Nash S, Nugent K. Generalized lymphadenopathy secondary to an invasive fungal infection in an apparently healthy patient. Am J Med Sci. 2010 Jul;340(1):84-8. doi: 10.1097/MAJ.0b013e3181df7ff8. PMID: 20610974.

322: Concejero AM, Yong CC, Chen CL, Lu HI, Wang CC, Wang SH, Liu YW, Yang CH, Cheng YF, Jawan B. Solitary pulmonary nodule in the liver transplant candidate: importance of diagnosis and treatment. Liver Transpl. 2010 Jun;16(6):760-6. doi: 10.1002/lt.22066. PMID: 20517910.

323: Kumar R, Shandal V, Shamim SA, Halanaik D, Malhotra A. Clinical applications of PET and PET/CT in pediatric malignancies. Expert Rev Anticancer Ther. 2010 May;10(5):755-68. doi: 10.1586/era.10.12. PMID: 20470007.

324: Vaglio A, Manenti L, Mancini C, Chierici E, Cobelli R, Bacci F, Palmisano A, Buzio C, Bignardi L, Maggiore U. EBV-associated leukoencephalopathy with late onset of central nervous system lymphoma in a kidney transplant recipient. Am J Transplant. 2010 Apr;10(4):947-951. doi: 10.1111/j.1600-6143.2009.02999.x. PMID:

20420644.

325: Kawaguchi M, Asada Y, Terada T, Takehara A, Munemoto Y, Fujisawa K, Mitsui T, Iida Y, Miura S, Sudo Y. Aggressive recurrence of gastric cancer as a granulocyte-colony-stimulating factor-producing tumor. Int J Clin Oncol. 2010 Apr;15(2):191-5. doi: 10.1007/s10147-010-0023-3. Epub 2010 Feb 24. PMID: 20179985.

326: Welling MM, Alberto R. Performance of a 99mTc-labelled 1-thio-beta-D-glucose 2,3,4,6-tetra-acetate analogue in the detection of infections and tumours in mice: a comparison with [18F]FDG. Nucl Med Commun. 2010 Mar;31(3):239-48. doi: 10.1097/MNM.0b013e32833501e4. PMID: 20032803.

327: Inui K, Takahashi Y, Komeichi H, Katsuta Y, Shimizu S, Mizuno K. Successful transcatheter chemoembolization for acute jaundice in a patient with advanced hepatocellular carcinoma and portal vein tumor thrombosis: a case report. J Nippon Med Sch. 2009 Aug;76(4):217-20. doi: 10.1272/jnms.76.217. PMID: 19755798.

328: Tan IB, Padhy AK, Thng CH, Osmany S, Magsombol B, Ho YH, Tham CK, Quek R, Tao M, Lim ST. Intensely hypermetabolic extra-axial brainstem tumor in Erdheim-Chester disease. Clin Nucl Med. 2009 Sep;34(9):604-7. doi: 10.1097/RLU.0b013e3181b06a7e. PMID: 19692824.

329: Hard GC, Flake GP, Sills RC. Re-evaluation of kidney histopathology from 13-week toxicity and two-year carcinogenicity studies of melamine in the F344 rat: morphologic evidence of retrograde nephropathy. Vet Pathol. 2009 Nov;46(6):1248-57. doi: 10.1354/vp.08-VP-0317-F-FL. Epub 2009 Jul 15. PMID: 19605901.

330: Chopra A. ⁶⁴Cu-Labeled 1,1′-{1,4-phenylenebis(methylene)}-bis{1,4,8,11-tetraaza-cyclotetradecane}. 2009 Jun 8 [updated 2009 Jul 7]. In: Molecular Imaging and Contrast Agent Database (MICAD) [Internet]. Bethesda (MD): National Center for Biotechnology Information (US); 2004–2013. PMID: 20641708.

331: Chopra A. ¹¹¹In-Labeled diethylenetriamine-pentaacetic acid-Ac-TZ14011. 2009 Jun 10 [updated 2009 Jul 7]. In: Molecular Imaging and Contrast Agent Database (MICAD) [Internet]. Bethesda (MD): National Center for Biotechnology Information (US); 2004–2013. PMID: 20641260.

332: Desouza RM, Prachalias A, Srinivasan P, O'Doherty M, Olsburgh J. Differentiation between infection in kidney and liver cysts in autosomal dominant polycystic kidney disease: use of PET-CT in diagnosis and to guide management. Transplant Proc. 2009 Jun;41(5):1942-5. doi: 10.1016/j.transproceed.2008.10.102. PMID: 19545761.

333: Jiménez-Bonilla JF, Quirce R, Calabia ER, Banzo I, Martínez-Rodríguez I, Carril JM. Hepatorenal polycystic disease and fever: diagnostic contribution of gallium citrate Ga 67 scan and fluorine F 18 FDG-PET/CT. Eur Urol. 2011 Feb;59(2):297-9. doi: 10.1016/j.eururo.2009.05.049. Epub 2009 Jun 6. PMID: 19524352.

334: Sallée M, Rafat C, Zahar JR, Paulmier B, Grünfeld JP, Knebelmann B, Fakhouri F. Cyst infections in patients with autosomal dominant polycystic kidney disease. Clin J Am Soc Nephrol. 2009 Jul;4(7):1183-9. doi: 10.2215/CJN.01870309. Epub 2009 May 21. PMID: 19470662; PMCID: PMC2709515.

335: Rossleigh MA. Scintigraphic imaging in renal infections. Q J Nucl Med Mol Imaging. 2009 Feb;53(1):72-7. PMID: 19182730.

336: Legler M, Kothe R, Rautenschlein S, Kummerfeld N. Detektion des Psittacid Herpesvirus 1 bei Amazonen mit papillomatösen Kloakenveränderungen (Internal papillomatosis of parrots, IPP) aus einer Gemeinschaftshaltung verschiedener Papageienarten [Detection of psittacid herpesvirus 1 in Amazon parrots with cloacal papilloma (internal papillomatosis of parrots, IPP) in an aviary of different psittacine species]. Dtsch Tierarztl Wochenschr. 2008 Dec;115(12):461-70. German. PMID: 19113029.

337: Gu YK, Wu PH, Fan WJ, Huang JH, Zhang L, Gao F. [Clinical value of CT guided radiofrequency ablation in treatment of post-operational resectable liver metastatic tumor from colorectal carcinoma]. Zhonghua Yi Xue Za Zhi. 2008 Oct 28;88(39):2748-50. Chinese. PMID: 19080447.

338: Salem N, Kuang Y, Wang F, Maclennan GT, Lee Z. PET imaging of hepatocellular carcinoma with 2-deoxy-2[18F]fluoro-D-glucose, 6-deoxy-6[18F] fluoro-D-glucose, [1-11C]-acetate and [N-methyl-11C]-choline. Q J Nucl Med Mol Imaging. 2009 Apr;53(2):144-56. Epub 2008 Nov 28. PMID: 19039303.

339: Nau KC, Lewis WD. Multiple myeloma: diagnosis and treatment. Am Fam Physician. 2008 Oct 1;78(7):853-9. PMID: 18841734.

340: Doi H, Horiike N, Hiraoka A, Koizumi Y, Yamamoto Y, Hasebe A, Ichikawa S, Yano M, Miyamoto Y, Ninomiya T, Ishimaru Y, Miyagawa M, Takamura K, Kawasaki H, Kozuka T, Maeda T, Yoshino T. Primary hepatic marginal zone B cell lymphoma of mucosa-associated lymphoid tissue type: case report and review of the literature. Int J Hematol. 2008 Nov;88(4):418-423. doi: 10.1007/s12185-008-0153-9. Epub 2008 Sep 23. PMID: 18807227.

341: Kaira K, Ishizuka T, Tanaka H, Tanaka Y, Yanagitani N, Sunaga N, Hisada T, Ishizuka T, Mori M. Lung cancer producing granulocyte colony-stimulating factor

and rapid spreading to peritoneal cavity. J Thorac Oncol. 2008 Sep;3(9):1054-5. doi: 10.1097/JTO.0b013e3181834f7b. PMID: 18758311.

342: Soussan M, Sberro R, Wartski M, Fakhouri F, Pecking AP, Alberini JL. Diagnosis and localization of renal cyst infection by 18F-fluorodeoxyglucose PET/CT in polycystic kidney disease. Ann Nucl Med. 2008 Jul;22(6):529-31. doi: 10.1007/s12149-008-0150-3. Epub 2008 Aug 1. PMID: 18670861.

343: Bonatti H, Huguet K, McLaughlin S, Stockland A, Aranda-Michel J, Al Haddad M, Dougherty M, Fitzpatrick P, Kim G, Martin K, Hinder R, Nguyen JH. Whipple's procedure in a renal transplant recipient with polycystic liver disease. JOP. 2008 Jul 10;9(4):515-9. PMID: 18648145.

344: Lee SY, Kim HJ, Park SY, Park YH, Chung SK. Myxoid liposarcoma involving the liver, subcutaneous tissue, and epidural space in a polycystic disease patient. Clin Nucl Med. 2008 Jul;33(7):507-9. doi: 10.1097/RLU.0b013e3181779240. PMID: 18580245.

345: Chundru S, Wong CY, Wu D, Balon H, Palka J, Chang CY, Gaskill M, Cheng CY, Huang WS, Fink-Bennett D. Granulomatous disease: is it a nuisance or an asset during PET/computed tomography evaluation of lung cancers? Nucl Med Commun. 2008 Jul;29(7):623-7. doi: 10.1097/MNM.0b013e3282fdc979. PMID: 18528184.

346: Park BH, Hwang T, Liu TC, Sze DY, Kim JS, Kwon HC, Oh SY, Han SY, Yoon JH, Hong SH, Moon A, Speth K, Park C, Ahn YJ, Daneshmand M, Rhee BG, Pinedo HM, Bell JC, Kirn DH. Use of a targeted oncolytic poxvirus, JX-594, in patients with refractory primary or metastatic liver cancer: a phase I trial. Lancet Oncol. 2008 Jun;9(6):533-42. doi: 10.1016/S1470-2045(08)70107-4. Epub 2008 May 19. Erratum in: Lancet Oncol. 2008 Jul;9(7):613. PMID: 18495536.

347: Gaujoux S, Terris B, Bertherat J, Vilgrain V, Ruszniewski P, Dousset B. Massive postoperative ascites following pancreatic cysts fenestration in a patient with von Hippel-Lindau disease. Gastroenterol Clin Biol. 2008 Nov;32(11):910-3. doi: 10.1016/j.gcb.2008.01.041. Epub 2008 May 7. PMID: 18467057.

348: Lustberg MB, Aras O, Meisenberg BR. FDG PET/CT findings in acute adult mononucleosis mimicking malignant lymphoma. Eur J Haematol. 2008 Aug;81(2):154-6. doi: 10.1111/j.1600-0609.2008.01088.x. Epub 2008 May 6. PMID: 18462255.

349: Chopra A. 1-(2′-Deoxy-2′-[¹⁸F]-fluoro-β-D-arabinofuranosyl)thymine. 2008 Jan 9 [updated 2008 Feb 14]. In: Molecular Imaging and Contrast Agent Database (MICAD) [Internet]. Bethesda (MD): National Center for Biotechnology Information (US); 2004–2013. PMID: 20641443.

350: Sedlacek M, Cotter JG, Suriawinata AA, Kaneko TM, Zuckerman RA, Parsonnet J, Block CA. Mucormycosis peritonitis: more than 2 years of disease-free follow-up after posaconazole salvage therapy after failure of liposomal amphotericin B. Am J Kidney Dis. 2008 Feb;51(2):302-6. doi: 10.1053/j.ajkd.2007.09.026. PMID: 18215708.

351: Chopra A. 2′-Fluoro-5-([¹¹C]-methyl)-1-β-D-arabinofuranosyluracil. 2008 Jan 2 [updated 2008 Jan 24]. In: Molecular Imaging and Contrast Agent Database (MICAD) [Internet]. Bethesda (MD): National Center for Biotechnology Information (US); 2004–2013. PMID: 20641855.

352: Moulin-Romsee G, Mortelmans L. PET versus PET-CT in patient with suspicion of non-Hodgkin lymphoma recurrence. Clin Nucl Med. 2007 Dec;32(12):954-5. doi: 10.1097/RLU.0b013e3181597680. PMID: 18030052.

353: Mocherla B, Kim J, Roayaie S, Kim S, Machac J, Kostakoglu L. FDG PET/CT imaging to rule out extrahepatic metastases before liver transplantation. Clin Nucl Med. 2007 Dec;32(12):947-8. doi: 10.1097/RLU.0b013e3181598cef. PMID: 18030049.

354: Salskov A, Tammisetti VS, Grierson J, Vesselle H. FLT: measuring tumor cell proliferation in vivo with positron emission tomography and 3'-deoxy-3'-[18F]fluorothymidine. Semin Nucl Med. 2007 Nov;37(6):429-39. doi: 10.1053/j.semnuclmed.2007.08.001. Erratum in: Semin Nucl Med. 2008 Mar;38(2):148. PMID: 17920350.

355: Staebler S, Steinmetz H, Keller S, Deplazes P. First description of natural Echinococcus multilocularis infections in chinchilla (Chinchilla laniger) and Prevost's squirrel (Callosciurus prevostii borneoensis). Parasitol Res. 2007 Nov;101(6):1725-7. doi: 10.1007/s00436-007-0717-2. Epub 2007 Sep 1. PMID: 17768640.

356: van de Glind G, de Graaf S, Klein C, Cornelissen M, Maecker B, Loeffen J. Intrathecal rituximab treatment for pediatric post-transplant lymphoproliferative disorder of the central nervous system. Pediatr Blood Cancer. 2008 Apr;50(4):886-8. doi: 10.1002/pbc.21297. PMID: 17668865.

357: Guillot J, Bouree P. Les helminthes transmissibles des carnivores domestiques à l'homme : évaluation des risques et stratégies de prévention [Zoonotic worms from carnivorous pets: risk assessment and prevention]. Bull Acad Natl Med. 2007 Jan;191(1):67-78; discussion 79-81. French. PMID: 17645108.

358: Hayashi Y, Ishii Y, Arai R, Obara K, Kamada A, Takizawa H, Hase I, Mashio K, Yamada I, Takemasa A, Sugiyama K, Fukushima Y, Fukuda T. [A case of group G

Streptococcus sepsis, chest wall abscess, and vertebral osteomyelitis mimicking a primary lung cancer with bone metastasis]. Nihon Kokyuki Gakkai Zasshi. 2007 Jan;45(1):76-80. Japanese. PMID: 17313032.

359: Roarke MC, Collins JM, Nguyen BD. Indolent enterococcal abscess mimicking recurrent renal cell carcinoma on MR imaging and PET/CT after radiofrequency ablation. J Vasc Interv Radiol. 2006 Nov;17(11 Pt 1):1851-4. doi: 10.1097/01.RVI.0000242169.99178.CB. PMID: 17142718.

360: Nasr Ben Ammar C, Chaari N, Kochbati L, Besbes M, Maalej M. Lymphome non hodgkinien primitif du foie: à propos d'un cas et revue de la littérature [Primary non-Hodgkin lymphoma of the liver: case report and review of the literature]. Cancer Radiother. 2006 Dec;10(8):595-601. French. doi: 10.1016/j.canrad.2006.09.117. Epub 2006 Nov 29. PMID: 17137818.

361: Teshigawara K, Kakizaki S, Sohara N, Hashida T, Tomizawa Y, Sato K, Takagi H, Mori M, Hoshino K, Mogi K. Solitary mandibular metastasis as an initial manifestation of hepatocellular carcinoma. Acta Med Okayama. 2006 Aug;60(4):243-7. doi: 10.18926/AMO/30713. PMID: 16943863.

362: Bryant AS, Cerfolio RJ. The maximum standardized uptake values on integrated FDG-PET/CT is useful in differentiating benign from malignant pulmonary nodules. Ann Thorac Surg. 2006 Sep;82(3):1016-20. doi: 10.1016/j.athoracsur.2006.03.095. PMID: 16928527.

363: Rehbinder B, Wullstein Ch, Bechstein WO, Probst M, Engels K, Kriener S, Döbert N, Schwarz W, Brixner V, Steffan D, Gauer S, Geiger H, Hauser IA. Epstein-barr virus-associated posttransplant lymphoproliferative disorder of donor origin after simultaneous pancreas-kidney transplantation limited to pancreas allograft: A case report. Am J Transplant. 2006 Oct;6(10):2506-11. doi: 10.1111/j.1600-6143.2006.01464.x. Epub 2006 Jul 25. PMID: 16869797.

364: Jacene HA, Stearns V, Wahl RL. Lymphadenopathy resulting from acute hepatitis C infection mimicking metastatic breast carcinoma on FDG PET/CT. Clin Nucl Med. 2006 Jul;31(7):379-81. doi: 10.1097/01.rlu.0000222675.10765.14. PMID: 16785802.

365: Homburg R. Pregnancy complications in PCOS. Best Pract Res Clin Endocrinol Metab. 2006 Jun;20(2):281-92. doi: 10.1016/j.beem.2006.03.009. PMID: 16772158.

366: Chang JM, Lee HJ, Goo JM, Lee HY, Lee JJ, Chung JK, Im JG. False positive and false negative FDG-PET scans in various thoracic diseases. Korean J Radiol. 2006 Jan-Mar;7(1):57-69. doi: 10.3348/kjr.2006.7.1.57. PMID: 16549957; PMCID: PMC2667579.

367: Noguera Aguilar JF, Vicens Arbona JC, Morales Soriano R, Ibarra de la Rosa J, Arrivi García-Ramos A, Cuadra Coll M, Dolz Abadía C, Pujol Tugores JJ. Liver resection in metastatic colorectal cancer: a multidisciplinary approach. Rev Esp Enferm Dig. 2005 Nov;97(11):786-93. English, Spanish. doi: 10.4321/s1130-01082005001100003. PMID: 16438622.

368: Carrasco L, Raya AI, Núñez A, Gómez-Laguna J, Hernández S, Dubey JP. Fatal toxoplasmosis and concurrent Calodium hepaticum infection in Korean squirrels (Tanias sibericus). Vet Parasitol. 2006 Apr 15;137(1-2):180-3. doi: 10.1016/j.vetpar.2005.11.026. Epub 2006 Jan 6. PMID: 16406358.

369: Patel T. Cholangiocarcinoma. Nat Clin Pract Gastroenterol Hepatol. 2006 Jan;3(1):33-42. doi: 10.1038/ncpgasthep0389. PMID: 16397610.

370: Medical Advisory Secretariat. Extracorporeal photophoresis: an evidence-based analysis. Ont Health Technol Assess Ser. 2006;6(6):1-82. Epub 2006 Mar 1. PMID: 23074497; PMCID: PMC3379535.

371: Goodfellow M, Shaw S, Morgan E. Imported disease of dogs and cats exotic to Ireland: Echinococcus multilocularis. Ir Vet J. 2006 Apr 1;59(4):214-6. doi: 10.1186/2046-0481-59-4-214. PMID: 21851681; PMCID: PMC3113889.

372: Rogers BE, Parry JJ, Andrews R, Cordopatis P, Nock BA, Maina T. MicroPET imaging of gene transfer with a somatostatin receptor-based reporter gene and (94m)Tc-Demotate 1. J Nucl Med. 2005 Nov;46(11):1889-97. PMID: 16269604.

373: Sezer S, Tutal E, Arat Z, Akçay A, Celik H, Ozdemir FN, Haberal M. Peritoneal transport status influence on atherosclerosis/inflammation in CAPD patients. J Ren Nutr. 2005 Oct;15(4):427-34. doi: 10.1053/j.jrn.2005.07.007. PMID: 16198934.

374: Peñuelas I, Mazzolini G, Boán JF, Sangro B, Martí-Climent J, Ruiz M, Ruiz J, Satyamurthy N, Qian C, Barrio JR, Phelps ME, Richter JA, Gambhir SS, Prieto J. Positron emission tomography imaging of adenoviral-mediated transgene expression in liver cancer patients. Gastroenterology. 2005 Jun;128(7):1787-95. doi: 10.1053/j.gastro.2005.03.024. PMID: 15940613.

375: Teruya-Feldstein J, Chiao E, Filippa DA, Lin O, Comenzo R, Coleman M, Portlock C, Noy A. CD20-negative large-cell lymphoma with plasmablastic features: a clinically heterogenous spectrum in both HIV-positive and -negative patients. Ann Oncol. 2004 Nov;15(11):1673-9. doi: 10.1093/annonc/mdh399. PMID: 15520070.

376: Chen X, Hou Y, Tohme M, Park R, Khankaldyyan V, Gonzales-Gomez I, Bading JR, Laug WE, Conti PS. Pegylated Arg-Gly-Asp peptide: 64Cu labeling and PET

imaging of brain tumor alphavbeta3-integrin expression. J Nucl Med. 2004 Oct;45(10):1776-83. PMID: 15471848.

377: Aprile C. Radiolabelled peptides and low molecular weight proteins in metabolic diseases. Q J Nucl Med. 2003 Dec;47(4):321-36. PMID: 14973422.

378: Lara PN Jr, Quinn DI, Margolin K, Meyers FJ, Longmate J, Frankel P, Mack PC, Turrell C, Valk P, Rao J, Buckley P, Wun T, Gosselin R, Galvin I, Gumerlock PH, Lenz HJ, Doroshow JH, Gandara DR; California Cancer Consortium. SU5416 plus interferon alpha in advanced renal cell carcinoma: a phase II California Cancer Consortium Study with biological and imaging correlates of angiogenesis inhibition. Clin Cancer Res. 2003 Oct 15;9(13):4772-81. PMID: 14581348.

379: Bleeker-Rovers CP, de Sévaux RG, van Hamersvelt HW, Corstens FH, Oyen WJ. Diagnosis of renal and hepatic cyst infections by 18-F-fluorodeoxyglucose positron emission tomography in autosomal dominant polycystic kidney disease. Am J Kidney Dis. 2003 Jun;41(6):E18-21. doi: 10.1016/s0272-6386(03)00368-8. PMID: 12776306.

380: Silvestri GA, Tanoue LT, Margolis ML, Barker J, Detterbeck F; American College of Chest Physicians. The noninvasive staging of non-small cell lung cancer: the guidelines. Chest. 2003 Jan;123(1 Suppl):147S-156S. doi: 10.1378/chest.123.1_suppl.147s. PMID: 12527574.

381: Jafari M, Forsberg J, Gilcher RO, Smith JW, Crutcher JM, McDermott M, Brown BR, George JN. Salmonella sepsis caused by a platelet transfusion from a donor with a pet snake. N Engl J Med. 2002 Oct 3;347(14):1075-8. doi: 10.1056/NEJMoa021050. PMID: 12362008.

382: Sugimura K, Tanaka T, Tanaka Y, Takano H, Kanagawa K, Sakamoto N, Ikemoto S, Kawashima H, Nakatani T. Decreased sulfotransferase SULT1C2 gene expression in DPT-induced polycystic kidney. Kidney Int. 2002 Sep;62(3):757-62. doi: 10.1046/j.1523-1755.2002.00512.x. PMID: 12164856.

383: Zhao S, Kuge Y, Tsukamoto E, Mochizuki T, Kato T, Hikosaka K, Nakada K, Hosokawa M, Kohanawa M, Tamaki N. Fluorodeoxyglucose uptake and glucose transporter expression in experimental inflammatory lesions and malignant tumours: effects of insulin and glucose loading. Nucl Med Commun. 2002 Jun;23(6):545-50. doi: 10.1097/00006231-200206000-00006. PMID: 12029209.

384: García A, Erdman SE, Xu S, Feng Y, Rogers AB, Schrenzel MD, Murphy JC, Fox JG. Hepatobiliary inflammation, neoplasia, and argyrophilic bacteria in a ferret colony. Vet Pathol. 2002 Mar;39(2):173-9. doi: 10.1354/vp.39-2-173. PMID: 12009055.

385: McAfee SL, Powell SN, Colby C, Spitzer TR. Dose-escalated total body irradiation and autologous stem cell transplantation for refractory hematologic malignancy. Int J Radiat Oncol Biol Phys. 2002 May 1;53(1):151-6. doi: 10.1016/s0360-3016(02)02743-8. PMID: 12007954.

386: Yoshimura M, Yamamoto T, Iso-o N, Imafuku I, Momose T, Shirouzu I, Kwak S, Kanazawa I. Hemiparkinsonism associated with a mesencephalic tumor. J Neurol Sci. 2002 May 15;197(1-2):89-92. doi: 10.1016/s0022-510x(02)00042-4. PMID: 11997073.

387: Kaim AH, Burger C, Ganter CC, Goerres GW, Kamel E, Weishaupt D, Dizendorf E, Schaffner A, von Schulthess GK. PET-CT-guided percutaneous puncture of an infected cyst in autosomal dominant polycystic kidney disease: case report. Radiology. 2001 Dec;221(3):818-21. doi: 10.1148/radiol.2213010445. PMID: 11719684.

388: Mochizuki T, Tsukamoto E, Kuge Y, Kanegae K, Zhao S, Hikosaka K, Hosokawa M, Kohanawa M, Tamaki N. FDG uptake and glucose transporter subtype expressions in experimental tumor and inflammation models. J Nucl Med. 2001 Oct;42(10):1551-5. PMID: 11585872.

389: Simmons JH, Riley LK, Franklin CL, Besch-Williford CL. Hamster polyomavirus infection in a pet Syrian hamster (Mesocricetus auratus). Vet Pathol. 2001 Jul;38(4):441-6. doi: 10.1354/vp.38-4-441. PMID: 11467479.

390: Zhao S, Kuge Y, Tsukamoto E, Mochizuki T, Kato T, Hikosaka K, Hosokawa M, Kohanawa M, Tamaki N. Effects of insulin and glucose loading on FDG uptake in experimental malignant tumours and inflammatory lesions. Eur J Nucl Med. 2001 Jun;28(6):730-5. doi: 10.1007/s002590100517. PMID: 11440033.

391: Hathway DE. Toxic action/toxicity. Biol Rev Camb Philos Soc. 2000 Feb;75(1):95-127. doi: 10.1017/s0006323199005447. PMID: 10740894.

392: Locatelli AJ, Marcos GM, Gómez MG, Alvarez SA, DeBenedetti LC. Comparing peritonitis in continuous ambulatory peritoneal dialysis patients versus automated peritoneal dialysis patients. Adv Perit Dial. 1999;15:193-6. PMID: 10682100.

393: Dujmović F. Nuklearna medicina danas [Nuclear medicine today]. Med Pregl. 1999 Sep-Oct;52(9-10):409-12. Croatian. PMID: 10624394.

394: Tonus C, Kolotas C, Appel P, Martin T, Zamboglou N, Nier H. Colorectales Carcinom--Behandlungsalternative durch intraoperative Radiotherapie mittels Afterloading-flab-Technik? Chirurg. 1999 Jan;70(1):43-7; discussion 48. German. doi: 10.1007/s001040050603. PMID: 10068822.

395: Miller WL, Geller DH, Auchus RJ. The molecular basis of isolated 17,20 lyase deficiency. Endocr Res. 1998 Aug-Nov;24(3-4):817-25. doi: 10.3109/07435809809032692. PMID: 9888582.

396: Schröder O, Trojan J, Zeuzem S, Baum RP. Limited value of fluorine-18-fluorodeoxyglucose PET for the differential diagnosis of focal liver lesions in patients with chronic hepatitis C virus infection. Nuklearmedizin. 1998;37(8):279-85. PMID: 9868710.

397: Willett BJ, Adema K, Heveker N, Brelot A, Picard L, Alizon M, Turner JD, Hoxie JA, Peiper S, Neil JC, Hosie MJ. The second extracellular loop of CXCR4 determines its function as a receptor for feline immunodeficiency virus. J Virol. 1998 Aug;72(8):6475-81. doi: 10.1128/JVI.72.8.6475-6481.1998. Erratum in: J Virol 1998 Oct;72(10):8460. PMID: 9658090; PMCID: PMC109811.

398: Hosie MJ, Broere N, Hesselgesser J, Turner JD, Hoxie JA, Neil JC, Willett BJ. Modulation of feline immunodeficiency virus infection by stromal cell-derived factor. J Virol. 1998 Mar;72(3):2097-104. doi: 10.1128/JVI.72.3.2097-2104.1998. PMID: 9499065; PMCID: PMC109504.

399: O'Doherty MJ, Barrington SF, Campbell M, Lowe J, Bradbeer CS. PET scanning and the human immunodeficiency virus-positive patient. J Nucl Med. 1997 Oct;38(10):1575-83. PMID: 9379195.

400: Mehrotra R, Khanna R, Yang TC, Kathuria P, Moore HL, Prowant BF, Nolph KD, Twardowski ZJ. Calculation of 6-hour D/P creatinine ratio from the 4-hour peritoneal equilibration test. The effect of dwell duration on the results. Perit Dial Int. 1997 May-Jun;17(3):273-8. PMID: 9237289.

401: Miller TR. The AAPM/RSNA physics tutorial for residents. Clinical aspects of emission tomography. Radiographics. 1996 May;16(3):661-8. doi: 10.1148/radiographics.16.3.8897630. PMID: 8897630.

402: Heaf J. CAPD adequacy and dialysis morbidity: detrimental effect of a high peritoneal equilibration rate. Ren Fail. 1995 Sep;17(5):575-87. doi: 10.3109/08860229509037622. PMID: 8570870.

403: Hombal SM, Dincsoy HP. Pasteurella multocida endocarditis. Am J Clin Pathol. 1992 Dec;98(6):565-8. doi: 10.1093/ajcp/98.6.565. PMID: 1462953.

404: Diamant YZ, Rimon E, Evron S. High incidence of preeclamptic toxemia in patients with polycystic ovarian disease. Eur J Obstet Gynecol Reprod Biol. 1982 Dec;14(3):199-204. doi: 10.1016/0028-2243(82)90097-1. PMID: 7160531.

405: GREEN JA, GOLDZIEHER JW. THE POLYCYSTIC OVARY. IV.LIGHT AND ELECTRON MICROSCOPE STUDIES. Am J Obstet Gynecol. 1965 Jan 15;91:173-81. PMID: 14258017.

406: NELSON JB. RESPONSE OF MICE TO REOVIRUS TYPE 3 IN PRESENCE AND ABSENCE OF ASCITES TUMOR CELLS. Proc Soc Exp Biol Med. 1964 Aug-Sep;116:1086-9. doi: 10.3181/00379727-116-29459. PMID: 14230352.

407: KITAMURA I, VAN HOOSIER G Jr, SAMPER L, TAYLOR G, TRENTIN JJ. CHARACTERISTICS OF HUMAN ADENOVIRUS TYPE 12 INDUCED HAMSTER TUMOR CELLS IN TISSUE CULTURE. Proc Soc Exp Biol Med. 1964 Jul;116:563-8. doi: 10.3181/00379727-116-29307. PMID: 14194601.

408: KETTLER LH. DIE HISTOLOGISCHE DIFFERENTIALDIAGNOSE AKUTER UND CHRONISCHER LEBERERKRANKUNGEN [THE HISTOLOGICAL DIFFERENTIAL DIAGNOSIS OF ACUTE AND CHRONIC LIVER DISEASES]. Z Arztl Fortbild (Jena). 1964 Jan 1;58:14-20. German. PMID: 14133208.

409: GRANBOULAN N, TOURNIER P, WICKER R, BERNHARD W. An electron microscope study of the development of SV40 virus. J Cell Biol. 1963 May;17(2):423-41. doi: 10.1083/jcb.17.2.423. PMID: 13963379; PMCID: PMC2106205.

410: FAWCETT DW. Electron microscope observations on intracellular virus-like particles associated with the cells of the Lucké renal adenocarcinoma. J Biophys Biochem Cytol. 1956 Nov 25;2(6):725-41. doi: 10.1083/jcb.2.6.725. PMID: 13398440; PMCID: PMC2224004.
